# Does milk intake promote prostate cancer initiation or progression via effects on insulin-like growth factors (IGFs)? A systematic review and meta-analysis

**DOI:** 10.1007/s10552-017-0883-1

**Published:** 2017-03-30

**Authors:** Sean Harrison, Rosie Lennon, Jeff Holly, Julian P. T. Higgins, Mike Gardner, Claire Perks, Tom Gaunt, Vanessa Tan, Cath Borwick, Pauline Emmet, Mona Jeffreys, Kate Northstone, Sabina Rinaldi, Stephen Thomas, Suzanne D. Turner, Anna Pease, Vicky Vilenchick, Richard M. Martin, Sarah J. Lewis

**Affiliations:** 1 School of Social and Community Medicine, University of Bristol, Bristol, UK; 2 MRC Integrative Epidemiology Unit (IEU), University of Bristol, Bristol, UK; 3 IGFs & Metabolic Endocrinology Group, School of Clinical Sciences at North Bristol, Southmead Hospital, BS10 5NB Bristol, UK; 4 Nuffield Department of Population Health, University of Oxford, Oxford, UK; 5 Cardiff University, Cardiff, UK; 6 CLAHRC West, University of Bristol, Bristol, UK; 7 International Agency for Research on Cancer, Lyon, France; 8 School of Oral and Dental Sciences,, University of Bristol, Bristol, UK; 9 Department of Pathology, University of Cambridge, Cambridge, UK; 10 National Institute for Health Research Biomedical Research Unit in Nutrition, Diet and Lifestyle, University Hospitals Bristol NHS Foundation Trust and the University of Bristol, BS2 8AE Bristol, UK

**Keywords:** Prostate cancer, Insulin-like growth factors, Milk, Mechanistic pathway, Systematic review, Meta-analysis

## Abstract

**Purpose:**

To establish whether the association between milk intake and prostate cancer operates via the insulin-like growth factor (IGF) pathway (including IGF-I, IGF-II, IGFBP-1, IGFBP-2, and IGFBP-3).

**Methods:**

Systematic review, collating data from all relevant studies examining associations of milk with IGF, and those examining associations of IGF with prostate cancer risk and progression. Data were extracted from experimental and observational studies conducted in either humans or animals, and analyzed using meta-analysis where possible, with summary data presented otherwise.

**Results:**

One hundred and seventy-two studies met the inclusion criteria: 31 examining the milk–IGF relationship; 132 examining the IGF–prostate cancer relationship in humans; and 10 animal studies examining the IGF–prostate cancer relationship. There was moderate evidence that circulating IGF-I and IGFBP-3 increase with milk (and dairy protein) intake (an estimated standardized effect size of 0.10 SD increase in IGF-I and 0.05 SD in IGFBP-3 per 1 SD increase in milk intake). There was moderate evidence that prostate cancer risk increased with IGF-I (Random effects meta-analysis OR per SD increase in IGF-I 1.09; 95% CI 1.03, 1.16; *n* = 51 studies) and decreased with IGFBP-3 (OR 0.90; 0.83, 0.98; *n* = 39 studies), but not with other growth factors. The IGFBP-3 -202A/C single nucleotide polymorphism was positively associated with prostate cancer (pooled OR for A/C vs. AA = 1.22; 95% CI 0.84, 1.79; OR for C/C vs. AA = 1.51; 1.03, 2.21, *n* = 8 studies). No strong associations were observed for IGF-II, IGFBP-1 or IGFBP-2 with either milk intake or prostate cancer risk. There was little consistency within the data extracted from the small number of animal studies. There was additional evidence to suggest that the suppression of IGF-II can reduce tumor size, and contradictory evidence with regards to the effect of IGFBP-3 suppression on tumor progression.

**Conclusion:**

IGF-I is a potential mechanism underlying the observed associations between milk intake and prostate cancer risk.

**Electronic supplementary material:**

The online version of this article (doi:10.1007/s10552-017-0883-1) contains supplementary material, which is available to authorized users.

## Introduction

Dairy consumption, and in particular milk, has been implicated as a risk factor for prostate cancer, although results are inconsistent. The World Cancer Research Fund (WCRF)/ American Institute for Cancer Research (AICR) concluded in their 2014 expert report that there is limited evidence that milk increases risk [1]: the synthesized results of 15 of the 21 studies identified as examining the association between dairy products and prostate cancer risk showed a 7% increased risk per 400 g of dairy products consumed per day (RR 1.07, 95% CI 1.02–1.12). However, observational studies have been hindered by milk consumption being recorded semi-quantitatively in some studies, with large differences between individuals in the same group, and therefore subject to attenuation of effects by measurement error. In addition, associations of milk intake with prostate cancer are susceptible to confounding by other diet and lifestyle factors.

Evidence to support the causality of observed positive associations of milk intake with prostate cancer could come from basic experimental studies. For example, a recent study [2] showed that milk stimulates the growth of prostate cancer cells in culture. In addition, evidence of underlying mechanisms by which milk causes prostate cancer may shed some light on whether observational findings are likely to be accurate. However, some way of systematically collating and synthesizing data from such disparate sources is required in order to avoid the selective interpretation of evidence.

Our overall objective in this work was to assemble the worldwide literature from human, animal, and genetic models to investigate whether any association between milk consumption and prostate cancer initiation or progression acts via the IGF pathway. The review was undertaken as a case study in support of a novel framework for reviewing mechanistic studies of exposures and cancer; the milk–prostate cancer association was not considered as the WCRF have already synthesised this literature. The framework was commissioned by WCRF UK, as an extension of their Continuous Update of the 2007 Report [1].

Several mechanisms have been hypothesised by which milk could promote prostate cancer. First, increased calcium intake in people with high milk consumption may suppress the conversion of 25(OH) vitamin D to 1,25(OH)2 vitamin D, which has antiproliferative effects on human prostate cancer cells [3]. Second, milk is a rich source of oestrogens [4], which are associated with prostate cancer, although the mechanism of action is poorly understood [5]. Furthermore, the nutritionally regulated (including by milk intake [6]) insulin-like growth factor (IGF) signaling pathway has been highlighted in a number of studies as a probable factor in prostate cancer (PCa) initiation and progression [7–9].

Relevant mechanistic studies will frequently have an intermediate phenotype (in this case the IGF system) rather than cancer as an outcome, or the intermediate phenotype as the exposure for a cancer outcome. Therefore, we separately identified studies that linked our exposure of interest (milk) to the intermediate phenotype, and studies that linked the same intermediate phenotype to prostate cancer initiation or progression. Data from each evidence stream (human, animal, and genetic models) were critically appraised using specific risk of bias (RoB) protocols and graded using the Grading of Recommendations Assessment, Development, and Evaluation (GRADE) tool. By combining the evidence from each study type and using strict RoB and GRADE protocols to ensure the inclusion of high-quality data only, our aim was to provide a comprehensive review of the milk–PCa relationship, focusing on IGF as a specific intermediate phenotype.

## Materials and methods

### Data sources

We carried out two searches to identify studies that investigated (i) associations between milk intake and IGF levels (milk–IGF); and (ii) associations between IGF levels and prostate cancer (PCa) outcomes (IGF–PCa). MEDLINE (1950–March 2014), EMBASE (1980–March 2014), BIOSIS (1969–March 2014) and CINAHL (1981–March 2014) were systematically searched. Searches were performed using key words (BIOSIS) or a combination of key words and subject headings (MEDLINE, EMBASE, and CINAHL). Full search terms are included in Supplementary Boxes 1–3.

### Inclusion and exclusion criteria

We included original articles published in peer-reviewed journals (including supplements and meeting abstracts); reviews, books, commentaries, and letters were excluded. We included randomized controlled trials (RCTs), case–control, cohort, non-randomized experimental, and case-series studies in humans, as well as all animal studies, with the exception of those that used a known carcinogen alongside the exposure. This decision was based on the relevance of carcinogenic-initiated cancers within the context of addressing whether milk causes prostate cancer initiation or its progression. We included both transgenic and xenograft animal models; although transgenic models were considered to be more relevant within a human context, xenograft models may give some insight into the biological plausibility of the mechanism of action. Animal studies that only presented cell line data or used hallmarks of cancer as the outcome [10] were analyzed separately; thus the results are not presented here. There were no language restrictions.

### Milk–IGF specific criteria

We included all papers that investigated associations of dairy and milk intake with IGFs and IGFBPs. Outcomes of interest were serum or plasma levels of IGF-I, IGF-II, and IGF binding proteins (IGFBP-1, -2, and -3). Exposures of interest were milk or milk products, including dairy protein or dairy product intake. Since different milk products contain different amounts of water and other substances, they were considered both separately and together so that any differences between the exposures could be observed. We included both RCTs and observational studies. The primary exposure of interest within this analysis was cow’s milk as a dietary environmental factor in relation to PCa risk; therefore, we excluded studies where the exposure was human breast milk, colostrum, soy milk or formula milk.

### IGF–Prostate Cancer specific criteria

We included all papers that measured the association between the IGF pathway and prostate cancer outcomes. Exposures of interest were serum or plasma levels of IGF-I, IGF-II, and IGF binding proteins (IGFBPs)-1, -2, and -3; expression levels of IGF-I, IGF-II, IGFBP-1, -2, and -3; IGF-I receptor (R), IGF-IIR genes and any other genes (or specific single nucleotide polymorphisms [SNPs]) with ‘IGF’ as part of the gene name and that were part of the IGF pathway. Outcomes of interest included prostate cancer incidence or prevalence, measures of progression (biochemical recurrence, local or distal metastases), and prostate cancer-specific mortality. We included studies using healthy or benign prostatic hyperplasia (BPH) control subjects. We excluded papers if PCa was not a defined outcome.

### Data extraction

After papers (milk–IGF and IGF–PCa) had been identified and collated, exact duplicates were excluded. Titles, author names, page numbers, years of publication, and journal names were analyzed to exclude any remaining duplicates that had different entries in multiple databases due to typographical errors or reference style. The abstracts of all remaining papers were retrieved and independently screened by two of four possible assessors (SH, VV, AP, MG); where no abstract was available, or if the abstract provided insufficient information to inform a screening decision, the full text was retrieved for review. Any discrepancies between the two assessors were resolved by a third assessor.

Data were independently extracted in duplicate for each study (SH, RL), with disagreements resolved by discussion. Data extracted for all study types included details of study or model design, study population (location and sample size), exposure or intervention, outcome, statistical measure (including details of any model adjustments), and effect estimates (including mean values, standard deviation [SD], P values, and odds ratios [OR] with any corresponding 95% confidence intervals [CI] as a continuous measure or by quantiles). For each study type (human studies [milk–IGF, IGF–PCa] or animal models [IGF–PCa]), specific data were also collected (see **Box** **1**).

**Table:** **Box 1** Differences in data extracted for each study type

Milk–IGF	IGF–prostate cancer
Human studies	
Study design: RCT, cross-sectional, cohort Intervention: milk, dairy product, dairy protein intake Outcome: type of IGF, IGFBP Measure of exposure (units) Sample size Average age of subjects Percentage of males in study Method of diet assessment Length of follow-up Ethnicity	Study design: cohort, nested case control, case control Retrospective or prospective ascertainment of outcome with respect to exposure measurement Intermediate phenotype: type of IGF and IGFBP, name of genes/SNPs/allele/number of repeats Study category: IGF levels, genetic, supporting evidence^a^ Control source (population, hospital, population-based cohort, trial-based cohort) Control type (healthy, BPH, mixed BPH, and healthy) Sample type (serum, plasma) Method of exposure measurement Age of cases and controls (including where possible, age at diagnosis) Time between sample collection and analysis PSA-detected or clinically detected study populations Mid-year of recruitment Study name Ethnicity
Animal studies	
All IGF-PCa animal studies	
Model design: transgenic, xenograft Outcome: tumor biometrics (various), hallmarks of cancer [10] Type of experimental and control models used Length of follow-up	

^a^Studies which did not compare IGF biomarkers or genotypes in cases vs controls or against progression or mortality, but nevertheless may provide evidence on the role of the IGF pathway in prostate cancer. Examples include studies examining the association between prostate cancer risk and loss of imprinting in genes or haplotypes (as opposed to single nucleotide polymorphisms), and studies looking at progression of prostate cancer after treatment

### IGF–Prostate cancer studies

To minimize the risk of reverse causation (i.e., the secondary effects of PCa on IGF levels rather than the causal effects of IGF levels on PCa), we pragmatically classified human studies assessing IGF–PCa as prospective if there was a mean of 2 years or more between the collection of samples and diagnosis; otherwise they were classified as retrospective. Where the time between sample collection and diagnosis was unclear, we conservatively classified the studies as retrospective. This classification may differ from how the studies were classified by the authors in the original publication.

For papers presenting data in several ways the order of choosing the data to be synthesized was (i) reported coefficients (log odds ratio [OR] or risk ratio per unit increase in exposure); (ii) quantile data (ORs stratified by quantiles of IGF) and (iii) continuous data presented as mean or median differences. We extracted data that were fully adjusted up to, but not including, mutual adjustment for a different growth factor (i.e., IGF-I adjusted for IGFBP-3 and vice versa). If more than one publication or study presented data on the same IGF biomarker from the same cohort, we extracted data from the study with the largest sample size.

During extraction, we divided human IGF–PCa studies into three categories that provided data on (i) the association between circulating levels of IGF or IGFBPs, ‘IGF level’ studies and prostate cancer outcome; (ii) IGF or IGFBP genes or SNPs, ‘genetic’ studies; and (iii) studies which provided neither (i) or (ii), but presented data relevant to the relationship between the IGF pathway and PCa outcomes, termed as ‘supporting evidence.’ Studies examining free IGF levels, as opposed to total IGF levels, were not amenable to meta-analysis as they could not be combined with total IGF levels and there were too few papers to meta-analyze alone. As such, studies examining free IGF levels were included in supporting evidence.

### Milk–IGF studies

We extracted *p* values and sample sizes from all studies. If *p* values were not presented, 95% CIs and effect estimates were used to estimate the *p* value. Data for males were extracted in preference to females (because the ultimate outcome of interest was prostate cancer), followed by combined data, then female only data.

### Statistical analyses

#### Milk–IGF data

The main difficulty in combining all studies that examined the relationships of milk, dairy products, and dairy proteins with IGFs and IGFBPs was the degree of heterogeneity between these studies. Study designs ranged from RCTs conducted over decades to short-term observational studies; the exposures (milk, dairy protein, and dairy products) were both different and measured differently between studies; and study participants varied in age, ethnicity, and location. In addition, effect estimates were provided in different formats, such as percentage increases or ORs, often with too little information to estimate a standardized effect estimate.

We generated albatross plots [11] for each outcome to best interpret the results. An albatross plot is a scatter plot of study sample sizes against two-sided *p* values, with results separated according to the observed direction of effect. The albatross plot allows the *p* values to be interpreted in the context of the study sample size. Small studies appear toward the bottom of the plot and large studies toward the top. Different exposures are drawn using different markers to facilitate identification of subgroup effects. Effect contours are superimposed on the plot to give an indication of the magnitude of effect both for individual studies, and for the association as a whole.

To provide additional information, a meta-analysis of P values was conducted using Stouffer’s *Z* score method of combining P values [12]; the one-tailed P value for the most common direction of effect across studies for each IGF was used to calculate the one-tailed combined *p* value across studies for each IGF.

### IGF–Prostate cancer data

#### Types of data used

To compare data from multiple studies that examined relationships of growth factors with prostate cancer, we calculated the log OR per standard deviation (SD) increase in exposure, as previously described in Rowlands et al. [8]. The log OR per unit increase in exposure for studies presenting results as a difference in means between cases and controls was calculated using the method presented by Chêne and Thompson [13]. For studies presenting results within quantiles of exposure, the mean or median exposure was used in each quantile (if reported), and the log OR per unit increase in exposure was calculated using the method presented by Greenland and Longnecker [14], using the ‘glst’ command in Stata [15]. When the mean or median in each quantile was not reported but instead a range of exposures in each group was, the mean exposure was estimated in each quantile using the method presented by Chêne and Thompson [13] (assuming a normal distribution of the exposure in the population). When no mean, median or range was reported, a normal distribution was assumed based on the mean and SD of the group used to generate the quantiles (usually the controls). This distribution was used to calculate the quantile range, and thus the mean of each quantile. If only subgroup analyses were presented and not an overall case versus control group analysis, the subgroups were combined statistically by calculating pooled means and SDs. The log ORs per unit increase and their standard errors were converted to a per SD increase by multiplying by the SD of the exposure. For quantile data, the SD was calculated using the method presented by Chêne and Thompson [13].

As in the Rowlands meta-analysis [8], a linear relationship between growth factors and prostate cancer was assumed, which can result in a different OR to a highest versus lowest quantile analysis. However, this method allowed the use of the middle quantiles of data, increasing the amount of available data.

#### Random effects and fixed effect meta-analyses

We performed random effects and fixed effect meta-analyses on all growth factors to calculate summary OR estimates, using the ‘metan’ Stata command [16]. We calculated the I^2^ statistic as a quantitative measure of the degree of inconsistency across studies [17]. Small study effects were assessed by inspection of funnel plots and computation of Egger and Begg tests [18, 19].

There was substantial inconsistency across studies in the IGF–PCa analysis (*I* ^*2*^ > 65% for all growth factors). Therefore, our primary results are from random effects models throughout this paper; we also present fixed effect results for comparison.

#### Subgroup analyses

To investigate whether growth factors were associated with advanced prostate cancers, we conducted a separate meta-analysis using data from studies that examined this outcome. The definition of advanced prostate cancers varied across studies; the combined definition of ‘advanced’ thus extends to non-localized cancers, Gleason score 7+ cancers, metastasized cancers, ‘high grade’ cancers (the definition of which varied between studies) or ‘aggressive’ cancers (which were a combination of advanced stage and high grade).

We produced forest plots for all growth factor and both prostate cancer outcome (all and advanced) analyses; these plots were stratified by prospective and retrospective studies, and PSA-detected and clinically detected studies. The difference between subgroup estimates was explored using the method of Altman and Bland [20].

### Genetic data

Although many studies reported associations between various SNPs and prostate cancer, only two genetic variants were examined by a sufficient number of studies to allow us to conduct meta-analyses: IGF-I CA repeats, and IGFBP-3 -202 A/C polymorphisms. We included studies which presented results in a combinable way: for IGF-I CA repeats, 19/X and X/X were both compared against 19/19 repeats in separate analyses, where X was anything other than 19. For IGFBP-3 -202 A/C polymorphism, A/C and C/C were both compared against A/A in separate analyses. If data were presented in the opposite direction, i.e., A/C and A/A against C/C, we transformed the results to allow these to be combined with other data. The meta-analyses for these genotypes were conducted in the same manner as in the IGF–PCa studies with forest and funnel plots produced, but not grouped by prospective/retrospective, as an individual’s genotypes do not change over time, and therefore should not be susceptible to reverse causation.

### Risk of bias (RoB) and GRADE assessments

Due to the variety of studies included within the meta-analysis and the need for consistent risk of bias assessment, we developed a tool to determine the overall risk of bias for each study using the categories of assessment from a draft of the ROBINS-I tool [21], and the questions to aid in assessing risk of bias from the CASP case–control and cohort questionnaires [22, 23]. Bias was assessed in six categories: Confounding, selection of participants, missing data, outcome measurement, exposure measurement, and results’ reporting. Each category contained questions designed to help assess the risk of bias; these varied depending on the study type (e.g., animal, human, genetic) and design (e.g., cohort, RCT, case–control) (Supplementary Box 4). An overall and category-specific risk of biases were assigned; either low, moderate, serious, critical, or unclear.

On the basis that circulating IGF levels increase with age from birth to young adulthood and then gradually decline into old age, any studies which did not age-match cases and controls or adjust for age within the analysis, and presented a difference of 5 or more years between the mean age of cases and controls, were categorized as critical RoB due to confounding and excluded from the analysis. In addition, all observational studies that investigated serum levels of IGF in relation to PCa risk were given a moderate RoB status as a minimum, as it is unlikely that all confounding factors could be fully controlled for within observational designs. The majority of the human IGF–PCa cohort papers did not provide information on missing data with regards to patients lost at follow-up, therefore these papers were considered to have at least a moderate RoB due to missing data.

All milk–IGF studies that used food frequency questionnaires, or used diet assessments involving information recall, were also given a moderate RoB rating as a minimum due to measurement error when dietary recall is used as the only measure of exposure.

For milk studies, if only one RoB sub-category was found to be unclear, the level of bias the sub-category could have caused was taken into consideration. For example, confounding could have a large impact on risk of bias, and therefore an unclear risk of bias in confounding would be assumed to be serious risk of bias. Conversely, results’ reporting has a relatively smaller impact on risk of bias, and therefore unclear risk of bias would be assumed to be moderate risk of bias. The overall risk of bias for a study was based on the sub-category with the largest risk of bias. Studies determined to have a critical risk of bias were excluded before analysis.

The GRADE rating for each exposure/outcome pair was calculated as per the GRADE protocol [24]. The RoB status of each paper was used to inform the GRADE rating for each collection of papers within a study category (e.g., animal IGF–PCa). The overall GRADE rating was used to provide a measure of overall quality of the evidence provided by the results.

## Results

**Fig. 1:**
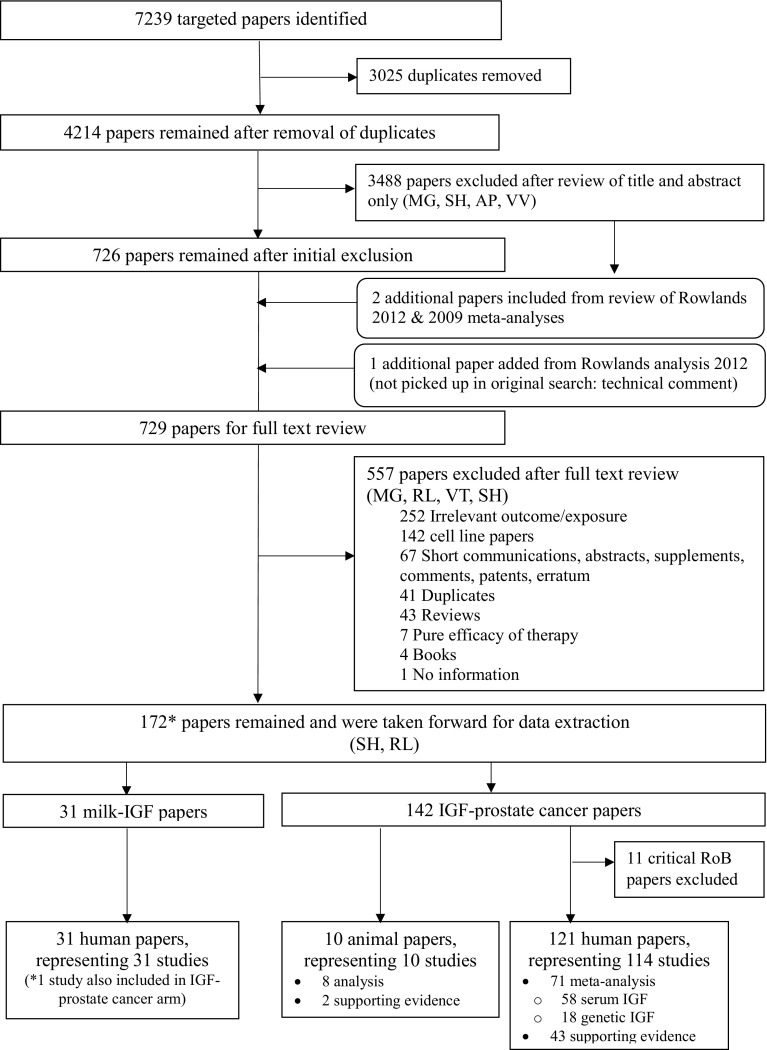
Flow diagram depicting the inclusion and exclusion process during the meta-analysis and the number of papers categorized into each study type for milk–IGF and IGF–PCa

Systematic searches of all four online databases identified 7,239 papers; 3,025 cross-database duplicates were removed, leaving 4,214 papers. After abstract screening, 728 papers remained for full text review, including one additional paper from Rowlands et al. [8] that was not identified within the original search [25]. Of these, 172 papers met the inclusion criteria and were taken forward for data extraction (Fig. 1): 31 papers (representing 31 independent studies) examining the milk–IGF relationship; 132 papers (representing 125 studies) examining the IGF–prostate cancer relationship in humans; and ten papers (representing ten studies) examining the IGF–prostate cancer relationship in animals. One study had data relevant to both milk–IGF and IGF–PCa analyses [26].

None of the 31 milk–IGF studies were excluded due to RoB, and all were included in at least one albatross plot and narrative synthesis of different IGFs, except for two studies [27, 28] that looked at qualitatively different outcomes, which were excluded from the albatross plots and considered separately in the narrative synthesis. The total number of studies looking at each type of IGF was as follows (the two studies excluded from albatross plots shown in brackets): IGF-I, *n* = 28 (+2); IGF-II, *n* = 2 (+1); IGFBP-1, *n* = 2; IGFBP-2, *n* = 2 (+1); IGFBP-3, *n* = 15 (+2). Three studies stratified results by ethnicity; the ethnicity subgroups were considered as separate data points in the albatross plots [29–31].

Of the studies included in the milk–IGF analysis, two had serious RoB (both retrospective cross-sectional studies) [32, 33]; 18 had moderate RoB (two prospective cohort studies; 16 retrospective cross-sectional studies) [27, 31, 34–49]; one had low RoB (non-randomized trial) [30]; and nine had unclear RoB (four RCTs, one non-randomized trial, three prospective cohorts, and one retrospective cross-sectional study) [28, 29, 50–57].

No animal milk–IGF studies were included due to irrelevant exposures or outcomes of interest; the majority of animals were exposed to colostrum intake within the first 6 months of life as part of study designs.

Of the 132 human IGF–PCa studies, 89 had serum level IGF data available for meta-analysis; the remaining 43 studies were considered as supporting evidence as they did not contain any data amenable to meta-analysis [58–100]. One study was considered as supporting evidence since it presented data for free IGF-I in relation to PCa risk, rather than total serum levels of IGF-I [75].

Of the 89 studies with data for meta-analysis, 16 had serious RoB (all retrospective) [101–116], 47 had moderate RoB (23 prospective, 24 retrospective) [8, 26, 34, 117–160], eight had low RoB (all genetic only) [161–168] and seven had unclear RoB (five genetic only, two retrospective) [25, 169–174]. Eleven observational studies were excluded from the meta-analysis due to critical RoB (one prospective, one genetic, and nine retrospective [175–185]).

Seven studies were excluded from the meta-analysis as they presented data from the same group of participants with the same exposure and outcome variables as other included studies that contained more information (e.g., more participants) [34, 122, 153, 156–160], leaving 71 unique studies for meta-analysis; 58 studies examining serum IGF levels (IGF-I: 51 studies; IGF-II: 10 studies; IGFBP-1: 4 studies; IGFBP-2: 6 studies; IGFBP-3: 39 studies; IGF-I and IGFBP-3 Advanced PCa: 12 studies) and 18 examining IGF genetic data (IGF-I (CA)n repeats: 5 studies; IGFBP-3 -202 A/C SNP: 8 studies; other SNPs: 11 studies). Three papers did not contain enough information in the paper to calculate an OR [25, 151, 174]; therefore data from the previous Rowlands meta-analysis were used [8], as Rowlands contacted the study authors for more information as part of the data extraction protocol.

Eight animal IGF–PCa studies were included within the animal analysis [186–193], and two animal studies were included in supporting evidence [194, 195]; these studies did not have enough information to extract an effect estimate or P value. The overall risk of bias in all animal studies was unclear.

**Fig. 2:**
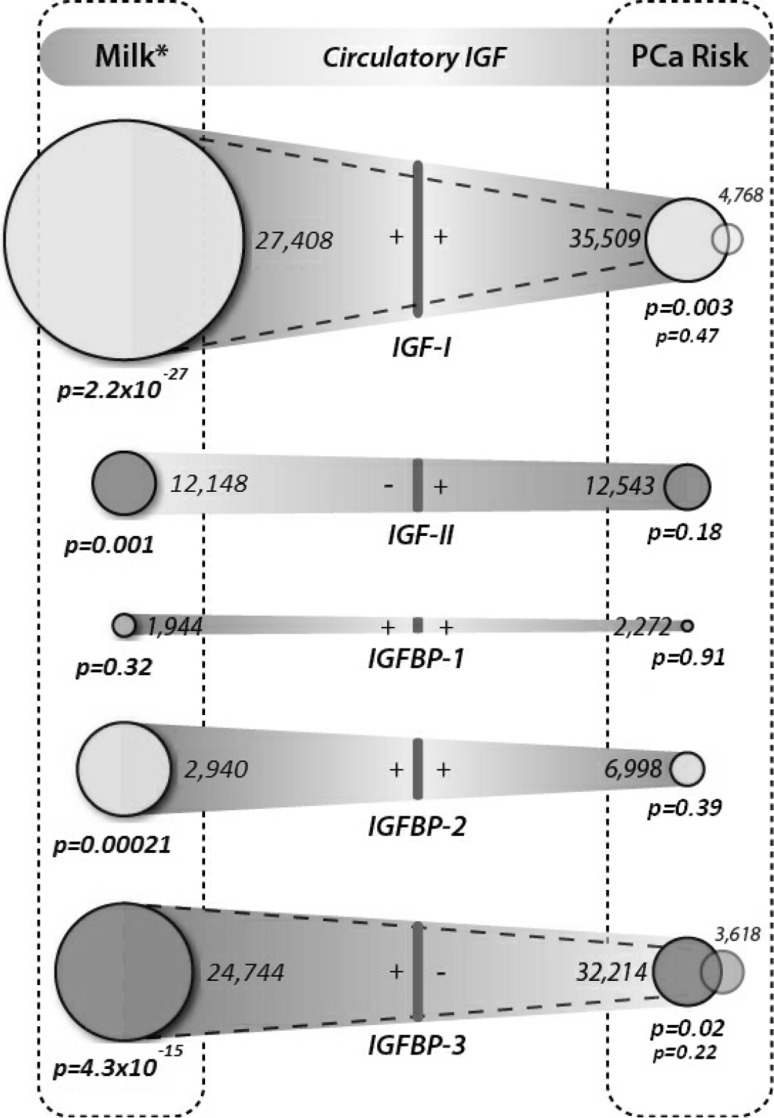
Infographic to illustrate overall associations between milk intake and PCa risk (including advanced risk for IGF-I and IGFBP-3). Notes: *numbers* next to the *circles* indicate the total number of participants across all studies, the size of each circle is proportionate to the *p* value (*larger circles* indicate lower *p* values), the “*+*” and “*−*” symbols indicate the direction of effect, the two *semi-transparent circles* in IGF-I and IGFBP-3 PCa Risk indicate advanced PCa risk (with associated *p* values the lower of the two *p* values), and *p* values are all calculated using Stouffer’s *Z* score method of combining *p* values. *Milk is used as a collective term for milk, dairy products and dairy proteins

An infographic displaying the main results of the human milk to IGF and IGF to PCa studies is presented in Fig. 2, showing the combined P values for the associations between milk and IGFs, and IGFs and PCa risk (including advanced PCa risk), the direction of effect and the total number of participants from all studies.

### Milk–IGF: human studies

Table 1 shows data extracted from studies examining the milk–IGF association. In general, studies examined the quantity of milk or dairy consumed through a food record or food frequency questionnaire, and used servings/weight of milk/dairy consumed per day/week, as the exposure (either categorically or continuously). The outcome (IGF levels) was measured using blood draws. The majority of studies were cross-sectional; prospective studies had a follow-up ranging from 1 week to 65 years. Interventional studies generally supplemented the diet of the participants, rather than depriving one group of milk/dairy. The age range of participants when the outcome was measured varied between 2 and 86 years; the majority of studies focussed on Caucasians.

**Table 1:** Studies investigating the association between milk, dairy protein, and dairy products and IGF-I, stratified by study type and ordered by year of publication

Author (year)	Intervention or exposure	Length of follow-up	Total sample size	Age of subjects (years)	Ethnicity (% male)	Diet assessment	Measure	Effect estimate	Overall risk of bias
*RCTs*									
Cadogan (1997) [50]	Milk (1 pint supplement/day) versus usual diet	18 months	82	12.2	Caucasian (0)	7 days weighed food record	Difference in IGF-I between intervention and control	*p* = 0.023; significantly higher IGF-1 levels in intervention	Unclear
Heaney (1999) [51]	Milk supplement (3 × 8 oz/day) versus no supplement	12 weeks	204	65.2	Caucasian (35.3)	3 days food record	Difference in IGF-I between baseline and follow-up in intervention and control	*p* < 0.001; IGF-I increased in intervention (+12 ng/ml), not in control (−2.0 ng/ml)	Unclear
Ben-Shlomo (2005) [28]	Milk supplement (NS) versus no supplement	25 years	644	25	Caucasian (53.6)	Questionnaire	Difference in IGF-I between intervention and control	*p* = 0.01; *r*= −9.5 (95% CI −16.6−2.27)	Unclear
Zhu (2005) [52]	Ca milk (330 ml/sch.day); CaD milk (330 ml/sch.day) vs no supplement	12; 24 months	606	10	Asian (0)	n/a	Baseline versus 24-month follow-up (Ca and CaD milk)	*p* < 0.001, significantly higher IGF-1 levels in both Ca and CaD milk compared to control	Unclear
*Non-randomized experimental studies*									
Hoppe (2004) [41]	Milk (1.5 l of skimmed milk/d) versus low-fat meat intake (250 g/day)	1 week	24	8	Caucasian (100)	FFQ	Baseline versus 24-month follow-up	*p* < 0.001; 19% in increase IGF-1 at 24 months in milk intake arm	Unclear
Rich-Edwards (2007) [30]	Mongolians: 710 ml milk/d for 1 month versus usual diet USA girls: 710 ml low-fat (2%) milk/d for 1 week versus macronutrient substitute for 1 week	1 month; 1 week	46 28	7.6	Asian (50)	7 days FFQ	Mongolia pre- vs postintervention USA substitute versus milk	Mongolians: *p* < 0.0001, significantly higher IGF-1 levels in intervention USA intervention: *p* = 0.35	Low
*Prospective studies (cohort)*									
Colangelo (2005) [29]	Milk (servings/day): (ethnic subgroups)	8 years	459 682	31.5 32.5	Black Caucasian (100)	FFQ (quantatative with interviewer)	Per quantile change in milk servings/day	Blacks: IGF-1 *p* = 0.05, increase in IGF-1 Caucasian: IGF-1 *p* = 0.31	Unclear
Martin (2007) [27]	Milk & milk products (g)	65 years	727	5.8 (baseline)	Caucasian (46)	7 days household inventory at baseline; FFQ at follow-up	Percentage change in IGF-I per SD	*p* = 0.06; −3.2% (95% CI −6.5, 0.07)	Moderate
Hrolfsdottir (2013) [54]	0–150 ml milk/day versus > 150-600 ml milk/day	20 years	436	Gest. wk30 (baseline)	Caucasian (51.9)	Questionnaire	Percentage change in IGF-I per SD	*p* = 0.12; 8.3% (95% CI −2.2, 20.0)	Unclear
Joslowski (2013) [55]	Dairy protein intake (tertiles)	>18 years of age	213	9–15 (baseline)	Caucasian (45.6)	3 days weighed diet records (x2)	*p* value for trend in linear regression models	*p* > 0.9	Unclear
Tsilidis (2013) [34]	Dairy protein intake (% energy)	NS	4105	60–69	Caucasian (100)	Questionnaire	Per tertile change in circulating IGF-1 and IGFBP-3 concentrations by dietary protein sources	*p* = 0.01, increase in IGF-1	Moderate
*Retrospective studies (cross-sectional)*									
Signorello (2000) [56]	Dairy products intake (g)	1 year	153	70–74	Caucasian (100)	FFQ (interviewer administered)	Percentage change in IGF-1 is per one quintile intake of dairy products	*p* = 0.57; −1.7(95% CI −7.4, 4.4)	Unclear
Mucci (2001) [35]	Dairy products (1 serving/day increment)	1 year	112	67.7	Caucasian (100)	FFQ	Percentage change in IGF-I per 1 serving/day increment	p = 0.41; 2.4% (95% CI −3.2, 8.3)	Moderate
Ma (2001) [36]	Skim/low-fat milk (8 oz glasses)	18 weeks	318	40–84	Caucasian (100)	19-item food report	*p* value for trend in tertiles of skim/low-fat milk intake	*p* = 0.0003, increase in IGF-1	Moderate
Holmes (2002) [37]	Milk intake (servings/day)	NA	1037	50.5	Caucasian (0)	FFQ	*p* value for trend in quartiles of milk intake	*p* = 0.01, increase in IGF-1	Moderate
Giovannucci (2003) [38]	Milk intake (1 serving increment/day)	NA	753	41–86	Caucasian (100)	Semi-quantitative FFQ	Change in plasma IGF-1 per 1 serving/day increment	*p* < 0.05; Δ 5.4 ng/ml, increase in IGF-1	Moderate
Gunnell (2003) [39]	Dairy products (g/week)	NA	344	62.2	Caucasian (100)	FFQ	Change in IGF-1 per SD increase	*p* = 0.09; *r* = 4.4 (95% CI −0.8, 9.7)	Moderate
DeLellis (2004) [40]	Total milk intake (g/1000 kcal/day)	NA	490	65	Caucasian (100)	Questionnaire	P value for trend in quartiles of total dairy intake (g/1000 kcal/day)	*p* = 0.66	Moderate
Hoppe (2004) [41]	Milk intake (g/day)	1 week	90	2.5	Caucasian (60)	Questionnaire	Change in IGF-1 per unit increase in milk intake	*p* = 0.045; *b* = 0.049 (SE 0.024)	Moderate
Larsson (2005) [57]	Total milk (g/day)	1 year	226	60.5	Caucasian (100)	24 h telephone interviews (x14)	Difference in IGF-I serum concentration per SD	*p* = 0.61; *b* = −1.7 (95% CI −8.2, 4.8)	Unclear
Morimoto (2005) [42]	Milk (servings/week)	NA	333	59.8	Caucasian (40.2)	Questionnaire	Per quantile change in milk servings/day	*p* < 0.05, increase in IGF-1	Moderate
Rogers (2006) [43]	Milk intake; dairy product intake (g)	NA	744	7–8	Caucasian (54.3)	3 days unweighted diet record	Percentage change in IGF-1 per 100 g increase in cows milk/dairy product	*p* = 0.24; 1.11 (95% CI 20.70, 2.94)	Moderate
McGreevy (2007) [31]	Dairy (servings/day)	NA	233	61 55	Caucasian Black (100)	2000 Brief block questionnaire	Per quantile change in milk servings/d	Caucasian: *p* = 0.82 Black: *p* = 0.03, increase in IGF-1	Moderate
Norat (2007) [44]	Milk & milk beverages (NS)	12 months	2109	54.5	Caucasian (0)	Questionnaire	P value for trend in quintiles of milk intake	*p* = 0.007, increase in IGF-1	Moderate
Budek (2007) [45]	Milk intake (g/day)	NA	56	8.1	Caucasian (100)	3 days weighed food record	Change in IGF-1 per unit increase in milk/dairy protein intake	*p* = 0.03; *b* = 0.05, increase in IGF-1	Moderate
Crowe (2009) [46]	Dairy protein intake (% energy)	NA	1142	59.9	Caucasian (100)	Questionnaire	P value for trend in quintiles of dairy protein intake (%)	*p* < 0.001, 2.4% increase in IGF-1	Moderate
Esterle (2009) [32]	Milk intake (ml/day)	NA	98	15.7	Caucasian (0)	7 days food recall, nutritionist	Difference between lowest (<55) and highest (>260) tertiles of milk intake	*p* < 0.015, increase in IGF-1	Serious
Maruyama (2009) [47]	Milk 3–4 servings/week plus	NA	10,350	63	Asian (100)	FFQ	*p* value for trend in quartiles of milk intake	*p* < 0.001, increase in IGF-1	Moderate
Young (2012) [48]	Dairy products (g)	12 months	1798	62	Caucasian (100)	FFQ (12 months)	Percentage change in IGF-I per SD	*p* < 0.001; 4.88% (95% CI 2.52, 7.23)	Moderate
Thorisdottir (2013) [33]	Dairy protein intake (% energy)	6 years	137	6	Caucasian (0)	3 days weighed food record	Dairy protein positive predictor of IGF1 in 6 year old girls	*p* = 0.0002; Δ 5.4 ug/l (95% CI 2.5, 8.2), increase in IGF-1	Serious

*FFQ* food frequency questionnaire, *RCT* randomized-controlled trial, *NS* not stated (with regards to unit of measurement)

Data extracted from the 31 milk–IGF studies were unsuitable for standard meta-analysis methods of combining effect sizes due to high levels of heterogeneity and differences in effect estimate formats (Tables 1, 2, 3). The studies investigated a range of exposures (including milk, milk beverages, dairy protein, and dairy products) that were quantified by a number of measures (such as density intake [g/1,000 kcal/day], percentage of energy intake and servings per day and grams per day).

**Table 2:** Studies investigating the association between milk, dairy protein, and dairy products and IGFBP-3

Author (year)	Intervention or exposure	Length of follow-up	*n*	Age of subjects (years)	Ethnicity (% male)	Diet assessment	Measure	Effect estimate	Overall risk of bias
*RCTs*									
Ben-Shlomo (2005) [28]	Milk supplement (NS) versus no supplement	25 years	644	25	Caucasian (53.6)	Questionnaire	Difference in IGFBP-3 between intervention and control	*p* = 0.55; *r* = −0.07 (95% CI −0.30, 0.17)	Unclear
*Non-randomized experimental studies*									
Hoppe (2004) [41]	Milk (1.5 l of skimmed milk/d) versus low-fat meat intake (250 g/day)	1 week	24	8	Caucasian (100)	FFQ	Baseline versus 24-month follow-up	*p* < 0.05, 8% increase in IGFBP-3 at 24 months in milk intake arm	Unclear
*Prospective studies (cohorts)*									
Colangelo (2005) [29]	Milk (servings/day) (ethnic subgroups)	8 years	459 682	31.5 32.5	Black Caucasian (100)	FFQ (quantitative with interviewer)	Per quantile change in milk servings/day	Blacks: *p* = 0.14 Caucasian: *p* = 0.84	Unclear
Martin (2007) [27]	Milk & milk products (g)	65 years	727	5.8 (baseline)	Caucasian (46)	7 days household inventory at baseline; FFQ at follow-up	Change in IGFBP-3 per SD	*p* = 0.5; −28.1 (95% CI 101.1,44.9)	Moderate
Tsilidis (2013) [34]	Dairy protein intake (% energy)	NA	4,104	60–69	Caucasian (100)	Questionnaire	Per tertile change in circulating IGFBP-3 concentrations by dietary protein sources	*p* = 0.32	Moderate
*Retrospective studies (cross-sectional)*									
Ma (2001) [36]	Skim/low-fat milk (8 oz glasses)	18 weeks	318	40–84	Caucasian (100)	19-item food report	*p* value for trend in tertiles of skim/low-fat milk intake	*p* = 0.004, increase in IGFBP-3	Moderate
Holmes (2002) [37]	Milk intake (servings/day)	NA	1,037	50.5	Caucasian (0)	FFQ	*p* value for trend in quartiles of milk intake	*p* = 0.37	Moderate
Giovannucci (2003) [38]	Milk intake (1 serving increment/day)	NA	753	41–86	Caucasian (100)	Semi-quantitative FFQ	Change in plasma IGFBP-3 per 1 serving/day increment	*p* < 0.05; Δ 64 ng/ml, increase in IGFBP-3	Moderate
Gunnell (2003) [39]	Dairy products (g/week)	NA	344	62.2	Caucasian (100)	FFQ	Change in growth factor per SD increase	*p* = 0.96; 3.9 (95% CI −139.4, 147.3)	Moderate
DeLellis (2004) [40]	Total milk intake (g/1000 kcal/day)	NA	490	65	Caucasian (100)	Questionnaire	*p* value for trend in quartiles of total dairy intake (g/1000 kcal/day)	*p* = 0.16	Moderate
Morimoto (2005) [57]	Milk (servings/week)	NA	333	59.8	Caucasian (40.2)	Questionnaire	Per quantile change in milk servings/days	*p* = 0.37	Moderate
Rogers (2006) [42]	Milk intake (g)	NA	744	7–8	Caucasian (54.3)	3 days unweighted diet record	Percentage change in IGFBP-3 per 100 g increase in cows milk/dairy products	*p* = 0.029; 1.82 (95% CI 0.20–3.36), increase in IGFBP-3	Moderate
Norat (2007) [44]	Milk & milk beverages (NS)	12 months	2,109	54.5	Caucasian (0)	Questionnaire	*p* value for trend in quintiles of milk intake	*p* = 0.06 (increase in IGFBP-3)	Moderate
Budek (2007) [45]	Milk intake(g/day)	NA	56	8.1	Caucasian (100)	3 days weighed food record	Change in IGFBP-3 per unit increase in milk/dairy protein intake	*p* = 0.15; b = 0.51	Moderate
Crowe (2009) [46]	Dairy protein intake (% energy)	NA	1,142	59.9	Caucasian (100)	Questionnaire	Mean change in serum IGF concentration per SD increment in dairy protein intake	*p* = 0.19; 0.42 (95 %CI −0.21, 1.06)	Moderate
Maruyama (2009) [47]	Milk 3–4 servings/week plus	NA	10,350	63	Asian (100)	FFQ	*p* value for trend in quartiles of milk intake	*p* < 0.001, increase in IGFBP-3	Moderate
Young (2012) [48]	Dairy products (g)	12 months	1,798	62	Caucasian (100)	FFQ (12 months)	Percentage change in IGFBP-3 per SD	Dairy products: *p* = 0.24	Moderate

*FFQ* food frequency questionnaire, *RCT randomized-controlled trial, NS not stated (with regards to unit of measurement)*

**Table 3:** Studies investigating the association between milk, dairy protein, and dairy products and IGF-2, IGFBP-1, and IGFBP-2

IGF	Author (year)	Intervention or exposure	Length of follow-up	*n*	Age of subjects (years)	Ethnicity (% male)	Diet assessment	Measure	Effect estimate	Overall risk of bias
*Prospective studies (cohorts)*										
IGF-II	Martin (2007) [27]	Milk & milk products (g)	65 years	726	5.8 (baseline)	Caucasian (46)	7 days household inventory at baseline; FFQ at follow-up	Change in IGF-2 per SD of milk and milk beverages	*p* = 0.5; −6.4 (95% CI −24.3, 11.4)	Moderate
IGFBP-2									*p* = 0.3; −1.9 (95% CI −5.8, 2.1)	
*Retrospective studies (cross-sectional)*										
IGFBP-1	DeLellis Henderson (2007) [49]	Total dairy intake density (g/1000 kcal/days)	NA	802	60.66	Caucasian (56.1)	Questionnaire	Trend in mean plasma IGFBP-1 per quantile of total dairy intake density	*p* = 0.388	Moderate
IGF-II	Maruyama (2009) [47]	Milk 3–4 servings/week plus	NA	10,350	40–79	Asian (100)	FFQ	*p* value for trend in quartiles of milk intake	*p* < 0.001, increase in IGF-2	Moderate
IGFBP-1	Crowe (2009) [46]	Dairy protein intake (% energy)	NA	1,142	59.9	Caucasian (100)	Questionnaire	Trend in quintiles of dairy protein intake (%)	*p* = 0.83; −0.45 (95% CI -4.43, 3.69)	Moderate
IGFBP-2									*p* = 0.016; −3.45 (95% CI −6.20 −0.61);	
IGF-II	Young (2012) 48]	Dairy protein (g)	12 months	1,798	62	Caucasian (100)	FFQ (12 month)	Percentage change in IGF-2 per SD	*p* = 0.28	Moderate
IGFBP-2									*p* < 0.−3.43% (95% CI −5.92, −0.87)	

*FFQ food frequency questionnaire*

Albatross plots were used to assimilate the data and visual inspection was used to provide an estimated standardized effect estimate and a range of possible effect estimates for each outcome (Table 4). The effect estimates are not precise and are intended only as an indication of the magnitude of the effect estimate; a range of possible estimates is provided to highlight this fact. We present all standardized effect estimates for the milk–IGF association as the standard deviation (SD) increase in IGF protein per one standard deviation increase in the measure of exposure. One SD of milk varies across studies, especially as studies differ in presentation of milk units (e.g., grams per day, milliliters per day, portions per day). However, Hoppe [53] and Rogers [43] report that one SD is about 200 ml, and Hrolfsdottir [54] reports one SD is about 370 ml, so although an exact amount of milk per SD cannot be determined, an estimate between 200 and 350ml seems appropriate.

**Table 4:** Results for all milk–IGF associations, including subgroup analyses

Outcome	N_s_	N_p_	Effect estimate and range (from Albatross plots)
*All*			
IGF-I	28	27,408	0.10 (0.05–0.25)
IGF-II	2	12,148	<0.05 (0.00–0.05)
IGFBP-1	2	1,944	0.00 (NA)
IGFBP-2	2	2,940	(− 0.10 to − 0.05)
IGFBP-3	15	24,744	0.05 (0.00–0.10)
*Caucasian only*			
IGF-I	26	15,852	0.10 (0.05–0.25)
IGFBP-3	14	13,935	0.05 (0.00–0.10)

*N*_*s*_ number of studies within each analysis, *N*_*p*_ number of participants across all studies

Two studies (Martin [27] and Ben-Shlomo [28]) studied the effects of milk intake in childhood and IGF-I and IGFBP3 levels in adulthood, and were considered separately. The Martin study also included IGF-II and IGFBP2, and the results of these were also considered separately.

### The association between milk and IGF-I

**Fig. 3:**
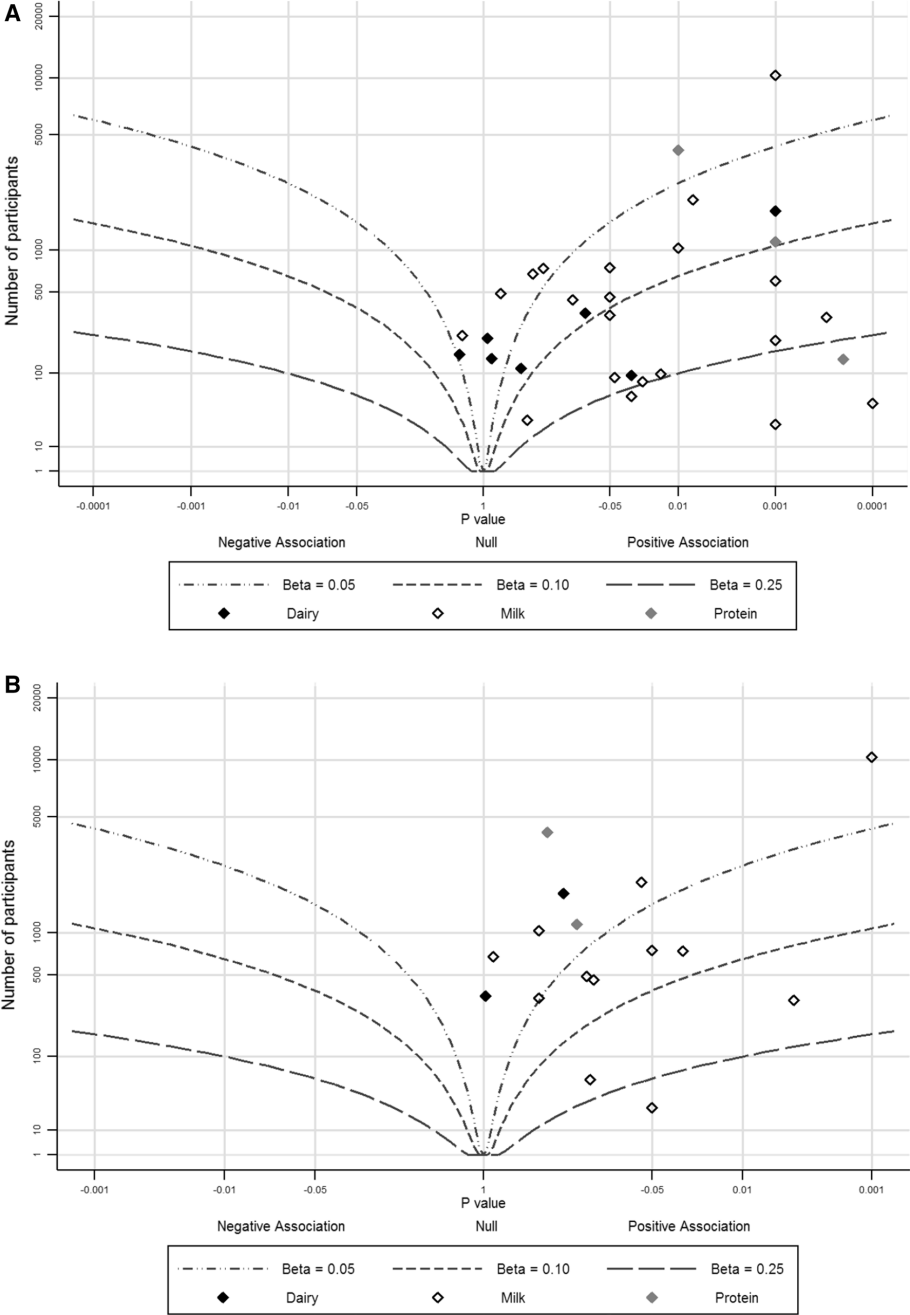
Albatross plots for each outcome: **a** IGF-I and **b** IGFBP-3, stratified by exposure. Each *point* represents a single study included in the meta-analysis, with the effect estimate (represented as a *p* value), plotted against the number of subjects included within each study. Effect estimates are standardized beta coefficients. Where *p* values were presented as <0.05, they were plotted as 0.05 as a conservative estimate

Of the 31 data points (from 28 studies) included in the main IGF-I analysis, 29 data points showed positive associations of milk and dairy intake with IGF-I levels compared to two data points that showed negative or null associations. The estimated standardized effect size was 0.10 SD increase in IGF-I per 1 SD increase in milk (estimated range 0.05–0.25 SDs), from observation of the albatross plot (Fig. 3a). The combined *p* value for a positive association was 2.2 × 10^−27^. All studies with non-Caucasian subjects displayed a positive association between milk intake and PCa risk; in particular, two studies [30, 52] had *p* values of 0.0001 and 0.001, respectively, and both studies had a sample size of less than 100. When considering only Caucasians, the overall impression from the albatross plot did not change; the effect estimate was still considered to be around 0.10 SD.

Of the 31 data points, 18 had an estimated standardized effect size between 0.05 and 0.25 SDs and four had an effect size of more than 0.25 SD. Eleven of these data points (61%) used milk as an exposure, including two that had an estimated standardized effect size of more than 0.25 SD [30, 53].

When considering studies that examined the association between milk and IGF-I, the 31 data points (from 28 studies) showed consistency in their direction of effect, as all but two associations seen were positive.

The largest effect was seen in the Rich-Edwards study [30], where 26 Mongolian children, who had a very low baseline milk intake, were given milk supplements; IGF-I and IGFBP-3 levels were compared before and after the supplementation. At the end of the month, mean IGF-I levels rose from 290.93 ng/ml (SD 93.98 ng/ml) to 358.34 ng/ml (SD 125.62 ng/ml), an increase of 23% (*p* < 0.0001). As no study deprived the participants of milk/dairy, this study is the best estimate of the difference in IGF-I levels between very little and some milk intake, although the small number of children limit our ability to draw firm conclusions.

A large effect was also seen in the Hoppe study [53], where 24 8-year-old children were placed in two equally sized groups; the first group was asked to drink 1.5 l of skimmed milk per day, the second to eat 250 g of low-fat meat per day, with the remaining diet up to choice. Over 7 days, the IGF-I level of the milk-group children rose from 209.3 ng/ml (SD 54.9 ng/ml) to 249.0 ng/ml (SD 66.8 ng/ml), an increase of 19% (*p* < 0.001), while the IGF-I level of the meat-group children did not change. Although both these studies were conducted in small numbers of children, they both show that large increases in milk intake can increase IGF-I levels over a short period of time.

The Cadogan and Zhu RCTs [50, 52] both supplemented the diet of children with milk, with a longer follow-up of between 18 and 24 months, and both showed that milk increases IGF-I over longer periods, albeit with a smaller effect size. The Heaney RCT [51] showed that a 12-week milk supplement in older people (mean 65 years) also increased IGF-I levels. Prospective cohort and retrospective studies in general showed a null or positive effect, consistent with but generally weaker than the RCTs and non-randomized experimental studies. For cross-sectional studies, there appeared to be little difference between studies of children and adults.

The two negative associations seen in the Ben-Shlomo and Martin studies were relatively strong, with *p* values of 0.01 and 0.06, respectively, and estimated effect sizes of between 0.05 and 0.10 SDs [27, 28]. These studies were not included in the main analysis of IGF-I, as the studies had a qualitatively different follow-up period, where the exposure (in childhood) was far removed in time from the outcome (in adulthood). This is a different research question, possibly looking at programming of the IGF-I axis rather than a direct effect of milk intake on IGF levels [196].

In the Ben-Shlomo study, pregnant mothers were randomized during 1972–1974 to either receive vouchers for free milk until the child reached 5 years of age, or to receive no vouchers, with the serum IGF levels measured in offspring at a mean age of 50 years. In the Martin paper, diets and physical health surveys were given to children aged between 0 and 19 years (mean age 7 years) in 1937–1939, and these children were traced and invited to follow-up in 2002–2003, when IGF levels were measured.

### The association between milk and IGFBP-3

In total, 15 studies presented data for IGFBP-3, one of which reported data for two ethnic subgroups. All studies showed a positive association between milk intake and IGFBP-3 levels. The estimated standardized effect size was 0.05SD increase in IGFBP3 per 1 SD increase in milk (estimated range 0.00 to 0.10 SD, Fig. 3b). The combined *p* value was 4.3 × 10^−15^. Similar to IGF-I, the three studies that had the largest effect estimates and smallest P values used milk as an exposure rather than dairy products or protein [36, 45, 53]. There were few non-Caucasian studies looking at IGFBP-3, therefore the estimate for Caucasians only did not change.

Among studies that had examined the effect of milk on IGFBP-3, the 16 data points (from 16 studies) all had a positive effect size, centered on an effect size of about 0.05 SD. Almost all milk-IGFBP-3 studies were prospective cohorts or cross-sectional studies with reasonably similar effect sizes, regardless of age or ethnicity. Hoppe studied both IGF-I and IGFBP-3, and found a positive association (smaller than for IGF-I) with a 5% increase in IGFBP-3 in the milk-group children (*p* < 0.05) [53].

Ben-Shlomo and Martin studied IGFBP-3 as well as IGF-I [27, 28]; as with IGF-I, these studies were not included in the albatross plot due to differences in study design. Both studies found very slightly negative associations (*p* = 0.55, *p* = 0.5, respectively), in contrast to the other studies that all found positive associations.

### The association between milk and other IGF proteins

Two studies examined the association between milk/dairy protein intake and IGF-II levels; a large study of milk intake in elderly Japanese men (*p* = 0.001) [47] and a smaller study of dairy protein intake in Caucasian men (p = 0.28) [48]. The studies showed a very weak positive association between milk and dairy protein intake and IGF-II levels (effect estimate: <0.05 SDs, range 0.00–0.05 SDs, Supplementary Fig. 1A); the combined *p* value was 0.001.

There was no suggestion of an association between milk, dairy product or dairy protein intake and serum levels of IGFBP-1 (effect estimate: 0.00 SD, no range, Supplementary Fig. 1B), as the two included studies had *p* values of 0.39 and 0.83, respectively, and effect estimates in opposite directions [46, 49]. The combined P value was 0.32.

The two studies which examined the association between milk/dairy protein and IGFBP-2 levels both suggested that intake of dairy products and dairy protein led to a small decrease in serum levels of IGFBP-2 (effect estimate between −0.10 and −0.05 SDs Supplementary Fig. 1C); the combined *p* value was 0.00021.

The Martin study [27] also examined IGF-II and IGFBP-2, which both showed a slight negative association with *p* values of 0.5 and 0.3, respectively.

### IGF–Prostate Cancer: human studies

#### Circulatory levels of IGF and Prostate Cancer risk

In total, 59 studies were included in this meta-analysis, consisting of 18 prospective and 41 retrospective studies. Nine studies had not been included in the Rowlands 2012 paper [129, 133, 134, 142, 147, 148, 152–154] and eight studies that were included in the Rowlands 2012 analysis were excluded due to critical RoB [175–178, 180–183]. Data were extracted for all five IGF and IGFBP peptides. All results presented are from random effects meta-analysis, with fixed effect results commented on if necessary.

**Fig. 4:**
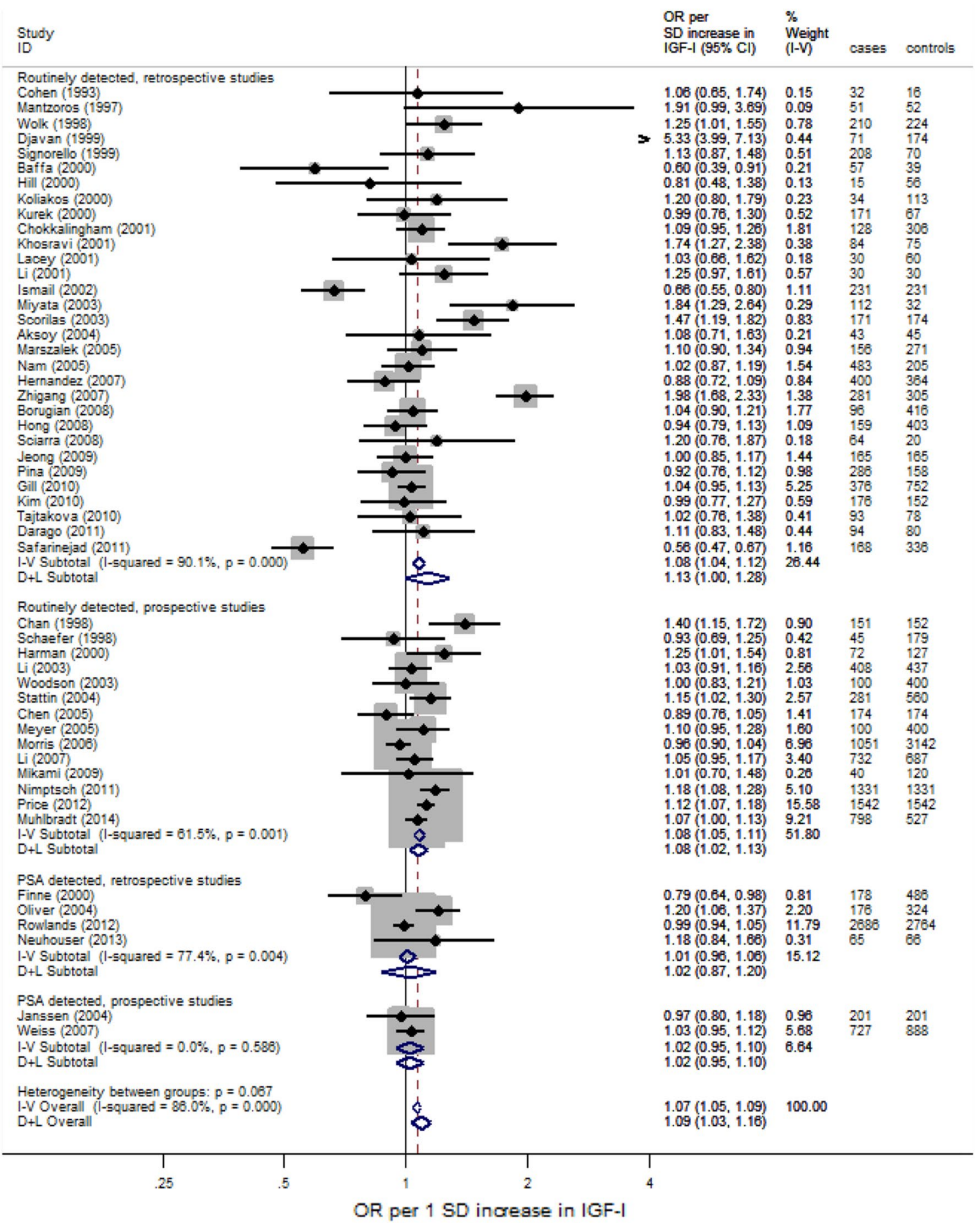
Forest plot for all studies that presented data on circulatory levels of IGF-I in relation to PCa risk, stratified by study design (prospective vs retrospective) and PSA-detected PCa cases

**Fig. 5:**
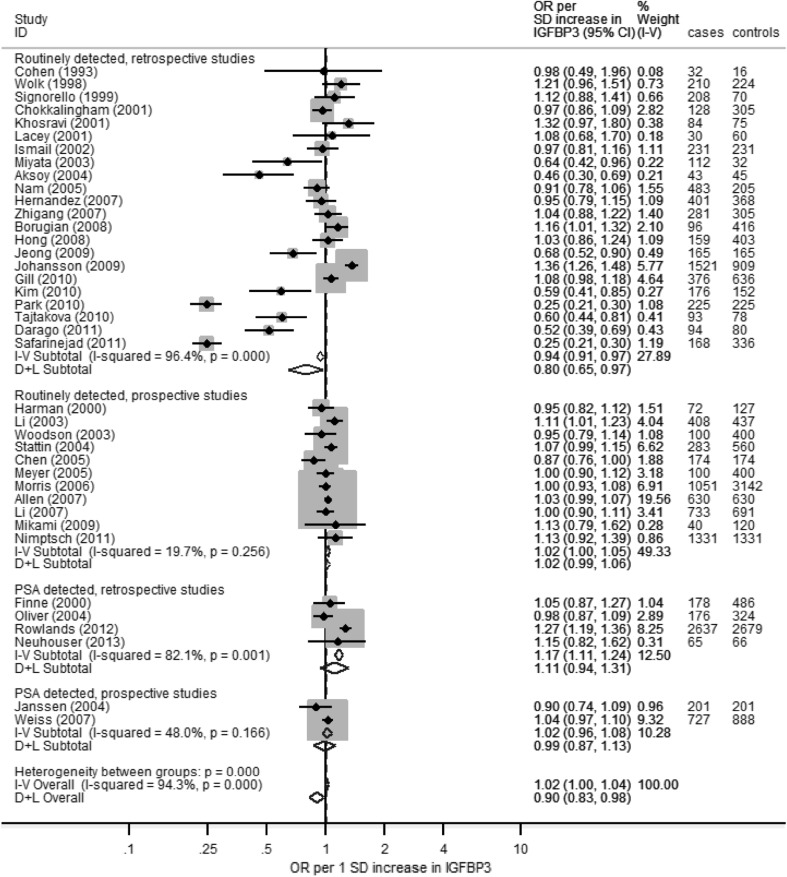
Forest plot for all studies that presented data on circulatory levels of IGFBP-3 in relation to PCa risk, stratified by study design (prospective vs retrospective) and PSA-detected PCa cases

OR values (95% CI) per SD of exposure increase for retrospective and prospective values combined were as follows (Table 5): IGF-I, *n* studies = 51, OR 1.09 (95% CI 1.03, 1.16) (Fig. 4); IGF-II, *n* = 10, OR 1.07 (0.97, 1.18); IGFBP-1, *n* = 4, OR 1.02 (0.77, 1.34); IGFBP-2, *n* = 6, OR 1.07 (0.91, 1.25); and IGFBP-3, *n* = 39, OR 0.90 (0.83, 0.98) (Fig. 5). Overall, there was a moderate-to-high degree of inconsistency across the studies for all five IGF and IGFBP peptides (I^2^ 66–88%; Supplementary Fig. 2) and the inconsistency within the retrospective data was consistently greater compared to prospective studies. Due to extreme outliers, the fixed effects meta-analysis result for IGFBP-3 shows a very small positive association with PCa risk, whereas the random effects result shows a small negative association (fixed OR 1.02 [1.00, 1.04]; random OR 0.90 [0.83, 0.98]).

**Table 5:** Results for all IGF–PCa associations, including subgroup analyses

Exposure	All					Retrospective					Prospective				
	N_s_	N_p_	OR (95% CI)	*p* value	I^2^	N1	N2	OR (95% CI)	*p* value	I^2^	N1	N2	OR (95% CI)	*p* value	I^2^
*All*															
IGF-I	51	35,509	1.09 (1.03, 1.16)	0.003	0.86	35	16,889	1.12 (1.01, 1.23)	0.04	0.89	16	18,620	1.07 (1.02, 1.12)	0.004	0.58
IGF-II	10	12,543	1.07 (0.97, 1.18)	0.18	0.83	7	7,651	1.16 (1.02, 1.32)	0.02	0.81	3	4,892	0.93 (0.81, 1.07)	0.31	0.77
IGFBP-1	4	2,272	1.02 (0.77, 1.34)	0.91	0.88	3	1,830	1.07 (0.72, 1.57)	0.75	0.92	1	442	0.90 (0.72, 1.13)	0.36	–
IGFBP-2	6	6,998	1.07 (0.91, 1.25)	0.39	0.69	4	6,075	1.15 (0.92, 1.43)	0.23	0.76	2	923	0.95 (0.81, 1.12)	0.58	0.00
IGFBP-3	39	32,214	0.90 (0.83, 0.98)	0.02	0.94	26	17,263	0.84 (0.71, 0.99)	0.04	0.96	13	14,951	1.02 (0.99, 1.05)	0.16	0.17
*Advanced*															
IGF-I	12	4,768	1.04 (0.94, 1.14)	0.47	0.69	7	1,556	1.08 (0.91, 1.27)	0.38	0.68	5	3,212	1.00 (0.88, 1.15)	0.95	0.76
IGFBP-3	12	3,618	0.95 (0.87, 1.03)	0.22	0.66	7	1,460	0.86 (0.73, 1.02)	0.08	0.65	5	2,158	1.01 (0.92, 1.11)	0.86	0.67
*Clinically detected*															
IGF-I	45	26,747	1.11 (1.03, 1.18)	0.004	0.87	31	10,144	1.13 (1.00, 1.28)	0.06	0.90	14	16,603	1.08 (1.02, 1.13)	0.004	0.62
IGF-II	7	6,556	1.04 (0.91, 1.19)	0.53	0.85	4	1,664	1.23 (0.93, 1.64)	0.15	0.90	3	4,892	0.93 (0.81, 1.07)	0.31	0.77
IGFBP-1	4	2,272	1.02 (0.77, 1.34)	0.91	0.88	3	1,830	1.07 (0.72, 1.57)	0.75	0.92	1	442	0.90 (0.72, 1.13)	0.36	–
IGFBP-2	3	971	1.18 (0.81, 1.72)	0.4	0.81	1	48	2.10 (1.34, 3.29)	0.001	–	2	923	0.95 (0.81, 1.12)	0.58	0.00
IGFBP-3	33	23,586	0.87 (0.79, 0.97)	0.01	0.95	22	10,652	0.80 (0.65, 0.97)	0.03	0.96	11	12,934	1.02 (0.99, 1.06)	0.21	0.20
*PSA detected*															
IGF-I	6	8,762	1.02 (0.94, 1.11)	0.67	0.63	4	6,745	1.02 (0.87, 1.20)	0.78	0.77	2	2,017	1.02 (0.95, 1.10)	0.56	0.00
IGF-II	3	5,987	1.15 (1.08, 1.22)	4E-06	0.00	3	5,987	1.15 (1.08, 1.22)	4E-06	0.00	0	0	NA	NA	–
IGFBP-1	0	0	NA	NA	–	0	0	NA	NA	-	0	0	NA	NA	–
IGFBP-2	3	6,027	1.05 (0.89, 1.24)	0.54	0.60	3	6,027	1.05 (0.89, 1.24)	0.54	0.60	0	0	NA	NA	–
IGFBP-3	6	8,628	1.06 (0.94, 1.19)	0.34	0.83	4	6,611	1.11 (0.94, 1.31)	0.23	0.82	2	2,017	0.99 (0.87, 1.13)	0.91	0.48

*N*_*s*_ number of studies within each analysis, *N*_*p*_ number of participants across all studies

Correspondingly, there was some evidence of small study effects in IGFBP-3 studies, possibly indicating publication bias; Egger’s test *p* = 0.02, with funnel plots showing that retrospective studies were responsible for a greater degree of heterogeneity and more extreme effects (Supplementary Fig. 3). IGF-I studies also showed retrospective studies to be more inconsistent, but Egger’s test showed no evidence of small study effects, *p* = 0.44. No other exposures showed signs of publication bias or small study effects in either funnel plots or Egger’s tests. There was no association between sample size and OR when performing meta-regression on prospective IGF-I (*p* = 0.68) or IGFBP-3 (*p* = 0.99) studies.

#### Subgroup analyses

##### Advanced prostate cancer risk

Data for advanced PCa risk and IGF-I and IGFBP-3 were presented in 12 studies each. OR values (95% CI) per standard deviation of exposure increase for retrospective and prospective studies combined were as follows: IGF-I, *n* = 12, OR 1.04 (0.94, 1.14) and IGFBP-3, *n* = 12, OR 0.95 (0.87, 1.03) (Supplementary Fig. 4). Egger’s test and funnel plots showed small study effects and publication bias for IGFBP-3 (*p* = 0.006), but not IGF-I (*p* = 0.55). The estimates for advanced PCa risk and all PCa risk were not materially different for either IGF-I or IGFBP-3 (*p* = 0.41 and *p* = 0.37, respectively).

##### Retrospective versus prospective studies

Inconsistency was much greater among retrospective studies compared to prospective studies across all subgroup analyses and overall. OR values were consistently higher in all retrospective analyses for all five IGF or IGFBP peptides, with the exception of IGFBP-3, which was slightly lower (for IGFBP-3: retrospective OR 0.84 [0.71, 0.99]; prospective OR 1.02 (0.99, 1.05); *p* = 0.01 for difference between prospective and retrospective studies) (see Table 5).

##### PSA screened versus clinically detected

In total, there were far fewer studies that used PSA screening to detect cases (*n* = 6) compared to those where cases were identified clinically (*n* = 59). Overall, there was no discernible trend in the effect of PCa detection methods on IGF levels and PCa risk (Table 5). The only distinct difference in OR values between these two subgroups was in IGFBP-3, where the PSA-detected PCa OR was 1.06 (0.94, 1.19), higher than the clinically detected PCa OR of 0.87 (0.79, 0.97), *p* = 0.02.

### Genetic data

In total, 18 studies presented data relevant to genes or SNPs within the IGF pathway, in relation to PCa risk. Of these, data for only two polymorphisms: IGF-I (CA)n repeat and IGFBP-3 SNP-202A/C, were presented in three or more studies, allowing for meta-analyses. Another 46 SNPs or genes were identified in the review; however, only one set of data was available for 42 of the SNPs and two sets of data for four of the SNPs (Supplementary Table 2). Again, all results presented are from random effects meta-analysis.

OR values (95% CI) for the two meta-analysable polymorphisms, were as follows: IGF-I (CA)n (*n* = 5 studies) when compared against a 19/19 repeat, where X is anything other than 19, the X/X repeat had an OR 0.98 (0.56, 1.73), and X/19 repeat had an OR 0.99 (0.63, 1.55). For IGFBP-3-202A/C SNP (*n* = 8 studies), when compared against the A/A allele, the A/C allele had an OR 1.22 (0.84, 1.79), and the C/C allele had an OR 1.51 (1.03, 2.21) (Supplementary Fig. 5).

### IGF–prostate cancer: animal models

Of the 10 animal studies included in the analysis (Table 6), eight provided data on mechanistic IGF pathways, in relation to PCa risk or progression. Four of these studies were transgenic mice models, which over-expressed [186] or knocked-out *IGFBP-3* [189], or knocked-out *IGF-IR* [187] or *IGF-I* [188]. The remaining four studies were xenograft models that used antibodies to bind to IGF peptides [190, 193] or IGF-IR [191] or IGFBP-3 [192].

**Table 6:** Characteristics of IGF–Pca animal studies ordered by study type (transgenic or xenograft models) and year of publication

Author (year)	Model	Experimental	Control	*n* (exp:control)	Outcome	Follow-up	Effects estimate	Overall risk of bias
*Animal studies*								
Silha (2006)	Transgenic mice, where experimental over-express IGFBP3	LPB Tag /PGKBP-3; LPB Tag/CMVBP-3	LPB Tag/WT	231	Tumor weight	21 weeks	*p* < 0.001 tumors smaller in experimental	Unclear
Sutherland (2008)	Transgenic IGFR knockout mice	IGFR KO (Cre/loxP) IGFR titrated (Cre/wt)	IGF-IR intact (loxP/loxP; loxP/WT)	66	Tumor weight	12 weeks	IGFR intact vs IGFR titrated p = 0.02	Unclear
Anzo (2008)	Transgenic TRAMP mice, targeted deletion of Igf1	LID-TRAMP mice, where LID have deleted Igf1 gene and low levels of IGF-I	L/L-TRAMP mice	45 (25;20)	Tumor progression (Lymph node metastasis)	36 weeks	*p* = 0.93 (% mice with metastasis)	Unclear
Mehta (2011)	Transgenic (knockout) mice deficient in IGFBP3	IGFBP-3KO:Myc	WT:Myc	NS	Tumor progression; lung metastasis	80 weeks	*p* < 0.05 (% mice with metastasis), higher for experimental	Unclear
Goya (2004)	Xenograft of human cell line and human adult bone in NOD/SCID mice	Antibody against IGF-I and IGF-II (KMI468)	Control antibody (KMI762)	NS	Bone tumor area	4 weeks	*p* < 0.001 tumors smaller in experimental	Unclear
Wu (2005)	Human cell line-derived xenograft in nude mice	Antibody A12- anti IGF-IR	Saline solution	NS	Tumor volume	5 weeks	*p* < 0.05 tumors smaller in experimental	Unclear
Ingermann (2010)	Human cell line-derived xenograft in nude mice	IGFBP3R binds to IGFBP3	PBS solution	NS	Tumor volume	16 days	Control: 302 ± 54.5 mm^3^ Exp.193 ± 28.6 mm^3^	Unclear
Kimura (2010)	Xenograft of human cell line and human adult bone in NOD/SCID mice	Antibody against IGF2 (M610)	Control antibody (M102.4)	24 (16; 8)	Bone tumor area	4 weeks	Control: 2.2 ± 0.9 mm^2^ Exp.: 0.8 ± 1.2 mm^2^ *p* = 0.01	Unclear
*Supporting evidence*								
DiGiovanni (2000)	Transgenic mice BK5.IGF- mice, over expression of IGF-1	BK5.IGF-1 transgenic mice	Non-transgenic mice	NS	Hallmarks of cancer	Various	Supporting evidence only	Unclear
Fu (2008)	Transgenic	C57/B6 mice	N/A	NS	LOI in IGF-2	NS	Supporting evidence only	Unclear

Seven out of the eight studies found statistically significant differences between experimental and control models, with a small level of consistency between results (Table 6). Two studies found a positive association between tumor progression and IGFBP-3 suppression or deficiency [189, 192], while another found tumor size to be smaller within mice that over-expressed IGFBP-3 [186]. In addition, both studies that provided data on IGF-II found that IGF-II suppressed mice had significantly smaller tumors [190, 193]. Two studies had contradictory results with regards to IGF receptors (IGF-IR), in that deleted IGF-IR accelerated the emergence of aggressive PCa in one experimental model [187], while in another tumors were smaller in IGF-IR suppressed mice [191].

One animal study investigated the association between loss of imprinting in IGF-II and PCa susceptibility [195]. The paper was a short communication and did not provide enough data to be included in the main analysis; however, it was included as supporting evidence. The remaining studies presented only data on the hallmarks of cancer as outcomes and were therefore also included only in supporting evidence [194].

### Supporting evidence

In total, 49 studies were included within the supporting evidence category; 16 of these studies investigated the association between circulating levels of IGF-I (*n* = 14), IGF-II (*n* = 2), IGFBP-2 (*n* = 2) or IGFBP-3 (*n* = 8) with prostate cancer risk or prostate cancer outcomes such as Gleason grade, TNM stage or PSA-progression free survival. 18 studies investigated the association between tissue expression of IGF-I (*n* = 5), IGF-II (*n* = 4), IGF-IR (*n* = 7), IGFBP-2 (*n* = 7), IGFBP-3 (*n* = 6) with risk of prostate cancer or prostate cancer outcomes such as Gleason Grade or TNM stage. Fifteen studies investigated whether genetic or epigenetic variations in the *IGF-I* (*n* = 5), *IGF-II* (*n* = 7), *IGFBP-2* (*n* = 2) or *IGFBP-3* (*n* = 3) gene were associated with prostate cancer risk or outcomes such as Gleason score, TNM stage, PSA recurrence or survival. Results from individual studies were generally not consistent with each other, and therefore did not influence our overall conclusions. The results are presented in Supplementary Tables 3, 4 and 5.

### Risk of bias and GRADE

Generally there was a moderate RoB across the majority of study types, with the exception of animal models where the overall RoB was ‘unclear’ due to the lack of information provided within the papers to inform a number of the RoB sub-categories. Only 11 human observational studies were classed as having critical RoB, and therefore excluded from the meta-analysis on this basis. All but one of the human RCTs in the Milk–IGF analysis were given an ‘unclear’ RoB rating, due to the lack of data to inform particular RoB sub-categories.

The overall GRADE assessments found that there was moderate evidence from human studies that milk intake increased IGF-I levels and there is low-level evidence that milk reduces IGFBP-3 levels. We also found moderate evidence from human studies that increasing IGF-I levels increases prostate cancer and advanced prostate cancer risk, and low-level evidence of no effect of IGFBP-3 levels on prostate cancer and advanced prostate cancer risk. It should be noted that although the evidence for an association between IGF-I and prostate cancer was strong, observational studies cannot by themselves offer strong evidence for causal associations; therefore there is only moderate evidence that IGF-I levels increases prostate cancer risk. For associations between all other IGF biomarkers, there was very low-level evidence of any effect. Common reasons for downgrading were imprecision (especially for IGF biomarkers which had a small number of studies) and publication bias. For full details on GRADE assessments, see Supplementary Tables 6, 7, 8, 9, 10, 11, 12, 13, 14, 15, 16, 17, and 18.

The animal studies were collectively found to offer very low-level evidence that the IGF pathway was related to prostate cancer risk. The reasons for this are as follows: (1) although they were all experimental studies, they had unclear risk of bias due to a lack of information on key variables; (2) we were unable to look at consistency across studies as each study was very different from the other; (3) we downgraded animal studies for indirectness because in the animal studies, components of the IGF pathway were over-expressed to very high levels or knocked out which does not reflect variation within the normal range in humans. In addition, animal studies measured outcomes in terms of tumor weight or volume rather than incidence; the level of imprecision was generally high due to the small number of animals in each experiment and the inability to combine across very different studies. Finally we felt that there was likely to be substantial publication bias. It should be noted, however, that GRADE assessments were designed for use in human studies rather than animal studies.

## Discussion

### Milk–IGF

Based on the overall synthesis of data, there was moderate evidence to suggest that milk intake was positively associated with increased levels of IGF-I and IGFBP-3. This trend appeared to be greater in studies that consisted of non-Caucasian subjects, particularly in IGF-I, and supports results from previous studies that demonstrate that ethnicity can be a contributing factor to increased levels of circulating IGF. It is not possible in this review to elucidate whether ethnicity or differences in normal milk intake have caused these differences. However, although there may be a true difference between ethnicities, it is more likely that lower milk intake in non-Caucasian ethnicities causes larger effects of milk on IGFs, both in experiments where milk is added to the diet and in observational studies where the range of milk intake is much larger.

Across both analyses for IGF-I and IGFBP-3, the largest effect on IGF levels was seen when milk was used as an exposure, rather than dairy protein or its products. Studies in the IGFBP-3 analysis, that used dairy protein or products as exposures, were not seen to contribute to the overall positive association; rather this association was observed only in those studies that used milk as a standalone exposure. Although the original search terms for this analysis were designed to include dairy protein and products as additional exposures, to ensure a broad coverage of exposure types that could include milk, the overall results suggest that ‘diluted’ exposure categories may not be as pertinent in elucidating the true association between milk and IGF levels.

The analysis of IGFBP-2 in this arm of the study consisted of only two studies, both of which classified the exposures as dairy protein and products. Despite the small number of studies and ‘diluted’ exposure types, the analysis produced weak evidence to suggest that IGFBP-2 levels decrease with these types of exposures. Speculatively, further studies using milk alone as an exposure may be useful to ascertain whether there is a stronger negative association between IGFBP-2 and milk intake, compared to the results in this analysis that may be under-powered. Additionally, a similar observation can be made for the weak positive association that was found between IGF-II and milk and dairy protein, as only two studies provided data for this protein.

IGFBP-1 was the only protein to produce a null association with milk and dairy proteins and products; however, there were only two studies available for this analysis, both of which used dairy product or protein as an exposure. Overall, the analyses of the small number of studies available for IGF-II, IGFBP-1, and IGFBP-2 present a more conservative estimate of the effect of milk on levels of these proteins and warrant further data to confirm the presence or absence of any associations.

Overall there was a large amount of data for the milk–IGF analysis, however, there was considerable inconsistency within study design that made it difficult to assimilate the results in such a way to produce a statistic that would represent the associations present across all the studies as a whole. As part of the analysis, we were able to account for ethnicity by differentiating these sets of data within the albatross plots, but other aspects of study design were more difficult to account for. One of the main divisions between study types were those that followed up interventions over weeks or months and those that followed interventions up after a number of years; essentially providing data for short-term and long-term consequences of milk interventions. Studies with a different study design, which looked at milk exposure in childhood and IGF levels in adulthood, were considered separately as these may reflect a programming effect, rather than a direct effect.

One possibility for the apparent shift from a positive association between milk and IGF-I in the short term to a negative association in the long term is programming of the IGF axis in childhood [196]. The theory developed in this study is that high milk consumption in childhood increases IGF-I in the short term; this leads to feedback and programming of the IGF axis, which modulates the amount of IGF-I in the body, leading to a reduced amount of IGF-I in adulthood.

### IGF–Prostate cancer

The results from our IGF–PCa analysis reflect those from the previous meta-analysis [8]; however, the strength of the associations seen in the current paper appears to be smaller; OR values for all IGF proteins in this study were between 0.90 (IGFBP-3) and 1.09 (IGF-I), compared to between 0.88 (IGFBP-3) and 1.21 (IGF-I) in the earlier Rowlands study [7]. However, the directions of effect for all IGF proteins were consistent between this study and Rowlands, and all results had *p* values above 0.05 when testing for differences between estimates [20], there is therefore no evidence to suggest that the results were inconsistent. The standard errors of the current study’s estimates were all lower than in the Rowlands study, indicating greater precision.

These differences in estimates and precision may in part be due to more rigorous risk of bias protocols, which have ensured that the quality of the data presented here is high and that confounding factors (with particular focus on age) have been accounted for. Eight papers, consisting of 941 PCa cases, were included in the Rowlands analysis and were excluded in this study due to age not being considered in the design or analysis of the studies. This may account for some of the differences between the results between the current versus the earlier Rowlands study [8]. The addition of studies published after 2012 may have also affected the results. Additionally, an evaluation of meta-analyses of biomarker associations with cancer risk [197] concluded that there may be bias present specifically in the meta-analyses of IGF with cancer risk, of which the Rowlands meta-analysis was one; by removing studies with the highest risk of bias, it is possible some bias has been removed. This study thus provides a refined and updated assessment of the previous IGF–PCa association.

Both the current study and the Rowlands study, showed a marked difference between the quality of retrospective and prospective studies, both in terms of heterogeneity and possible publication bias; retrospective studies in general had a larger amount of inconsistency and displayed more non-symmetry in funnel plots. All eight studies excluded from this study, but included in Rowlands, were retrospective; this may have contributed to the relative imprecision of the Rowlands results, as well as making those results more extreme.

In our current study, there were few differences between the results from studies with clinically detected vs PSA-detected PCa (Table 5); while there may simply be few differences between IGF and PSA or clinically detected PCa, it is more likely that the lack of difference is due to the difficulty in ascertaining the method by which the PCa cases are found and recruited, and may reflect the ubiquity of the screening for PCa using the PSA in the general population. As PSA testing has become the normal method of testing for prostate cancer in the USA and many other countries worldwide, prostate cancer cases are being found earlier and with more indolent disease. Thus, newer studies likely have a different case-mix to earlier studies, with a greater proportion of early and indolent cancers. If IGF action on prostate cancer affects progression as well as (or as opposed to) incidence, then the overall effect estimate of IGF on prostate cancer will be reduced by a change in case-mix favoring indolent cancers. It is possible that some of the differences between this study and the Rowlands study were as a result of changing the case-mix by excluding older studies, which may have had more clinical cases, (with critical risk of bias) and including newer studies, which may have had more PSA-detected cases. However, this is balanced by our finding showing that advanced prostate cancer risk was no more associated with IGF-I and IGFBP-3 than prostate cancer risk as a whole; if IGF was associated with progression, it would likely be seen when examining advanced prostate cancer risk. However, advanced prostate cancer was defined differently between studies and may not be representative of cancers that have “progressed,” especially as the advanced cancers may have been found earlier through PSA testing.

Within the genetic portion of the IGF–PCa analysis, the C/C genotype of the IGFBP-3 SNP-202A/C, presented a strong, positive association with PCa risk (OR 1.51), alongside a moderate association with the A/C genotype of the same SNP (OR 1.22). This genotype is associated with a higher IGF-I level relative to the A/A genotype [198], and therefore the finding supports the hypothesis that IGF-I is positively associated with PCa risk. Repeats of (CA) did not provide evidence for any association between IGF-I and PCa risk, but only 5 studies were included, which had very inconsistent results; Schildkraut [170] estimated an odds ratio of 3.33 (1.26–8.82) for 19/19 repeats against non-19/non-19 repeats, whereas Tsuchiya [165] estimated an odds ratio of 0.30 (0.12–0.77). This may be due to differences in study populations: Schildkraut recruited men in the USA, with a prevalence of 27% for the 19/19 allele; Tsuchiya recruited men in Japan, with a prevalence of only 6% for the 19/19 allele.

Although a larger number of studies provided data that was eligible for the genetic analysis, only two alleles had enough data to be meta-analyzed. Overall, there is a distinct lack of data with regards to many other genes and SNPs within the IGF pathway, with the majority of SNPs only being studied within one population within one study.

A recent Mendelian randomization study of the IGF pathway and prostate cancer [199] concluded that the IGF pathway may be associated with prostate cancer, but because of the potential for pleiotropy, no individual IGF protein could be identified as having a specific association.

When we subjected the evidence to GRADE assessments, we found that at best there was moderate evidence of an association for milk–IGF-I and milk–IGFBP-3, but for the most part, the evidence was of a low or very low level. GRADE takes into account the totality of evidence to determine the overall strength of a reported association and the potential for it to have been influenced by bias. As most of the evidence on both milk and the IGF pathway, and the IGF pathway and prostate cancer was observational, there is a great deal of potential for bias to have occurred. In addition for some biomarkers (IGF-II, IGFBP-1 and IGFBP-2), the small number of studies carried out meant that the level of imprecision was high. These results indicate that even for the IGF-I–prostate cancer association where many studies have previously been focused, the evidence that IGF-I increases prostate cancer risk is not strong, and there is a need for further high-quality studies that are free from bias to address this question. To our knowledge, GRADE has not previously been applied to animal studies in this way, the main reasons why these studies scored so low in this assessment was the lack of information on experimental methods, the lack of similar studies to assess consistency and the potential for publication bias. As noted, however, GRADE was developed for use in human studies and may not be appropriate when considering animal studies, which have very different methodologies. Additionally, while animal studies may not be suitable for assessing the strength of the evidence underlying mechanistic pathways, they may be extremely useful in identifying and highlighting mechanisms which can then be tested in human studies.

### Combining results from the milk–IGF and IGF–prostate cancer analysis

When looking at the overall results from the milk–IGF and IGF–PCa data, IGF-I and IGFBP-3 are possible mediators of the association between milk intake and PCa. Broadly speaking, the data from this study lend support to the hypothesis that increased milk intake may increase circulating levels of IGF-I, which in turn may increase the risk of PCa.

There is also evidence to suggest that levels of circulating IGBP-3 can be increased by milk intake; however, this binding protein appears to have a protective effect against PCa risk (Fig. 5). This finding was only seen in retrospective studies; as such, it should be interpreted with appropriate caution as prospective studies are generally more robust.

These associations are mediated by key factors within both arms of the study, most importantly, length of follow-up across both arms, baseline intake of milk and dairy and ethnicity within the milk–IGF studies and age within the IGF–PCa studies. Within this meta-analysis, the methodology was designed to account for these factors to ensure that they did not bias the overall result. However, combining the effects from differing populations may mask stronger or weaker associations among these populations as individual entities, and therefore the results should be carefully interpreted in this respect.

Results for IGF-II, IGFBP-1, and IGFBP-2 across both arms of the study do not suggest a complete pathway to link milk intake to PCa risk via these IGFs; this is due largely to the paucity of studies examining these biomarkers, future studies may contribute to our understanding.

It should be noted that although the results support short-term milk intake being associated with IGF-I increase, long-term associations may be complicated by programming of the IGF axis. Therefore, drinking milk in childhood may not be associated with prostate cancer risk in the future, whereas drinking milk in adulthood may be; this may have a biological basis, as milk is a food evolved for neonates rather than adults.

### Supporting evidence and animal studies

Animal models can provide evidence to support mechanistic pathways that connect IGF as an intermediate phenotype between milk intake and PCa risk; however, the application of these within a human-based review requires caution. The data from animal studies and results from supporting evidence studies were highly heterogeneous, encompassing a large variety of study designs, outcomes and exposures relating to IGF and PCa risk; as such, it was not possible to carry out detailed meta-analyses for these data. When IGF-I levels were reduced by producing antibodies against IGF-I [190], tumor volume was reduced relative to controls, whereas tumor volume was higher in IGFBP-3 knockout animals [189]. These studies therefore support the associations that were found within the human IGF–PCa analysis.

When assimilating the data from all the supporting evidence studies, the results were conflicting. The studies had looked at the effect of cancer treatment on IGF levels, how IGF levels changed over time in prostate cancer patients, the association between IGF levels and precancerous lesions, the expression of IGF in prostate cancer tissue vs normal prostate and other tissue, haplotypes in IGF-I and prostate cancer risk, SNPs in the IGF pathway and cancer survival, loss of *IGF-II* imprinting and cancer risk, and methylation of IGF genes. These data provided evidences for positive, negative, and null associations between IGF exposures and PCa risk, with positive associations as the most prevalent associations.

### Limitations

An important limitation of this work is that the studies examining the association between milk and IGF did not include sufficient data to perform a meta-analysis, and thus a combined effect estimate could not be calculated. However, the albatross plots allowed an approximate examination of the underlying magnitude of effect in terms of standardised mean differences, allowing conclusions to be drawn about the likely size of the association between milk and different IGFs. We acknowledge that the literature search was completed in March 2014, and therefore more recent relevant studies may have been missed. However, one particular strength of this study is the inclusion of many different data types, and the application of the Risk of Bias and GRADE tools to the data allowing for a more robust conclusion.

## Conclusion

The diversity of studies created a complex meta-analysis structure, which has required strict inclusion/exclusion criteria to allow meta-analysable data across a number of study types. Overall, the combined evidence from human and animal observational, experimental, and genetic studies provides evidence to support a role of the IGF pathway, in particular IGF-I in explaining the association between milk and prostate cancer.

## Electronic supplementary material

Below is the link to the electronic supplementary material.

Supplementary material 1 (DOCX 8208 KB)

Supplementary material 2 (DOCX 145 KB)
