# Supplementary material for: Does milk intake promote prostate cancer initiation or progression via effects on insulin-like growth factors (IGFs)? A systematic review and meta-analysis
Source: Cancer Causes Control. 2017 Mar 30;28(6):497–528. doi: 10.1007/s10552-017-0883-1 (PMC5400803; doi:10.1007/s10552-017-0883-1)
Supplement: Supplementary file 1 — Supplementary material 1 (DOCX 8208 KB) [file 10552_2017_883_MOESM1_ESM.docx]

**Supplementary Figures**


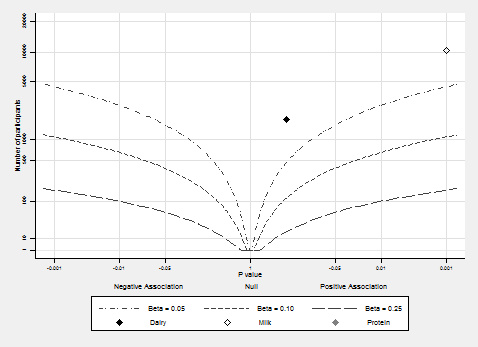

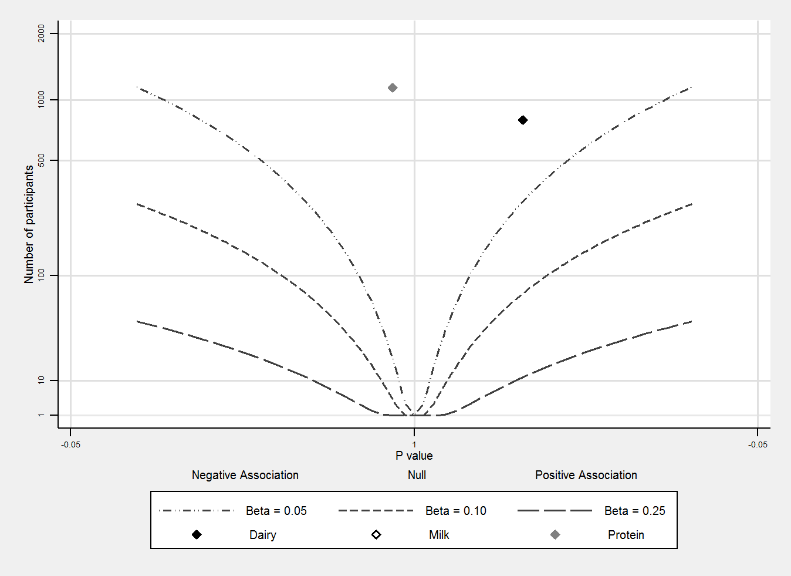

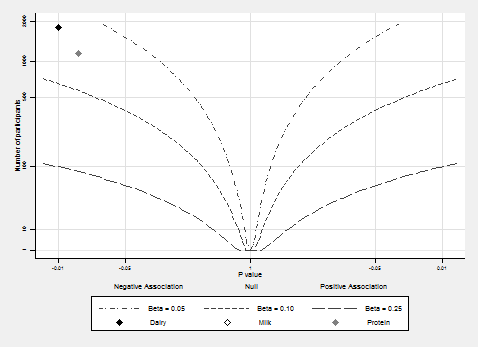


**B**

**A**

**C**

Supplementary Figure 1. Albatross plots for each outcome: A) IGF-II; B); IGFBP-1 and C) IGFBP-2, stratified by exposure. Each point represents a single study included in the meta-analysis, with the effect estimate (represented as a P value), plotted against the number of subjects included within each study. Effect estimates are standardised beta coefficients. Where P values were presented as <0.05, they were plotted as 0.05 as a conservative estimate.


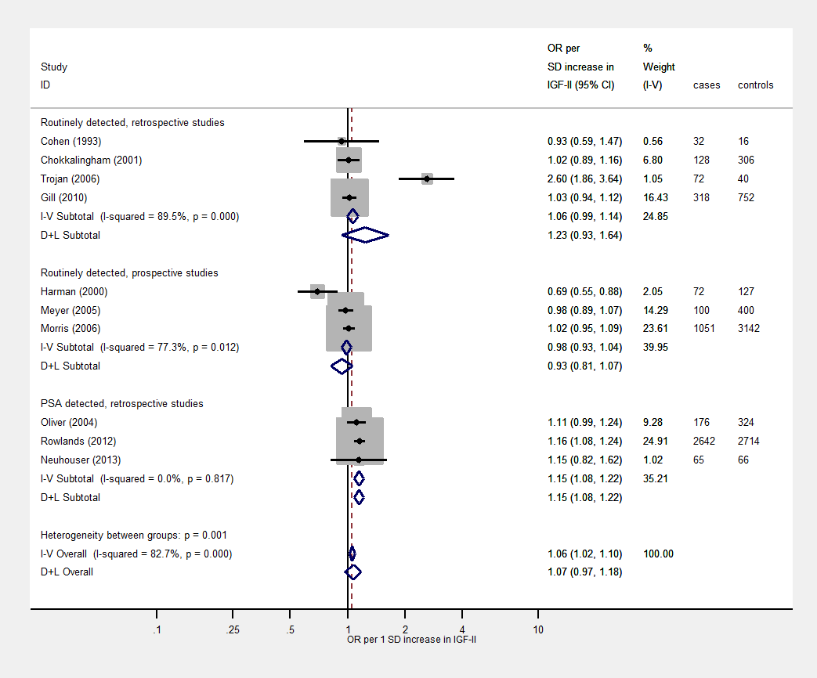


**B**

**A**


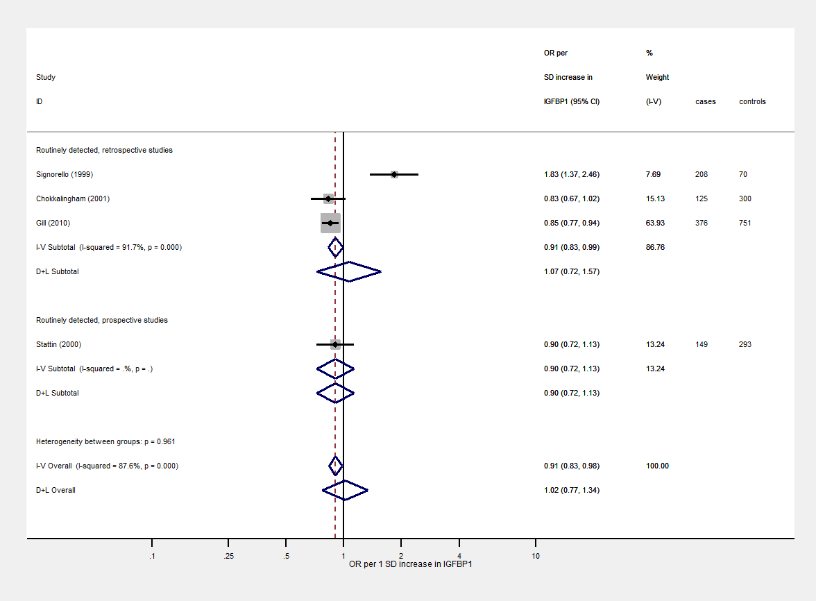


**C**


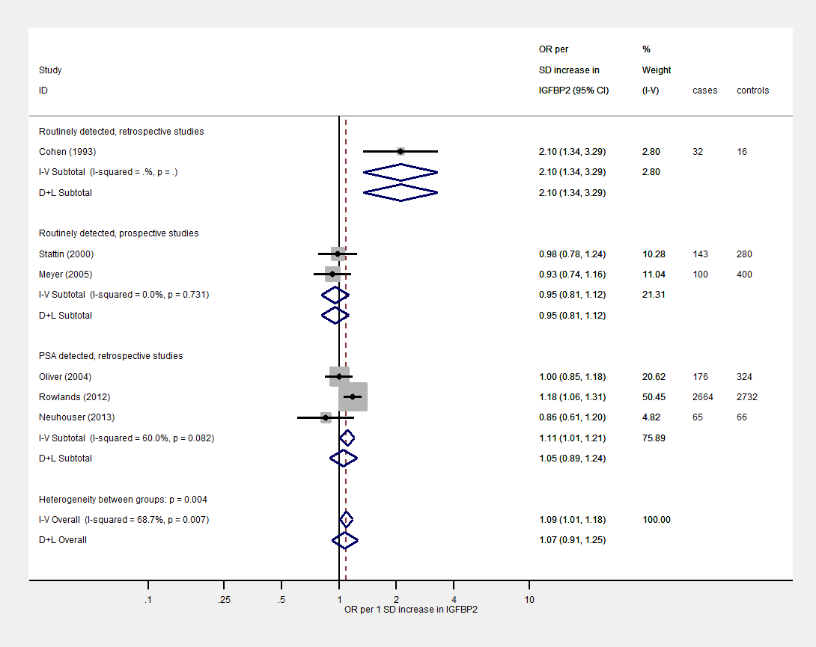


Supplementary Figure 2. Forest plots for all studies that presented data on circulatory levels of A) IGF-II; B) IGFBP-1; and C) IGFBP-2 in relation to PCa risk, stratified by study design (prospective vs retrospective) and PSA detected PCa cases.


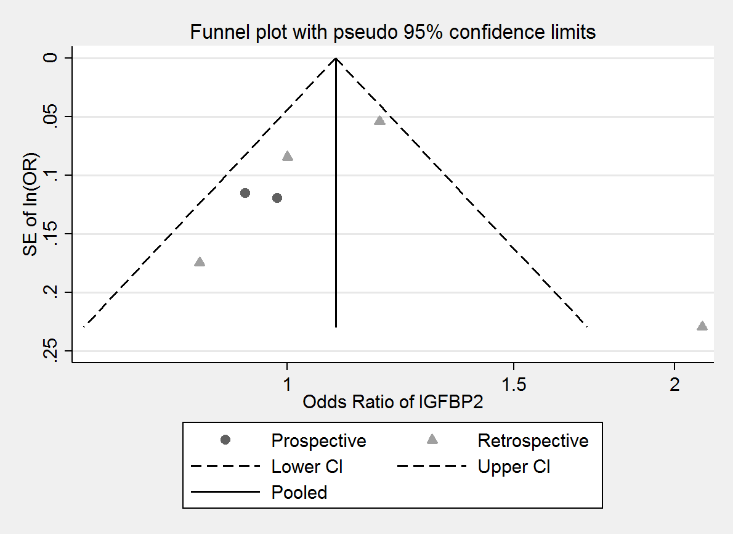

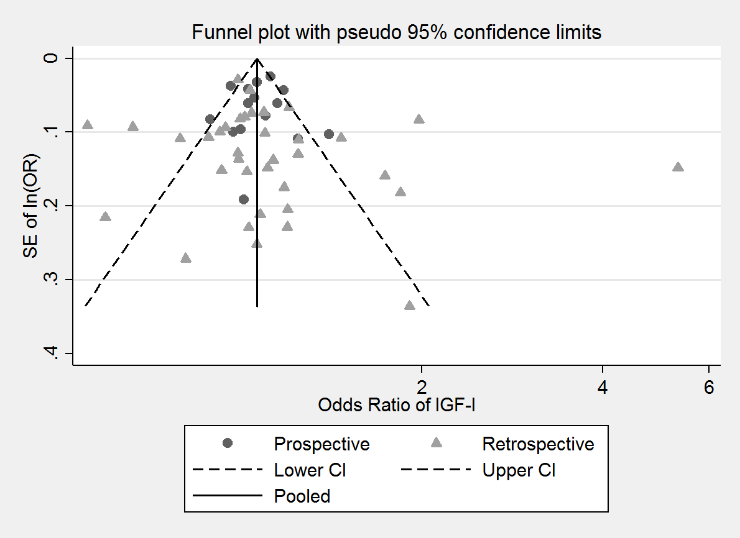


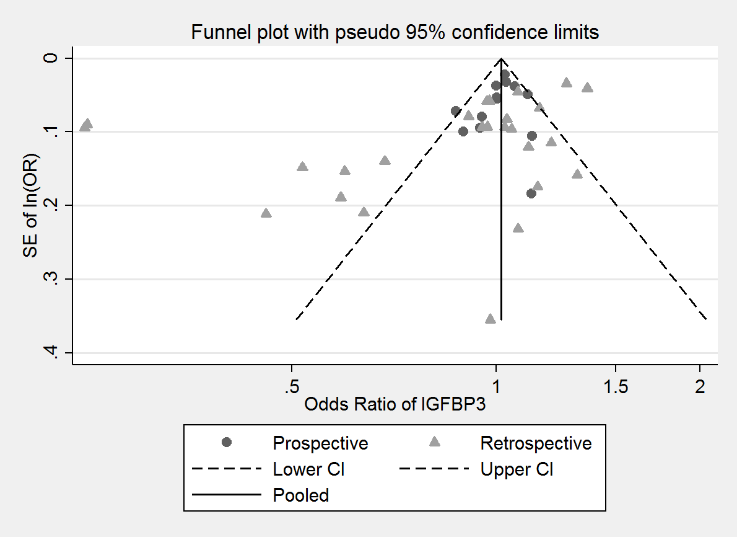


**A**

**B**

**C**

**D**

**E**


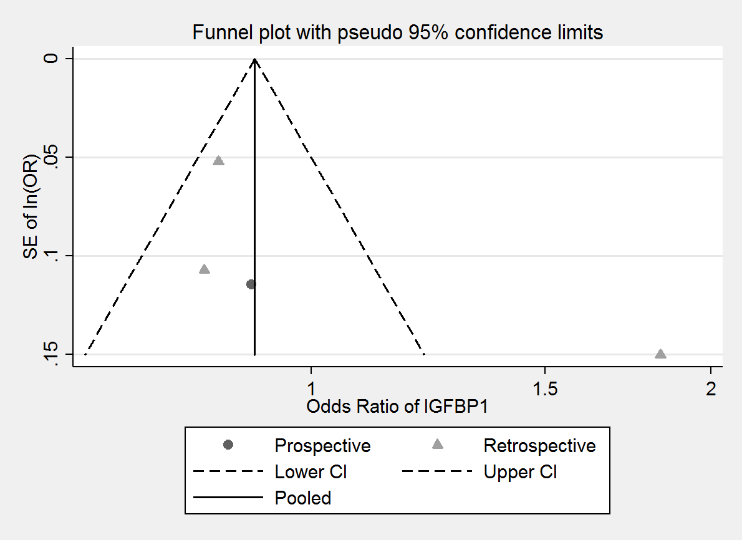


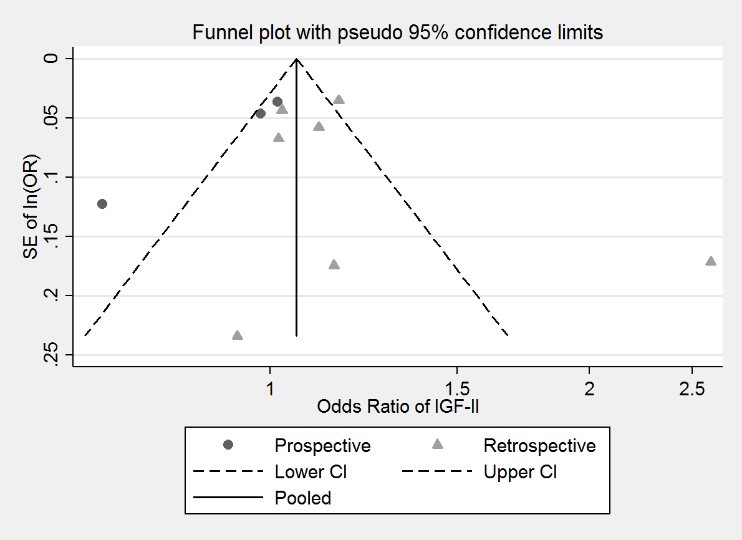


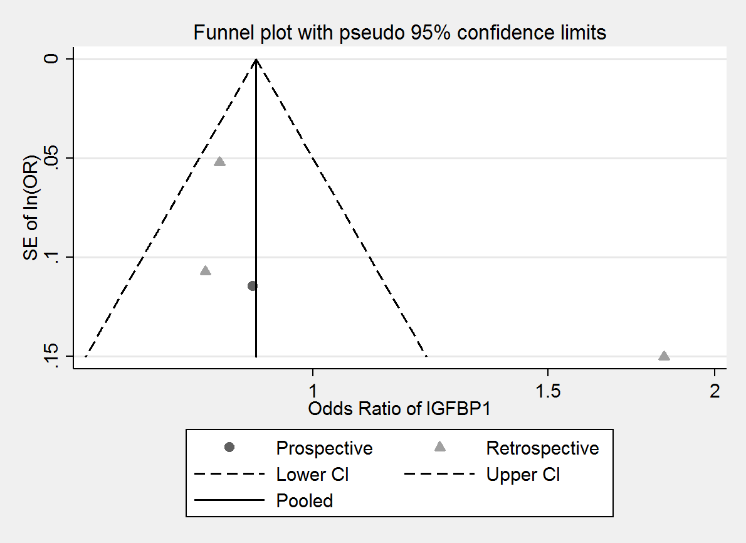


Supplementary Figure 3. Funnel plots for A) IGF-I; B) IGF-II; C) IGFBP-1; D) IGFBP-2; and E) IGFBP-3 against prostate cancer risk, stratified by retrospective and prospective study design.

**
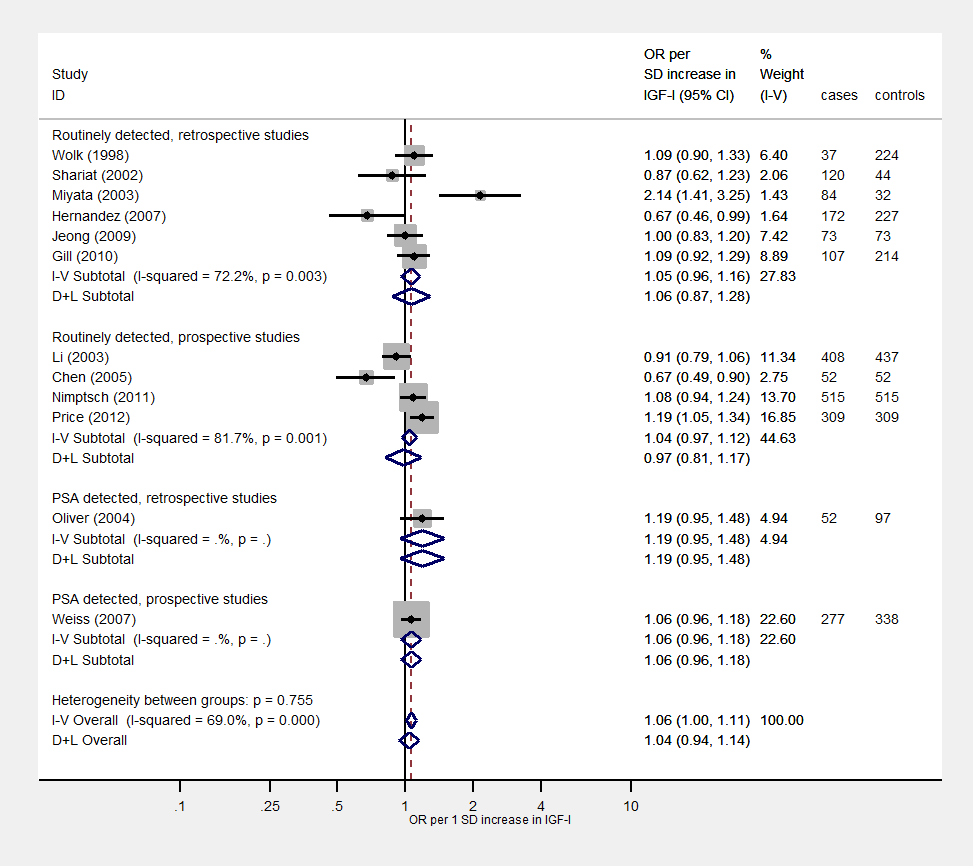
**

**B**

**A**

**
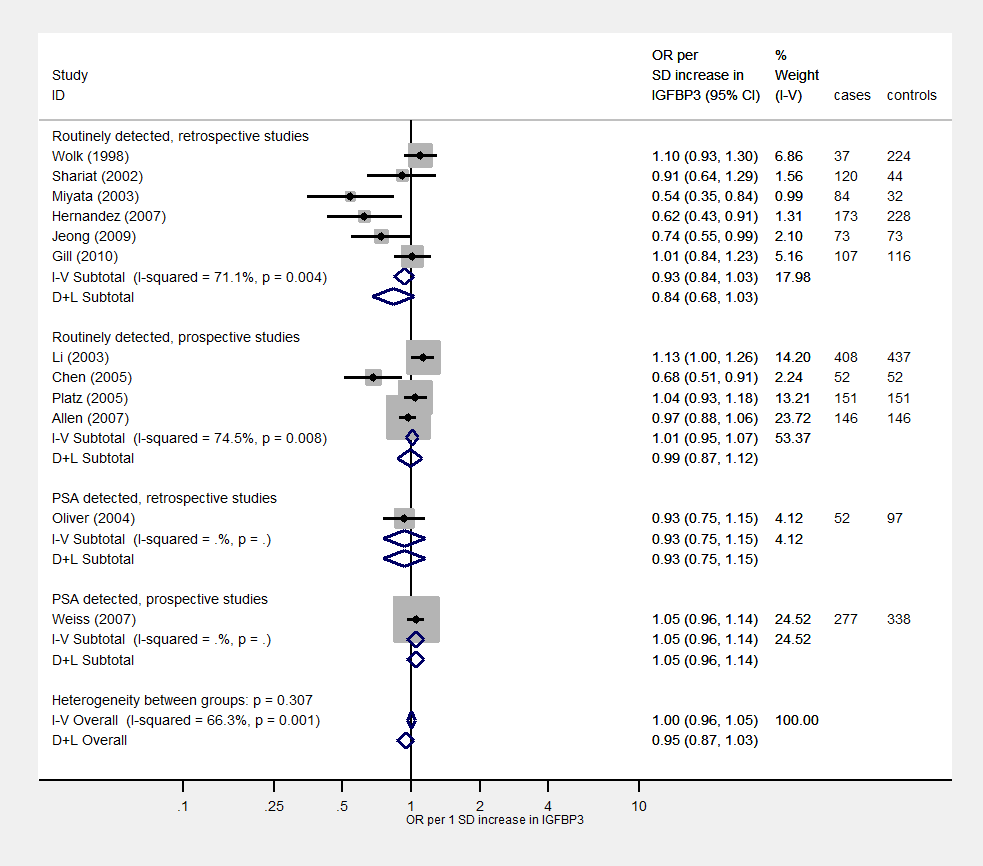
**

Supplementary Figure 4. Forest plot for studies that presented data on circulatory levels of A) IGF-I and B) IGFBP-3 in relation to advanced PCa risk, stratified by study design (prospective vs retrospective) and PSA detected PCa cases.

*
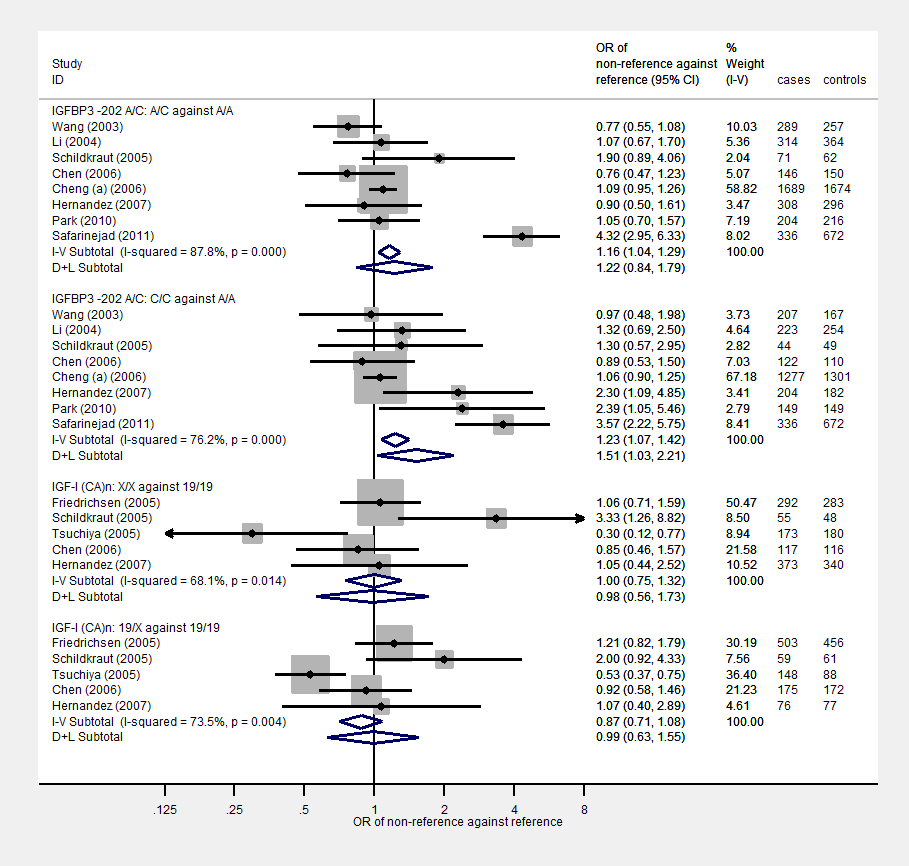
*

Supplementary Figure 5. Forest plot for studies that presented genetic data in relation to PCa risk using A) IGF-I (CA)n repeat and B) IGFBP-3 SNP -202A/C polymorphisms, stratified by alleles.
