# Supplementary material for: Does milk intake promote prostate cancer initiation or progression via effects on insulin-like growth factors (IGFs)? A systematic review and meta-analysis
Source: Cancer Causes Control. 2017 Mar 30;28(6):497–528. doi: 10.1007/s10552-017-0883-1 (PMC5400803; doi:10.1007/s10552-017-0883-1)
Supplement: Supplementary file 2 — Supplementary material 2 (DOCX 145 KB) [file 10552_2017_883_MOESM2_ESM.docx]

| Supplementary Table 1. Characteristics of all human IGF-PCa studies used for circulatory and genetic meta-analyses, stratified by study design and ordered by year of publication. | | | | | | | | | | | | | | | | | | | | | |
| --- | --- | --- | --- | --- | --- | --- | --- | --- | --- | --- | --- | --- | --- | --- | --- | --- | --- | --- | --- | --- | --- |
| **Author (year)** | **Study Name** | **Study Location** | **Assay** | **Control  Type** | **Control Source** | **Mean age at diagnosis (years)** | **Average age of cases (years)** | **Average age of controls (years)** | **Median time between (years)** | **Ethnicity** | **IGF-I** | **IGF-II** | **IGFBP-1** | **IGFBP-2** | **IGFBP-3** | **Genetic** | **Advanced PCa risk** | **PSA Screened** | **Data used** | **Data adjusted?** | **Overall RoB** |
| ***Prospective studies*** | |  |  |  |  |  |  |  |  |  |  |  |  |  |  |  |  |  |  |  |  |
| Chan (1998) | Physicians' Health Study | USA | ELISA | Mixed | PBC |  | M 5yr | 60.3 | 7 | Not Stated | √ | √ |  |  | √ |  |  |  | Quantiles^1^ | √ | Moderate |
| Harman (2000) | Baltimore Longitudinal Study of Aging | USA | RIA | Healthy | PBC | 74 | 64.8 | 65.7 | 12.8 | Multi-ethnic | √ | √ |  |  | √ |  |  |  | Quantiles | √ | Moderate |
| Stattin (2000) | NSHDS | Sweden | IRMA | Mixed | PBC | 63 | 59.7 | 59.6 | 3.3 | Caucasian | √ |  | √ | √ | √ |  |  |  | Quantiles | √ | Moderate |
| Stattin (2001) | NSHDS | Sweden | IRMA | Mixed | PBC |  | 58.4 | 59.5 | 3.9 | Caucasian | √ |  |  |  | √ |  |  |  | None: RCD | √ | Moderate |
| Chan (2002) | Physicians' Health Study | USA | ELISA | Healthy | PBC |  | M 1yr | M 1yr | 9 | Not Stated | √ |  |  |  | √ |  |  |  | None: RCD | √ | Moderate |
| Li (2003) |  | USA | ELISA | Mixed | S |  | 61.3 | 62.8 | 2 | Multi-ethnic | √ |  |  |  | √ |  | √ |  | Quantiles | √ | Moderate |
| Woodson (2003) | ATBC Trial | Finland | ELISA | Healthy | TC | 68.6 | 59 | 56.4 | 9.6 | Not Stated | √ |  |  |  | √ |  |  |  | Quantiles | √ | Moderate |
| Janssen (2004) | ERSPC | Netherlands | IRMA | Healthy | TC | 66.4 | 62.3 | 62.3 | 4 | Not Stated | √ |  |  |  | √ |  |  | √ | None: RCD | √ | Moderate |
| Stattin (2004) | NSHDS | Sweden | IRMA | Mixed | PBC | 63.6 | 59.9 | 59.9 | 3.7 | Caucasian | √ |  |  |  | √ |  |  |  | Quantiles | √ | Moderate |
| Chen (2005) | Cardiovascular Health Study | USA | IRMA | Mixed | PBC |  | 72.3 | 72.3 | 3.4 | Multi-ethnic | √ |  |  |  | √ |  | √ |  | Quantiles | √ | Moderate |
| Meyer (2005) | SUVIMAX Trial | France | CLA | Healthy | TC |  | 55.2 | 55.2 | 5+ | Not Stated | √ | √ |  | √ | √ |  |  |  | Quantiles | √ | Moderate |
| Platz (2005) | Health Professionals Follow Up Study | USA | ELISA | Healthy | PBC | 68.6 | 68.6 | M 1yr | 2.2 | Not Stated | √ |  |  |  | √ |  | √ |  | Quantiles | √ | Moderate |
| Morris (2006) | BUPA study | UK | ELISA | Healthy | PBC |  | 52.4 | 52.4 | 9.7 | Not Stated | √ | √ |  |  | √ |  |  |  | Means |  | Moderate |
| Severi (2006) | Melbourne collaborative cohort | Australia | ELISA | Mixed | PBC | 67 |  |  | 3+ | Multi-ethnic | √ |  |  |  | √ |  |  |  | Excluded |  | Critical |
| Allen (2007) | EPIC | Europe | ELISA | Mixed | PBC | 65 |  |  | 3.4 | Multi-ethnic | √ |  |  |  | √ |  | √ |  | Quantiles | √ | Moderate |
| Li (2007) | Physicians' Health Study | USA | ELISA | Mixed | PBC | 69.4 | 58.9 | 59 | 11 | Caucasian | √ |  |  |  | √ |  |  |  | Means | √ | Moderate |
| Weiss (2007) | PLCO screening trial | USA | ELISA | Healthy | PBC |  | >55 | >55 | 1+ | White | √ |  |  |  | √ |  | √ | √ | Quantiles | √ | Moderate |
| Mikami (2009) | Japan Collaborative Cohort Study | Japan | IRMA | Mixed | PBC |  | 69.3 | 69.1 | 5.3 | Asian | √ |  |  |  | √ |  |  |  | Means | √ | Moderate |
| Gu (2010) | BPC3 |  | ELISA | Mixed | PBC |  | 62.3 | 63.2 | 4.5 | Caucasian | √ |  |  |  | √ |  |  |  | None: RCD | √ | Moderate |
| Nimptsch (2011) | Health professionals follow up study | USA | ELISA | Mixed | PBC | 65 | M 1yr | M 1yr | 6 | Multi-ethnic | √ |  |  |  | √ |  | √ |  | Continuous | √ | Moderate |
| Price (2012) | EPIC | Europe | ELISA | Mixed | PBC |  | 60.12 | 60.1 | 3.4 | Not Stated | √ |  |  |  |  |  | √ |  | Quantiles | √ | Moderate |
| Tsilidis (2012) | BPC3 |  | ELISA | Mixed | PBC |  | 62.9 | 60.2 | 4.9 | Caucasian | √ |  |  |  | √ |  |  |  | None: RCD | √ | Moderate |
| Tsilidis (2013) | BPC3 |  | ELISA | Mixed | PBC |  | 66.2 | 65.9 | 2.1 | Caucasian | √ |  |  |  | √ | √ |  |  | None: RCD | √ | Moderate |
| Muhlbradt (2014) | Physicians' Health Study | USA | ELISA | Mixed | PBC | 70 | 62 | | 8 | Caucasian | √ |  |  |  |  |  |  |  | Quantiles^2^ | √ | Moderate |
| ***Retrospective studies*** | |  |  |  |  |  |  |  |  |  |  |  |  |  |  |  |  |  |  |  |  |
| Cohen (1993) |  | USA | RIA | Healthy | P | Not stated | |  |  | Not Stated | √ | √ |  | √ | √ |  |  |  | Means | √ | Moderate |
| Kanety (1993) |  | Israel | RIA | Healthy | NS |  | 65.8 | 56.4 | 23m | Not Stated |  |  |  | √ | √ |  |  |  | Excluded |  | Critical |
| Ho (1997) |  | Australia | RIA | Mixed | H |  | 74.4 | 68.6 |  | Not Stated | √ | √ |  | √ | √ |  |  |  | Excluded |  | Critical |
| Mantzoros (1997) |  | Athens, Greece | RIA | Healthy | P |  | 71.9 | 71.8 |  | Not Stated | √ |  |  |  |  |  |  |  | Continuous | √ | Moderate |
| Wolk (1998) |  | Sweden | IRMA | Healthy | P |  | 70 | M 10yrs |  | Not Stated | √ |  |  |  | √ |  | √ |  | Quantiles, Continuous | √ | Serious |
| Schaefer (1998) |  | USA | RIA | Healthy | P | 71 |  |  | 1+ | Not Stated | √ |  |  |  |  |  |  |  | Rowlands data |  | Unclear |
| Cutting (1999) |  | UK | IRMA | Healthy | H |  | 73.2 | 67.4 |  | Not Stated | √ |  |  |  |  |  |  |  | Excluded |  | Critical |
| Djavan (1999) |  | Austria | IRMA | Mixed | H |  | 65.7 | 67.7 |  | White | √ |  |  |  |  |  |  |  | Means |  | Serious |
| Signorello (1999) |  | Sweden | IRMA | Mixed | P |  | 69.9 | 70.9 |  | Not Stated | √ |  | √ |  | √ |  |  |  | Means | √ | Moderate |
| Hill (2000) |  | Czech Republic | IRMA | BPH | H |  | 77.1 | 72.2 |  | Not Stated | √ |  |  |  |  |  |  |  | Means |  | Serious |
| Koliakos (2000) |  | Greece | IRMA | BPH | H |  | 67 | 69 |  | Not Stated | √ |  |  |  |  |  |  |  | Means |  | Serious |
| Baffa (2000) |  | USA | ELISA | Healthy | NS | Not stated | |  |  | Not Stated | √ |  |  |  |  |  |  |  | Means | √ | Moderate |
| Finne (2000) | Finnish Prostate Cancer Screening Trial | Finland | ELISA | Mixed | PBC |  | 62 | 62.6 | 2-18m | Not Stated | √ |  |  |  | √ |  |  | √ | Continuous | √ | Moderate |
| Kurek (2000) |  | Germany | CLA | Healthy | NS |  | 66.2 | 64.5 |  | Not Stated | √ |  |  |  |  |  |  |  | Means | √ | Moderate |
| Lacey (2001) |  | USA | ELISA | Healthy | PBC | 70.6 |  |  |  | White | √ |  |  |  | √ |  |  |  | Means | √ | Moderate |
| Chokkalingham (2001) |  | China | ELISA | Mixed | P |  | 71.9 | 72 |  | Asian | √ | √ | √ |  | √ |  |  |  | Quantiles | √ | Moderate |
| Khosravi (2001) |  | Canada | ELISA | BPH | NS |  | 64.8 | 65.6 |  | Not Stated | √ |  |  |  | √ |  |  |  | Means |  | Serious |
| Li (2001) |  | China | RIA | Mixed | H |  | 74.7 | 74.8 |  | Asian | √ |  |  |  |  |  |  |  | Quantiles |  | Moderate |
| Perk (2001) |  | Turkey | IRMA | Healthy | H |  | 62.3 | 36 |  | Not Stated | √ |  |  |  |  |  |  |  | Excluded |  | Critical |
| Ismail (2002) |  | Montreal, Canada | ELISA | Mixed | H |  | 64.6 | 64.5 |  | Not stated | √ |  |  |  | √ |  |  |  | Means | √ | Serious |
| Shariat (2002) |  | USA | ELISA | Healthy | H | 63 | 29 | 44 |  | Not Stated | √ |  |  | √ | √ |  |  |  | Rowlands data |  | Unclear |
| Peng** (2002) |  | China | IRMA | Mixed |  |  | 74.8 | 64.8 |  | Not Stated | √ |  |  |  |  |  |  |  | Excluded |  | Critical |
| Kaaks (2003) | NSHDS | Sweden | RIA | Mixed | PBC |  |  |  | 1m-10yrs | Caucasian | √ |  |  |  |  |  |  |  | None: RCD |  | Moderate |
| Miyata (2003) |  | Nagasaki University, Japan | IRMA | BPH | H |  | 70.9 | 69.8 |  | Asian | √ |  |  |  | √ |  | √ |  | Means |  | Serious |
| Scorilas (2003) | Padova | Italy | ELISA | BPH | H |  | 68 | 65 |  | Not Stated | √ |  |  |  |  |  |  |  | Means |  | Serious |
| Aksoy (2004) |  | Turkey | IRMA | BPH | NS |  | 53-85 | 51-79 |  | Not Stated | √ |  |  |  | √ |  | √ |  | Means |  | Serious |
| Oliver (2004) | ProtecT | UK | ELISA | Mixed | PBC |  | 62.2 | 62.2 |  | Caucasian | √ | √ |  | √ | √ |  | √ | √ | Quantiles | √ | Moderate |
| Trapeznikova (2004) |  | Russia | ELISA | BPH | NS |  | 66.6 | 60.3 |  | Not Stated | √ | √ |  |  |  |  |  |  | Excluded |  | Critical |
| Lopez (2004) |  | Malaysia | ELISA | Healthy | NS |  | 69.7 | 57.2 |  | Not Stated | √ |  |  |  | √ |  |  |  | Excluded |  | Critical |
| Kehinde (2005) |  | Kuwait/Oman | IFMA | Healthy | P | 69.7 | 15-90 | 15-90 |  | Caucasian | √ |  |  |  | √ |  |  |  | Excluded |  | Critical |
| Marszalek (2005) |  | Austria | IRMA | Mixed | NS | 66.7 | 67 | 69 |  | Not Stated | √ |  |  |  |  |  |  |  | Means |  | Moderate |
| Nam (2005) | University Health Network | Canada | ELISA | Mixed | TC |  | 66.6 | 65.5 |  | Multi-ethnic | √ |  |  |  | √ |  |  |  | Means |  | Serious |
| Trojan (2006) |  |  | ELISA | BPH | H |  | 62.8 | 66.8 |  | Not Stated |  | √ |  |  |  |  |  |  | Means |  | Serious |
| Hernandez (2007) |  | USA | Other | Healthy | H |  | 65.86 | 68.85 |  | Black | √ |  |  |  | √ | √ | √ |  | Quantiles | √ | Moderate |
| Zhigang (2007) |  | China | ELISA | Mixed | H |  | 65.5 | 65.1 |  | Asian | √ |  |  |  | √ |  |  |  | Means |  | Moderate |
| Borugian (2008) | Prospective Multiethnic Study | Hawaii, USA, Canada | ELISA | Mixed | P |  | 69.1 | 68.9 | 1+ | Multi-ethnic | √ |  |  |  | √ |  |  |  | Quantiles | √ | Moderate |
| Hong (2008) |  | Korea | IRMA | Healthy | H |  | 65 | 64 |  | Asian | √ |  |  |  | √ |  |  |  | Means |  | Serious |
| Sciarra (2008) |  | Italy | ELISA | BPH | H |  | 67.24 | 67.06 |  | Not Stated | √ |  |  |  |  |  |  |  | Means |  | Serious |
| Jeong*** (2009) |  | Korea | ELISA | Healthy | P |  | 63.5 | 63.1 |  | Asian | √ |  |  |  | √ |  | √ |  | Quantiles | √ | Moderate |
| Pina (2009) |  | Portugal | ELISA | BPH | NS |  | 69 | 67 |  | Not Stated | √ |  |  |  |  |  |  |  | Means |  | Serious |
| Johansson (2009) | CAPS Study | Sweden |  | Healthy | P |  | M 5yr | M 5yr |  | Caucasian |  |  |  |  | √ | √ |  |  | Means | √ | Moderate |
| Gill (2010) | MEC | USA | ELISA | Mixed | P |  | 68.9 | 68.7 |  | Multi-ethnic | √ | √ | √ |  | √ |  | √ |  | Quantiles | √ | Moderate |
| Kim (2010) |  | Korea | ELISA | Mixed | H | Not stated | | |  | Not Stated |  |  |  | √ | √ |  |  |  | Rowlands data | √ | Moderate |
| Park*** (2010) |  | Korea | ELISA | Healthy | H |  | 64.7 | 63.5 |  | Asian |  |  |  |  | √ | √ |  |  | Means | √ | Moderate |
| Tajtakova (2010) |  | Slovakia | RIA | Healthy | NS |  | 65.5 | 60.7 |  | Not Stated | √ |  |  |  | √ |  |  |  | Means |  | Serious |
| Campa (2011) | EPIC | Europe | ELISA | Mixed | PBC | 60.4 | 60.4 | 60.5 | 1* | Caucasian | √ |  |  |  | √ |  |  |  | None: RCD |  | Moderate |
| Darago (2011) |  | Poland | CLA | BPH | H |  | 70.2 | 70.1 |  | Not Stated | √ |  |  |  | √ |  |  |  | Means |  | Serious |
| Safarinejad (2011) |  | Iran | ELISA | Healthy | H |  | 63.6 | 62.5 |  | Caucasian | √ |  |  |  | √ | √ |  |  | Means | √ | Moderate |
| Rowlands (2012) | ProtecT | UK | RIA | Mixed | PBC |  | 61.9 | 61.7 |  | White | √ | √ |  | √ | √ |  |  | √ | Continuous | √ | Moderate |
| Neuhouser (2013) | PCPT | USA | ELISA | Mixed | TC |  | 63.6 | 63.6 |  | White | √ | √ |  | √ | √ |  |  | √ | Means | √ | Moderate |
| Iltaf (2013) |  | Karachi | ELISA | Healthy | P |  |  | 50+ |  | Asian | √ |  |  |  |  |  |  |  | Excluded |  | Critical |
| ***Genetic data only*** | |  |  |  |  |  |  |  |  |  |  |  |  |  |  |  |  |  |  |  |  |
| Ho (2003) |  | USA |  | Mixed | H | 63 |  |  |  | Multi-ethnic |  |  |  |  |  | √ |  |  | Categorical | √ | Low |
| Wang (2003) |  | Japan |  | Healthy | P |  |  |  |  | Multi-ethnic |  |  |  |  |  | √ |  |  | Categorical | √ | Low |
| Li (2004) |  | USA |  | Mixed |  |  |  |  |  | Multi-ethnic |  |  |  |  |  | √ |  |  | Categorical | √ | Low |
| Friedrichsen  (2005) | Seattle-Puget Sound Registry SEER | USA |  | Mixed | P |  |  |  |  | Multi-ethnic |  |  |  |  |  | √ |  |  | Categorical | √ | Low |
| Neuhausen  (2005) |  | University of Utah, USA |  | Mixed | P | 63 |  |  |  | Multi-ethnic |  |  |  |  |  | √ |  |  | Categorical | √ | Unclear |
| Schildkraut (2005) |  | USA |  | Mixed | P | 62.7 |  |  |  | Multi-ethnic |  |  |  |  |  | √ |  |  | Categorical | √ | Unclear |
| Tsuchiya (2005) |  | Japan |  | Healthy | H |  |  |  |  | Caucasian |  |  |  |  |  | √ |  |  | Categorical | √ | Low |
| Chen (2006) | Cardiovascular Health Study | USA |  | Mixed | PBC |  |  |  |  | Multi-ethnic |  |  |  |  |  | √ |  |  | Categorical | √ | Low |
| Cheng (2006) | MEC | USA |  | Mixed | PBC |  |  |  |  | Caucasian |  |  |  |  |  | √ |  |  | Categorical | √ | Unclear |
| Cheng (2006) | MEC | USA |  | Mixed | PBC | 68.3 |  |  |  | African-American |  |  |  |  |  | √ |  |  | Categorical |  | Unclear |
| Hoyo (2007) |  | USA |  | Mixed | H |  |  |  |  | Multi-ethnic |  |  |  |  |  | √ |  |  | Categorical | √ | Low |
| Johansson (2007) | CAPS Study | Sweden |  | Healthy | P |  |  |  |  | Caucasian |  |  |  |  |  | √ |  |  | Categorical |  | Unclear |
| Sarma (2008) | Flint Men's Health Study | USA |  | Mixed | P |  |  |  |  | Asian |  |  |  |  |  | √ |  |  | Categorical | √ | Critical |
| Schumacher (2010) | BPC3 | Mixed | ELISA | Mixed | PBC | 68 |  |  |  | Asian |  |  |  |  |  | √ |  |  | Categorical | √ | Low |
| *BPH: Benign prostatic hyperplasia; M: Matched; H: Hospital; P: Population; PBC: Population-based cohort; RCD: Repeated cohort data; S: Sibling; TC: Trial cohort. ATBC: Alpha-Tocopherol, Beta-Carotene Cancer Prevention; BPC3: NCI Breast and prostate cancer consortium; CAPS: Cancer of the Prostate in Sweden; EPIC: European Prospective Investigation into Cancer and Nutrition; MEC: Multi-ethnic cohort; NSHDS; Northern Sweden Health and Disease Cohort Study; PCPT: Prostate cancer prevention trial; PLCO: Prostate, Lung, Colorectal and Ovarian. *Minimum number of years between sample collection and diagnosis. **Unclear if retrospective or prospective study; therefore, grouped as retrospective ***Unclear whether PSA-screened or not. ^1^IGF-I only ^2^RFPC study only* | | | | | | | | | | | | | | | | | | | | | |

| Supplementary Table 2. List of polymorphisms presented in each genetic paper (highlighted columns represent those polymorphisms with sufficient data to be analysed. | | | | | | | | | | | | | | | | | | | | | | | | | | | | | | | | | | | | | | | | | | | | | | | | |
| --- | --- | --- | --- | --- | --- | --- | --- | --- | --- | --- | --- | --- | --- | --- | --- | --- | --- | --- | --- | --- | --- | --- | --- | --- | --- | --- | --- | --- | --- | --- | --- | --- | --- | --- | --- | --- | --- | --- | --- | --- | --- | --- | --- | --- | --- | --- | --- | --- |
| **Author (year)** | **IGF-1 (CA)n*** | **IGF-1 hCV2801089** | **IGF-1 hCV2801106** | **IGF-1 hCV2801114** | **IGF-1 hCV3061153** | **IGF1 rs10735380** | **IGF-1 rs1106381** | **IGF-1 rs1457601** | **IGF1 rs1520220** | **IGF-1 rs1520220** | **IGF-1 rs2033178** | **IGF-1 rs2072952** | **IGF-1 rs2288377** | **IGF-1 rs2946834** | **IGF-1 rs4764695** | **IGF-1 rs4764876** | **IGF-1 rs5742639** | **IGF-1 rs5742657** | **IGF-1 rs5742723** | **IGF-1 rs6218** | **IGF-1 rs6220** | **IGF-1 rs7136446** | **IGF-1 rs7965399** | **IGF-1 rs7978742** | **IGF-1R (AGG)n** | **IGF-1R deletion** | **IGF2-Msp1 +3580** | **IGFALS rs1178436** | **IGFALS rs11865665** | **IGFALS rs17559** | **IGFALS rs344352** | **IGFBP1 rs1065780** | **IGFBP1 rs1995051** | **IGFBP1 rs3763497** | **IGFBP1 rs4988515** | **IGFBP1 rs9658194** | **IGFBP3 -202A/C*** | **IGFBP3 rs2132570** | **IGFBP3 rs2132571** | **IGFBP3 rs2270628** | **IGFBP3 rs2453839** | **IGFBP3 rs2471551** | **IGFBP3 rs2854742** | **IGFBP3 rs2854744** | **IGFBP3 rs2854746** | **IGFBP3 rs2960436** | **IGFBP3 rs3110697** | **IGFBP3 rs6670** |
| Chen (2006) | √ |  |  |  |  |  |  |  |  |  |  |  |  |  |  |  |  |  |  |  |  |  |  |  | √ | √ |  |  |  |  |  |  |  |  |  |  | √ |  |  |  |  |  |  |  |  |  |  |  |
| Cheng (2006) |  |  |  |  |  |  |  |  |  |  |  |  |  |  |  |  |  |  |  |  |  |  |  |  |  |  |  |  |  |  |  |  |  |  |  |  | √ |  |  |  |  |  |  |  |  |  |  |  |
| Cheng (2006) |  | √ | √ | √ | √ |  | √ | √ |  | √ |  | √ | √ | √ |  | √ | √ | √ | √ | √ |  |  | √ | √ |  |  |  |  |  |  |  |  |  |  |  |  |  |  |  |  |  |  |  |  |  |  |  |  |
| Friedrichsen (2005) | √ |  |  |  |  |  |  |  |  |  |  |  |  |  |  |  |  |  |  |  |  |  |  |  |  |  |  |  |  |  |  |  |  |  |  |  |  |  |  |  |  |  | √ |  |  |  |  |  |
| Hernandez (2007) | √ |  |  |  |  |  |  |  |  |  |  |  |  |  |  |  |  |  |  |  |  |  | √ |  |  |  |  |  |  |  |  |  |  |  |  |  | √ |  |  |  |  |  |  |  |  |  |  |  |
| Ho (2003) |  |  |  |  |  |  |  |  |  |  |  |  |  |  |  |  |  |  |  |  |  |  |  |  |  |  | √ |  |  |  |  |  |  |  |  |  |  |  |  |  |  |  |  |  |  |  |  |  |
| Hoyo (2007)** | √ |  |  |  |  |  |  |  |  |  |  |  |  |  |  |  |  |  |  |  |  |  |  |  |  |  |  |  |  |  |  |  |  |  |  |  | √ |  |  |  |  |  |  |  |  |  |  |  |
| Johansson (2009) |  |  |  |  |  |  |  |  |  |  |  |  |  |  |  |  |  |  |  |  |  |  |  |  |  |  |  |  |  |  |  | √ | √ | √ | √ | √ |  |  | √ |  | √ | √ |  | √ |  |  |  | √ |
| Johansson (2007) |  |  |  |  |  |  |  |  |  |  | √ |  |  |  |  |  |  |  |  |  | √ | √ |  |  |  |  |  |  |  |  |  |  |  |  |  |  |  |  |  |  |  |  |  |  |  |  |  |  |
| Li (2004)** | √ |  |  |  |  |  |  |  |  |  |  |  |  |  |  |  |  |  |  |  |  | √ |  |  |  |  |  |  |  |  |  |  |  |  |  |  | √ |  |  |  |  |  |  |  |  |  |  |  |
| Neuhausen (2005)** | √ |  |  |  |  |  |  |  |  |  |  |  |  |  |  |  |  |  |  |  |  |  |  |  |  |  |  |  |  |  |  |  |  |  |  |  |  |  |  |  |  |  |  |  |  |  |  |  |
| Park (2010) |  |  |  |  |  |  |  |  |  |  |  |  |  |  |  |  |  |  |  |  |  |  |  |  |  |  |  |  |  |  |  |  |  |  |  |  | √ |  |  |  |  |  |  |  |  |  |  |  |
| Safarinejad (2011) |  |  |  |  |  |  |  |  |  |  |  |  |  |  |  |  |  |  |  |  |  |  |  |  |  |  |  |  |  |  |  |  |  |  |  |  | √ |  |  |  |  |  |  |  |  |  |  |  |
| Schildkraut (2005) | √ |  |  |  |  |  |  |  |  |  |  |  |  |  |  |  |  |  |  |  |  |  |  |  |  |  |  |  |  |  |  |  |  |  |  |  | √ |  |  |  |  |  | √ |  |  |  |  |  |
| Schumacher (2010) |  |  |  |  |  |  |  |  |  |  |  |  |  |  | √ |  |  |  |  |  |  |  |  |  |  |  |  |  |  |  |  |  |  |  |  |  |  |  |  |  |  |  |  |  |  |  |  |  |
| Tsilidis (2013) |  |  |  |  |  | √ |  |  | √ |  |  |  |  |  |  |  |  |  |  |  |  |  |  |  |  |  |  | √ | √ | √ | √ |  |  |  |  |  |  | √ |  | √ |  |  |  | √ | √ | √ | √ |  |
| Tsuchiya (2005) | √ |  |  |  |  |  |  |  |  |  |  |  |  |  |  |  |  |  |  |  |  |  |  |  |  |  |  |  |  |  |  |  |  |  |  |  |  |  |  |  |  |  |  |  |  |  |  |  |
| Wang (2003) |  |  |  |  |  |  |  |  |  |  |  |  |  |  |  |  |  |  |  |  |  |  |  |  |  |  |  |  |  |  |  |  |  |  |  |  | √ |  |  |  |  |  |  |  |  |  |  |  |
| **Only IGF-1(CA)_n_ and IGFBP-3 -202A/C were meta-analysed.*  ***Could not be used in meta-analysis as data was un-combinable (Li (2004) had combinable IGFBP-3 data, but un-combinable IGF-I data)* | | | | | | | | | | | | | | | | | | | | | | | | | | | | | | | | | | | | | | | | | | | | | | | | |

Supplementary Table 3: Studies investigating tissue expression of IGF system and prostate cancer included as supporting evidence.

| **Study** | **Year** | **Experiments** | **Samples** | **Statistical analysis** | **Results** |
| --- | --- | --- | --- | --- | --- |
| **IGF-I** | | | | | |
| Mita | 2000 | **qPCR** analysis of mRNA of IGF system | 24 prostatectomy specimens after neoadjuvant hormone therapy  No controls included. | Mann-Whitney U-test | IGF-I mRNA was lower in locally advanced prostate cancer than in early stage (p=0.038), but was not related to LN mets, histologic differentiation or serum PSA level. |
| Cardillo | 2003 | **IHC**, **ISH** and **qPCR** of IGF-I, IGF-II and IGF-IR protein and mRNA expression in PCa, PIN and NAP | Prostatectomy specimens  Control: PIN and NAP | Mean +/- SEM, Chi-square, Student’s paired t-test, Mann-Whitney U-test, Spearman rank correlation and linear regression  , ANOVA | IGF-I protein increased from NAP tissue to PIN, to PCa tissue in the epithelial (p<0.0001) and stromal (p<0.0146).  No correlation between IGF-I (protein and mRNA) and Gleason histological score or TNM stage. |
| Soulitzis | 2006 | **qPCR** to determine mRNA expression levels of IGF-I in tissue specimens from patients with PCa, or BPH, and normal prostate samples obtained post-mortem from young individuals | Patients with PCa (n=42)  BPH: (n=42)  Controls: young individuals (n=10) | Chi-squared test and Fisher’s exact test | PCa patients with low Gleason score (<7) have increased IGF-I (p=0.031) mRNA levels. IGF-I levels are also elevated in tumors with TNM stages T1-T2 (p=0.03). |
| Massoner | 2011 | **IHC** and **qPCR** of IGF axis (IGF-I, IGF-II, IGFBP 1-6 and insulin receptor) in microdisseced tissue specimens of local PCa | Set 1: 20 local PCa specimens assigned according to their Gleason score used for laser microdissection  Set 2: 10 local PCa specimens  Set 3: 22 samples for IHC together with 20 samples from set 1  Benign tissues (from prostatectomy specimens) | Spearman’s p test for correlation, Mann-Whitney U-test and student’s t-test | IGF-I mRNA expression was decreased in prostate cancer compared with benign prostate areas.  IGF-I mRNA was decreased in high-grade (GSC 8–10) compared with low- grade (GSC 5– 6) cancer.  IGF-I protein levels determined by IHC do not reflect mRNA expression levels in PCa. |
| Savvani | 2013 | **IHC** of IGF-IEc expression in prostate cancer specimens | 83 prostatectomy specimens  No controls | Shapiro-Wilk test, Student’s t-test, ANOVA and spearman correlation coefficient | Mean IGF-1Ec expression was lower in localized (stage ≤IIb) PCa compared to locally advanced tumours (stage ≥III) (p=0.004).  Weak positive correlation was observed between IGF-IEc expression and Gleason score (p=0.02).  No association between IGF-IEc expression and age (p=0.81), PIN (p=0.153), positive surgical margins (p=0.95), vascular invasion (p=0.347), perineural invasion (p=0.185) and tumour extent inside the prostate gland (p=0.18). |

| **Study** | **Year** | **Experiments** | **Samples** | **Statistical analysis** | **Results** |
| --- | --- | --- | --- | --- | --- |
| **IGF-II** | | | | | |
| Tennant | 1996 | **IHC** and **ISH** to compare the expression of IGF-IR and IGF-II in benign epithelium, HG-PIN and prostate adenocarcinoma | 32 prostatectomy specimens | ANOVA, Fisher’s exact test and paired t-tests | IGF-II mRNA was increased by 30% in adenocarcinoma compared to benign epithelium (p<0.03) but not IGF-II protein. |
| Mita | 2000 | **qPCR** analysis of mRNA of IGF system | 24 prostatectomy specimens after neoadjuvant hormone therapy; No controls included. | Mann-Whitney U-test | IGF-II mRNA was associated with pathologic stage (p=0.003), LN mets (p=0.0007), histologic differentiation (p=0.003) and serum PSA level (p=0.04) after hormone therapy. |
| Cardillo | 2003 | **IHC**, **ISH** and **qPCR** of IGF-I, IGF-II and IGF-IR protein and mRNA expression in PCa, PIN and NAP | Prostatectomy specimens  Control: PIN and NAP | Mean +/- SEM, Chi-square, Student’s paired t-test, Mann-Whitney U-test, Spearman rank correlation and linear regression and ANOVA | IGF-II protein increased as the prostate tissue progressed from NAP, to PIN and PCa in both epithelium (p<0.0001) and stroma (p=0.033).  IGF-II mRNA increased as the prostate tissue progressed from NAP, to PIN and PCa in the epithelium (p<0.0001) and in the stroma (p=0.04).  No correlation was found between TNM stage and IGF-II expression.  High Gleason score tumors (8,9) expressed IGF-II mRNA and protein more strongly than lower Gleason score tumors, in the epithelium (IGF-II protein 2.85±0.14 vs 1.90±0.28, p=0.012; IGF-II mRNA 2.62±0.10 vs 1.96±0.22, p=0.03) and in the stroma (IGF-II protein 2.71±0.18 vs 2.06±0.22, p = 0.048; IGF-II mRNA 2.23±0.1 vs 1.69±0.2, p=0.05). |
| Massoner | 2011 | **IHC** and **qPCR** of IGF axis (IGF-I, IGF-II, IGFBP 1-6 and insulin receptor) in microdisseced tissue specimens of local PCa | Set 1: 20 local PCa specimens assigned according to their Gleason score used for laser microdissection; Set 2: 10 local PCa specimens; Set 3: 22 samples for IHC together with 20 samples from set 1  Control: Benign tissues (from prostatectomy specimens) | Spearman’s p test for correlation, Mann-Whitney U-test and student’s t-test | IGF-II mRNA expression was decreased in PCa compared with benign prostate areas.  IGF-II mRNA was decreased in high-grade (GSC 8–10) compared with low- grade (GSC 5– 6) cancer.  IGF-II protein levels determined by IHC do not reflect mRNA expression levels in PCa. |

| **Study** | **Year** | **Experiments** | **Samples** | **Statistical analysis** | **Results** |
| --- | --- | --- | --- | --- | --- |
| **IGF-IR** | | | | | |
| Tennant | 1996 | **IHC** and **ISH** to compare the expression of IGF-IR and IGF-II in benign epithelium, HG-PIN and prostate adenocarcinoma | 32 prostatectomy specimens | ANOVA, Fisher’s exact test and paired t-tests | IGF-IR mRNA and protein was decreased in PIN and in PCa compared to benign epithelium (mRNA: p<0.0001; protein: p<0.0004). |
| Cardillo | 2003 | **IHC**, **ISH** and **qPCR** of IGF-I, IGF-II and IGF-IR protein and mRNA expression in PCa, PIN and NAP | Prostatectomy specimens  Control: PIN and NAP | Mean +/- SEM, Chi-square, Student’s paired t-test, Mann-Whitney U-test, Spearman rank correlation and linear regression and ANOVA | IGF-IR mRNA expression increased from NAP to PIN to PCa in the epithelial (p<0.0001) and stromal (p=0.001).  IGF-IR protein increased in PCa than PIN and normal epithelial cells (p<0.0001) |
| Ryan | 2007 | **IHC** of IGF-IR expression in normal prostate epithelium and prostate cancer specimens | 30 primary prostate tumours  5 locally recurrent androgen-independent tumors; 5 distant androgen-independent lymph node metastases  Control: benign prostates from men without PCa | Mann Whitney test and Fisher exact test | IGF-IR protein was expressed in both normal prostate epithelium and PCa.  No associations between the Gleason grade and absence or presence of the IGF-IR (p =0.17 for normal epithelium in men with cancer; p= 0.26 for PCa).  No associations observed between stromal staining and tumour stage (T1c vs. T2–T4, P =1.0), race (white vs. non- white, P=0.71), age (P= 0.49), or PSA level (P= 0.56). |
| Massoner | 2011 | **IHC** and **qPCR** of IGF axis (IGF-I, IGF-II, IGFBP 1-6 and insulin receptor) in microdisseced tissue specimens of local PCa | Set 1: 20 local PCa specimens assigned according to their Gleason score used for laser microdissection; Set 2: 10 local PCa specimens; Set 3: 22 samples for IHC together with 20 samples from set 1  Control: Benign tissues from the prostatectomy specimens | Spearman’s p test for correlation, Mann-Whitney U-test and student’s t-test | IGF-IR mRNA was decreased in PCa compared with benign epithelial cells.  . |
| Turney | 2011 | IHC of IGF-IR expression in serial prostate cancer specimens | 18 patients who had undergone serial channel TURP at least 3 months apart.  Controls: prostate cancer sections that had previously stained heavily for IGF-IR | Not stated | No correlation between Gleason sum score and IGF-1R protein.  6/7 patients with falling IGF-1R staining scores were responding to androgen deprivation therapy (confirmed by PSA response) between operations. 7/8 patients who had progression to androgen-independence between procedures, IGF-1R levels increased or remained high.  7/11 patients developed radiologically confirmed metastases between procedures showed stable or increasing IGF-IR staining. |

| **Study** | **Year** | **Experiments** | **Samples** | **Statistical analysis** | **Results** |
| --- | --- | --- | --- | --- | --- |
| **IGF-IR (Continued)** | | | | | |
| Hetzl | 2012 | IHC of IGF-IR expression in both prostatic stromal and epithelial. | 45 prostatic samples from PCa patients obtained by radical prostatectomy  Control: 15 prostatic samples were obtained from necropsied patients without a diagnosis of prostatic or other urological diseases | ANOVA and post-hoc Tukey’s test | Increased IGF-IR protein levels in PCa and HG-PIN compared to normal and BPH group (p<0.01). |
| Zu | 2013 | **IHC** of IGF-IR expression in PCa to investigate if IGF-IR is a potential effect modifier for the association between PTEN expression and lethal prostate cancer risk. | 651 men with PCa | Logistic regression multivariate Cox models and Wald test | High IGF-IR protein alone was associated fatal prostate cancer or distant metastasis (HR:13.8; 95% CI, 1.7–112.8).  A significant negative interaction between PTEN and IGF-IR was found (Pinteraction=0.03). |
| **IGFBP-2** | | | | | |
| Tennant | 1996 | **IHC** and **ISH** was carried out to compare expression of IGFBP-2 and 3 in PCa and HG-PIN | 28 prostatectomy specimens from prostate adenocarcinoma  Benign (n=28); HG-PIN (n=8); PCa (n=11) | ANOVA, Fisher’s exact test, linear regression and paired t-tests | IGFBP-2 protein and mRNA was increased in PIN (p<0.0003) and PCa (p<0.0003). |
| Thrasher | 1996 | **IHC** of IGFBP-2 and IGFBP-3 protein expression in prostate tissues | 24 patients who underwent radical prostatectomy for localized prostate adenocarcinoma | ANOVA and Fisher’s protected least squared difference post-hoc test : compare IGFBP-2 and 3 among different groups. | IGFBP-2 protein was increased in PIN (p<0.001), and PCa (p<0.001) compared to normal epithelium and increased in PCa compared to PIN (p <0.05).  No correlation between the stage and grade of prostate cancer and IGFBP-2 immunostaining intensity (p = 0.69 and 0.48, respectively)  No correlation between preoperative serum PSA determinations and IGFBP-2 immunoreactivity (p= 0.99) |
| Figueroa | 1998 | IGFBP 1-6 RNA expression in benign and neoplastic prostate tissue detected by **RNAse protection assay** | 23 consecutive radical prostatectomy specimens  High Gleason scores (7-10): n=6; Low or intermediate scores (2-6): n=17  Control: Benign prostate tissue  (Note: samples obtained from the same prostatectomy specimens) | One-tailed Student’s t-test | Tumours with high Gleason score expressed higher IGFBP-2 RNA levels (0.4555 (CI: 0.395-0.515) vs 0.3 (CI: 0.256-0.356) (p<0.002).  High Gleason score tumours had a 117% and 95% higher IGFBP-2/IGFBP-3 RNA expression ratio compared with benign and low grade tumours, respectively. |

| **Study** | **Year** | **Experiments** | **Samples** | **Statistical analysis** | **Results** |
| --- | --- | --- | --- | --- | --- |
| **IGFBP-2 (Continued)** | | | | | |
| Mita | 2000 | **qPCR** analysis of mRNA of IGF system | 24 prostatectomy specimens after neoadjuvant hormone therapy  No controls included. | Mann-Whitney U-test | IGFBP-2 mRNA was associated with pathologic stage (p=0.002), LN Mets (p=0.001), histologic differentiation (p=0.002) and serum PSA level (p=0.002) after hormone therapy. |
| Ambriosini-Spaltro | 2001 | **IHC** of IGFBP-2 in normal epithelium, HG-PIN and Prostate adenocarcinoma | Prostatectomy specimens (n=60)  Group 1: Patients with bladder outlet obstruction  Group 2: Patients hormonally untreated before surgery  Group 3: Patients who underwent complete androgen ablation 3 months before surgery  Control: HGPIN and normal epithelium | ROC curves: % of positive neoplastic cells  Wilcoxon signed rank test: % of +ve neoplastic cells  Spearman rank test: Gleason scores, pathologic stages and IHC scores | IGFBP-2 is expressed in the cytoplasm of untreated PCa and to a lesser extent in HG-PIN.  IGFBP-2 is expressed in PCa and HG-PIN after complete androgen ablation, but to a lesser extent than in the untreated neoplasms.  IGFBP-2 expression in the untreated specimens is lower in HG-PIN than in invasive PCa.  IGFBP-2 was positive in PCa cases but not in benign prostatic tissues.  No correlation between IGFBP2 with Gleason Grade or Tumour stage. |
| Richardsen | 2003 | **IHC** of IGFBP-2 expression in high-grade PIN, and PCa | 193 radical prostatectomy specimens from patients with localized prostate adenocarcinoma  Controls: BPH (n=14) | Fisher’s exact probability test | Significant overexpression of IGFBP-2 in all instances of PIN and in more than 90% of cancers regardless of the grade. .  The majority of cases with invasive carcinoma showed an overexpression of IGFBP-2 in >90% of the cancer cells.  Extent and pattern of IGFBP2 expression was not correlated with Gleason grade. |
| Massoner | 2011 | **IHC** and **qPCR** of IGF axis (IGF-I, IGF-II, IGFBP 1-6 and insulin receptor) in microdisseced tissue specimens of local PCa | Set 1: 20 local PCa specimens assigned according to their Gleason score used for laser microdissection  Set 2: 10 local PCa specimens  Set 3: 22 samples for IHC together with 20 samples from set 1  Benign tissues  Note: samples obtained from the prostatectomy specimens) | Spearman’s p test for correlation, Mann-Whitney U-test and student’s t-test | IGFBP-2 mRNA expression levels unchanged in prostate cancer tissue areas compared with benign prostate tissue areas. |

| **Study** | **Year** | **Experiments** | **Samples** | **Statistical analysis** | **Results** |
| --- | --- | --- | --- | --- | --- |
| **IGFBP-3** | | | | | |
| Tennant | 1996 | **IHC** and **ISH** was carried out to compare expression of IGFBP-2 and 3 in PCa and HG-PIN | 28 prostatectomy specimens from prostate adenocarcinoma  Benign (n=28); HG-PIN (n=8); PCa (n=11) | ANOVA, Fisher’s exact test, linear regression and paired t-tests | IGFBP-3 mRNA was unchanged in benign epithelium, PIN and PCa.  IGFBP-3 protein was increased in PIN (p<0.0001) but as decreased in malignant cells (p<0.0001). |
| Thrasher | 1996 | **IHC** of IGFBP-2 and IGFBP-3 protein expression in prostate tissues | 24 patients who underwent radical prostatectomy for localized prostate adenocarcinoma | ANOVA and Fisher’s protected least squared difference post-hoc test : compare IGFBP-2 and 3 among different groups. | IGFBP-3 protein was decreased in PCa carcinoma compared to normal epithelium (p <0.0001).  IGFBP-3 protein increased in PIN compared to normal epithelium (p<0.001).  No correlation between the stage and grade of prostate cancer and IGFBP-3 protein (p = 0.88 and 0.52, respectively)  No correlation between preoperative serum PSA determinations and IGFBP-3 immunoreactivity (p=0.21). |
| Figueroa | 1998 | IGFBP 1-6 RNA expression in benign and neoplastic prostate tissue detected by **RNAse protection assay** | 23 consecutive radical prostatectomy specimens  High Gleason scores (7-10): n=6; Low or intermediate scores (2-6): n=17  Control: Benign prostate tissue from the same prostatectomy specimens | One-tailed Student’s t-test | IGFBP-3 RNA levels lower in high Gleason score tumours (p=0.05). |
| Hampel | 1998 | **IHC** of IGFBP-3 in prostate adenocarcinoma | Study 1: 20 neoplastic prostates; Control: 6 normal prostates obtained from patients undergoing cystoprostatectomy for bladder cancer  Study 2: 24 radical prostatectomy specimens from patients with clinically localized prostate adenocarcinoma Control: 8 normal prostates from organ donors or from patients undergoing cysto-prostatectomy for bladder cancer. | Conventional non-parametric tests, including the Wilcoxon sign rank test and the Mann Whitney U test.  Mixed model was fit to the repeated measures data | IGFBP-3 protein was decreased in PCa compared to normal epithelium (p<0.0001).  IGFBP-3 protein was not associated with Gleason grade, recurrence or LN mets. |

| **Study** | **Year** | **Experiments** | **Samples** | **Statistical analysis** | **Results** |
| --- | --- | --- | --- | --- | --- |
| **IGFBP-3 (Continued)** | | | | | |
| Linos | 2009 | **IHC** of IGFBP-3 expression in prostatic adenocarcinoma samples | 199 prostatectomy  specimens.  No control specimens from healthy controls. | No stated | Cytoplasmic IGFBP-3 over expression was observed in 119/199 (60%) of all tumors.  Within the non-treated subgroup (n=144), cytoplasmic IGFBP-3 over expression correlated with high tumor Gleason grade (Gleason score of 7 or more) [65% high grade versus 48% low grade, p=0.042].  Trend towards advanced stage, with 66% advanced stage tumors over expressing IGFBP3 protein versus 51% organ confined tumors, p=0.10). |
| Seligson | 2013 | **IHC** of nuclear and cytoplasmic IGFBP-3 protein expression in PCa | 226 prostatectomy specimens of prostate adenocarcinoma obtained from randomly selected, hormone naïve patients  Median Age: 65 (range: 46-76).  Control: Matched benign (morphologically normal or hypertrophic) or in situ neoplastic lesions (PIN) obtained from prostate cancer patients | Kruskal-Wallis and Mann Whitney U tests: differences between nominal clinicopathologic prognostic variables.  Chi-squared test: association of dichotomized IGFBP-3 versus nominal variables.  Kaplan-Meier plots and Cox proportional Hazards regression models: association with recurrence-free time. | Higher IGFBP-3 cytoplasmic and nuclear staining in PCa than benign tissues (p<0.0001).  Expression of nuclear IGFBP-3 was associated with disease recurrence as both a continuous p=0.0039, HR: 1.03; 95% CI: 1.01-1.06) and a dichotomized (p=0.0033; 2.51; 95% CI: 1.36-4.63) variable.  Median recurrence-free time was 60 months for cases with high nuclear IGFBP-3 (n=71), and the median was not reached for cases with low nuclear IGFBP-3 (n=123) (Logrank p=0.0074).  In patients with primary low-grade cancer, the presence of nuclear IGFBP-3 was even more predictive of tumor recurrence than in all cases (Logrank p=0.0007).  In the low-grade group, IGFBP-3 mean positivity association with recurrence (p=0.0007, HR: 1.03; 95% CI: 1.03-1.10), and of dichotomized data (p=0.001; 5.44; 95% CI: 1.99-14.87). |

Note: PCa: Prostate cancer; HG-PIN: High grade prostatic intraepithelial neoplasia; IHC: immunohistochemistry; ISH: in-situ hybridisation; NAP: normal adjacent counterpart; SEM: standard error of mean; qPCR: quantitative reverse-transcription polymerase chain reaction; BPH: Benign prostatic hyperplasia; ChIP: Chromatin immunoprecipitation; ab: antibody; ELISA: enzyme-linked immunosorbent assay; PSA: prostate specific antigen; TURP: transurethral resection of the prostate; MALDI-TOF: matrix-assisted laser desorption/ionization-time of flight; PCR: polymerase chain reaction; DRE: digital rectal examination; OR: Odds ratio; CI: confidence interval; LN mets: lymph node metastasis

Supplementary Table 4: Studies investigating whether genetic or epigenetic changes in the IGF system is associated with prostate cancer

| **Study** | **Year** | **Experiments** | **Samples** | **Experimental procedures** | **Statistical analysis** | **Results** |
| --- | --- | --- | --- | --- | --- | --- |
| **IGF-I** | | | | | | |
| Cheng | 2006 | Investigated whether genetic variation at the IGF-I locus is associated with prostate cancer risk. | Sequencing: Men with advanced PCa (n=95)  Genotyping Case-control study: Men with PCa (n=2320)  Controls: men without PCa (n=2290) | Sequenced IGF-I exons in germline DNA  Genotyping of tagged SNPs | Unconditioned logistic regression: association between PCa and IGF-I haplotypes and genotypes and permutation tests | Haplotype analysis revealed nominally statistically significant associations with PCa risk in each of the four haplotype blocks: haplotype 1B (OR = 1.21, 95% CI = 1.04 to 1.40), haplotype 2C (OR = 1.24, 95% CI = 1.06 to 1.44), haplotype 3C (OR = 1.25, 95% CI = 1.03 to 1.50), and haplotype 4D (OR = 1.19, 95% CI = 1.02 to 1.39).  SNP3 (rs7978742) and SNP4 (rs7965399) was associated with prostate cancer risk (P_trend_ = .002). The CT genotype for SNP4 was associated with increased risk of prostate cancer, compared with the common homozygous TT genotype (OR = 1.25, 95% CI = 1.09 to 1.43; P = .001). This association was also statistically significant for non-advanced disease (OR = 1.32, 95% CI = 1.13 to 1.55; P<0.001). |
| Tsuchiya | 2006 | To examine the association of 13 genetic polymorphisms with survival of metastatic PCa patients. | 111 PCa patients with bone metastasis at diagnosis and not received treatment | Polymerase chain reaction-restriction fragment length polymorphism or automated sequencer with genotyping | Kaplan-Meier curve, log rank test and cox proportional hazards model | Long allele (over 18 [CA] repeats) of insulin-like growth factor-I (IGF-I) was significantly associated with a worse cancer-specific survival (P =0.025).  Long allele of IGF-I polymorphisms was an independent risk factor for death (HR: 2.01: 95% CI: 1.12-3.62;p =0.019). |
| Tsuchiya | 2013 | To evaluate the association of polymorphisms in 3 linkage disequilibrium blocks of IGF-I on survival of metastatic PCa patients. | 215 patients with bone metastasis at initial presentation | Polymerase chain reaction-restriction fragment length polymorphism or automated sequencer with genotyping | Kaplan-Meier curve, log rank test and cox proportional hazards model | CA repeat polymorphism, rs12423791 and rs6220 are associated with cancer-specific survival (p=0.013, 0.014 and 0.014, respectively).  Haplotype in LD block 3 was significantly associated with cancer–specific survival (p=0.0003).  Patients with all the risk factors (19-repeat allele, C allele of rs12423791, or C-T haplotype) had a significantly shorter cancer specific survival than those with 0-2 of the risk factors (p=0.0003). |
| Johansson | 2007 | To investigate what extent genetic variation in the IGF-I gene is related to prostate cancer risk | Men with PCa (n=2863)  Controls: men randomly selected from the Swedish population (n=1737) | Genotyping by 5’ nuclease assay | Likelihood-ratio test and permutation testing | Common haplotypes in the block covering the 3’region of the IGF1 gene showed significant global association with prostate cancer risk (p = 0.004), with TCC haplotype giving an odds ratio of 1.46 (95% CI 5 1.15–1.84, p = 0.002). |

| **Study** | **Year** | **Experiments** | **Samples** | **Experimental procedures** | **Statistical analysis** | **Results** |
| --- | --- | --- | --- | --- | --- | --- |
| **IGF-I (Continued)** | | | | | | |
| Chang | 2013 | To investigate the association of 4 common SNPs in IGF-I and IGF-IR with age, PSA, Gleason score, surgical margin, lymph node metastasis and PSA recurrence | 320 localised prostate cancer patients receiving radical prostatectomy | Genomic DNA was extracted from peripheral blood of patients.  Genotyping (Sequenom iPLEX MALDI-TOF mass spectrometry | Logistic regression analyses and Cox proportional hazards regression: association of individual SNP alleles, genotypes and haplotypes with clinic pathological characteristics  Multi-factor dimensionality reduction (MDR) analysis: interaction between SNPs and PSA recurrence. | IGF-I rs2946834 alleles/genotypes and an IGF-I specific haplotype AT, containing the minor allele of rs2946834, were associated with higher risk of having advanced-stage prostate cancer (OR: 1.58; 95 % CI: 1.05–2.38; p= 0.027).  IGF-I haplotype AT was associated with an increased risk of having positive surgical margin after RP (OR, 1.65; 95 % CI:1.06–2.58; p= 0.03). |
| **IGF-II** | | | | | | |
| Lai | 2005 | Investigate whether polymorphism of IGF-II could be used as a genetic marker for risk of PCa. | Patients with PCa (n=96); Controls: Healthy male volunteers from the same geographic area (n=121). | PCR using primers for IGF-II gene exon 9 | Chi-square test | No significant difference between distribution of IGF-II gene C/T polymorphism between the healthy control group and the patients with PCa (p=0.78).  No significant difference in the distribution of the IGF-II gene C/T polymorphism between individuals younger than 70yrs and those older than 71yrs (p=0.5). |
| Hu | 2006 | To determine whether the M6P/IGF-IIR gene is inactivated in PCa. | 43 patients with PCa treated with radical prostatectomy | Regions of tumour, normal prostate and PIN were identified and cells were excised by laser capture microdissection.  DNA segments amplified with PCR | Pearson chi-squared test and ANOVA: difference between groups  Kaplan-Meier curve: disease-free survival | M6P/IGF-IIR gene was polymorphic in 83.7% (36/43) of patients.  41.7% (15/36) of these informative patients had loss of heterozygosity (LOH) in the tumor tissue. 11/15 patients with LOH in malignant tissue also had HG-PIN. Of these 63.6% (7/11) also had LOH in HG-PIN tissue.  There was no significant difference in age, PSA levels, stage, Gleason score, proliferative index, cancer involvement, lymphatic/vascular invasion and perineural invasion between the groups with or without LOH.  No significant difference in disease-free survival between the groups of with or without LOH. |

| **Study** | **Year** | **Experiments** | **Samples** | **Experimental procedures** | **Statistical analysis** | **Results** |
| --- | --- | --- | --- | --- | --- | --- |
| **IGF-II (Continued)** | | | | | | |
| Lui | 2006 | To explore the genomic imprinting of IGF-II in PCa and its correlation to disease progression. | PCa (n=41), BPH (n=27) and normal prostate tissue (n=13) | PCR-RFLP | Fisher’s exact test; t-test; Kaplan-Meier and log rank test | Rates of heterozygote of IGF-II DNA were 70.7% in PCa, 55.5% in BPH and 61.5% in normal prostate tissue group.  Occurrence rate of LOI of IGF-II was higher in PCa than in BPH and normal tissue (p=0.05).  LOI of IGF-II had no correlation to age, PSA, presence of bone metastasis, and cell differentiation before endocrinotherapy.  After androgen blockade, the 1 year progression free survival rate was lower in patient s with LOI of IGF-II tan in patients without LOI of IGF-I (p=0.04). |
| Fu | 2008 | Determined whether normal imprint is altered for the *IGF-2* gene with aging in the prostate. | Prostate tissues from C57/B6 mice (containing a Cast I*GF-2-H19* allele)  Histologically normal human prostate specimens from men without cancer (n=40) or men with cancer (n=25). | qPCR, DNA methylation sequencing, ChIP | Not stated | Significant loss of imprinting (LOI) for *IGF-2* in the dorsolateral prostate (DLP) beginning at 11 months compared to young sexually mature mice (3 months). LOI is associated with increase in *IGF-2* mRNA and protein expression.  siRNA mediated down-regulation of CTCF induced LOI in prostate cells.  No alteration in LOI of IGF-2 in the ventral prostate or non prostate tissues.  LOI in histologically normal prostate tissues from men with cancer is significantly greater than men without cancer (p=0. 02). |
| Paradowska | 2009 | Analysed DNA methylation and histone modifications in the differentially methylated region (DMR) of IGF-II/H19 in benign prostate hyperplasia (BPH) and prostate carcinoma (PCa). | 30 prostate radical prostatectomy or cystoprostatectomy  Control: 17 BPH surrounding tumors | Sodium bisulfite treatment and DNA sequencing of genomic DNA  The methylation pattern of 17 CpGs within 227 bp of the H19 fragment was characterized from each DNA sample.  ChIP | Mann-Whitney test | All BPH samples demonstrated >80% methylation of CpGs while 41% of CpGs were methylated in 9 out of 30 PCa specimens. Statistically significant differences in the methylation state was found between PCa and BPH groups, especially in the differentially methylated region (DMR) of H19 (p<0.0001) and in the imprinting control region (ICR) (p=0.0034), which corresponds to CTCF binding domain.  ChIP assay revealed that dimethyl H3K9 is associated with the ICR of IGF-II/H19 in BPH, but not in PCa (p<0.0001). |

| **Study** | **Year** | **Experiments** | **Samples** | **Experimental procedures** | **Statistical analysis** | **Results** |
| --- | --- | --- | --- | --- | --- | --- |
| **IGF-II (Continued)** | | | | | | |
| Bhusari | 2011 | To define whether IGF-2 LOI in histologically normal prostate tissues in relationship to tumour foci and gene expression | Prostatectomy samples containing tumour and associated normal tissue (n=18)  Controls: Normal prostate samples without any associated tumours from age-matched men. | Fluorescent primer extension (FluPE), qPCR, DNA methylation analysis | Spearman’s correlation; two-tailed t-test | Marked IGF-2 LOI in adjacent tumour associated tissues (39 $\pm$3.1%) but not in tissues distant (38 $\pm$5.3%) from tumour foci (45$\pm$2.9%).  IGF-2 imprinting correlated with IGF-2 expression in adjacent tumour associated tissues but not within the tumour foci.  Hypomethylation of IGF-2 DMRO region correlated with decreased IGF-2 expression in tumours (p<0.01).  The expression of IGF-2 and H19 gene were increased in adjacent and distant tissues compared to tumours (p<0.05). |
| Belharazem | 2012 | To investigate levels of IGF-II protein levels and IGF-II 820G/A genotype whether loss of imprinting (LOI) of IGF-II in normal circulating peripheral blood lymphocytes can predict increased PCa risk | 113 blood samples of patients with a history of radical prostatectomy for PCa  Controls: volunteer blood donors | ELISA: serum IGF-II and IGFBP-3 levels  Restriction-fragment length polymorphism: heterozygosity at ApaI-sensitive 820G>A locus on exon 7 of IGF-II gene  LOI or retention of imprinting (ROI): cDNA amplification from heterozygous cases by a nested RT-PCR method.  Bisulfite-DNA sequencing: Methylation status of IGF-II imprinting control region (ICR) | Chi-square test, Mann–Whitney U test and Spearman’s test | Among men with a history of PCa, the 820G/A genotype was significantly more frequent than among healthy control persons (50.5% in PCa patients vs 43% in controls; OR 1.92; 95% CI: 1.22–3.02, p=0.005).  LOI in PCa patients was significantly more frequent (16/41 cases (39%), P=0.03).  Higher degree of methylation in samples with LOI than in ROI in both PCa patients and controls.  All ICRs in samples of RPE patients, irrespective of the imprinting status, showed a higher degree of methylation compared with control samples. |

| **Study** | **Year** | **Experiments** | **Samples** | **Experimental procedures** | **Statistical analysis** | **Results** |
| --- | --- | --- | --- | --- | --- | --- |
| **IGFBP-2** | | | | | | |
| Hu | 2006 | To determine whether the M6P/IGF-IIR gene is inactivated in PCa. | 43 patients with PCa treated with radical prostatectomy | Regions of tumour, normal prostate and PIN were identified and cells were excised by laser capture microdissection.  DNA segments amplified with PCR | Pearson chi-squared test and ANOVA: difference between groups  Kaplan-Meier curve: disease-free survival | M6P/IGF-IIR gene was polymorphic in 83.7% (36/43) of patients.  41.7% (15/36) of these informative patients had loss of heterozygosity (LOH) in the tumor tissue. 11/15 patients with LOH in malignant tissue also had HG-PIN. Of these 63.6% (7/11) also had LOH in HG-PIN tissue.  No significant difference in age, PSA levels, stage, Gleason score, proliferative index, lymphatic/vascular invasion, and disease-free survival between the groups with or without LOH. |
| Paradowska | 2009 | Analysed DNA methylation and histone modifications in the differentially methylated region (DMR) of IGF-II/H19 in benign prostate hyperplasia (BPH) and prostate carcinoma (PCa). | 30 prostate radical prostatectomy or cystoprostatectomy  Control: 17 BPH surrounding tumors | Sodium bisulfite treatment and DNA sequencing of genomic DNA  The methylation pattern of 17 CpGs within 227 bp of the H19 fragment was characterized from each DNA sample.  ChIP | Mann-Whitney test | All BPH samples demonstrated >80% methylation of CpGs while 41% of CpGs were methylated in 9 out of 30 PCa specimens. Statistically significant differences in the methylation state was found between PCa and BPH groups, especially in the differentially methylated region (DMR) of H19 (p<0.0001) and in the imprinting control region (ICR) (p=0.0034), which corresponds to CTCF binding domain.  ChIP assay revealed that dimethyl H3K9 is associated with the ICR of IGF-II/H19 in BPH, but not in PCa (p<0.0001). |
| **IGFBP-3** | | | | | | |
| Okugi | 2006 | Investigated whether the methylation status of IGFBP-3 promoter in prostate tissues influences the progression and prognosis of prostate cancer. | PCa patients (n=38)  Controls: BPH (n=57) | Bisulfite modification and methylation-specific PCR of genomic DNA  Hep-methylation status and NSCLC-methylation status | Chi-squared test: distribution of methylation frequency of IGFBP-3 promoter region  Unconditional logistic regression: OR and 95% CI | No significant difference in the methylation frequency of the IGFBP-3 promoter between cases and controls (OR:1.53; 95% CI: 0.85-5.56; p=0.15) for Hep-primer method; OR=3.24; 95% CI: 0.46-15.42; p=0.21 for NSCLC-primer method).  No statistically significant association between the hypermethylation of IGFBP-3 promoter and clinical stage or Gleason score. |

| **Study** | **Year** | **Experiments** | **Samples** | **Experimental procedures** | **Statistical analysis** | **Results** |
| --- | --- | --- | --- | --- | --- | --- |
| **IGFBP-3 (Continued)** | | | | | | |
| Perry | 2007 | Investigated the methylation pattern of IGFBP-3 in benign, pre-invasive and cancerous prostate tissues | 40 prostatectomy specimens; 39 primary tumours (USA); 14 HG-PIN lesions (from 79 patients) with PCa.  Control: histologically normal adjacent tissue from PCa patients and BPH lesions | Bisulfite modification of genomic DNA and quantitative methylation specific PCR | Fisher’s exact test  Kruskal-Wallis one way ANOVA test and Wilcoxon-matched pairs test | IGFBP3 promoter was completely unmethylated in the histologically normal prostate samples.  In the BPH samples, the frequencies of IGFBP-3 promoter methylation were significantly less than detected in tumours (P<0.0001).  IGFBP-3 promoter methylation was only detected in HGPIN samples from patients whose adjacent tumour was also methylated The frequency of IGFBP3 methylation in HGPIN was not statistically different from tumour (p=0.383).  Methylation of IGFBP-3 promoter was detected in significantly more in tumours with Gleason score $\geq$7, than $\leq$6 (p=0.01), but was not significantly correlated with TNM classification or PSA level. |
| Johansson | 2009 | Analysed genetic variation within genes coding for IGFBP in relation to prostate cancer incidence and survival. | Genotyping analysis: PCa cases (n=2774); Controls: men randomly selected from the Swedish population (n=1736) | Genotyping by 5’ nuclease assay  ELISA: plasma total and intact IGFBP-3 levels | Conditional logistic regression: odds ratio  Cox proportional hazards: survival analysis  Likelihood ratio test | No association between the IGFBP-3 genetic variants and prostate cancer incidence or survival.  The rare allele of the IGFBP-3 SNP rs2854744 was associated with elevated plasma levels of total IGFBP-3 (Ptrend =9x 10^-8^), but not intact IGFBP3 (Ptrend=0.16). |

Note: PCa: Prostate cancer; HG-PIN: High grade prostatic intraepithelial neoplasia; IHC: immunohistochemistry; NAP: normal adjacent counterpart; SEM: standard error of mean; qPCR: quantitative reverse-transcription polymerase chain reaction; BPH: Benign prostatic hyperplasia; ChIP: Chromatin immunoprecipitation; ab: antibody; ELISA: enzyme-linked immunosorbent assay; PSA: prostate specific antigen; TURP: transurethral resection of the prostate; MALDI-TOF: matrix-assisted laser desorption/ionization-time of flight; PCR: polymerase chain reaction; DRE: digital rectal examination; OR: Odds ratio; CI: confidence interval ; PCR-RFLP: Polymerase chain reaction-based restrictive fragment length polymorphism

Supplementary Table 5: Studies investigating circulating levels of IGF system and prostate cancer included as supporting evidence.

| **Study** | **Year** | **Experiments** | **Samples** | **Experimental procedures** | **Statistical analysis** | **Results** |
| --- | --- | --- | --- | --- | --- | --- |
| **IGF-I** | | | | | | |
| Tricoli | 1999 | To determine the overall plasma levels of IGF-I in men at higher risk of PCa development and to investigate the relationships between demographic and IGF-I levels | 105 men ( 63 African American (AA) and 42 White) with no personal history of PCa but have at least one 1^st^ degree relative diagnosed with PCa. | ELISA | Wilcoxon test; Spearman correlation coefficient; linear regression; | Mean plasma level of IGF-I was not significantly different between AA (162.3ng/ml) and white (172.1ng/ml) men (p=0.42).  Inverse relationship between IGF-I plasma levels and age (p=0.008). |
| Baffa | 2000 | Relation between serum IGF-I and PCa | 57 patients who underwent radical prostatectomy for adenocarcinoma. Serum samples collected before radical prostatectomy (T0) or 6 months after radical prostatectomy (T6)  Controls: 39 age-matched controls | Active IGF-I Elisa Kit  (Diagnostic systems Lab) | Welch’s t-test and paired t-test | Serum IGF-I levels were lower in patients with PCa (124.6$\pm$ 58.2ng/ml) compared to the control (157.5$\pm$ 70.8ng/ml) (p=0.0192).  Mean serum IGF-I levels for case patients at T0 (124.91$\pm$ 58.6ng/ml) was lower than patients in the T6 group (148.49$\pm$ 57.2ng/ml) (p=0.0056). |
| Shariat | 2000 | Investigate pre-operative levels of IGF-I plasma levels in patients with clinically localize PCa | 120 patients who underwent radical prostatectomy for clinically localized prostatic adenocarcinoma.  Control: Healthy patients without PCa (n=20). (No prior history of any cancer or chronic disease, a normal digital rectal examination, and a PSA<2ng/ml). | DSL-105600 Active-IGF-I ELISA assay | ANOVA: means among patient groups.  Spearman’s rank correlation coefficient: compare ordinal and continuous variables  Logistic regression multivariate analysis of binary outcomes. | From univariate analysis, pretreatment IGF-I levels did not correlate with age (p=0.89), preoperative PSA (p=0.28), pathologic Gleason score (p=0.49) and pathologic stage (p=0.56).  In both a univariate and a multivariate logistic regression analysis that included preoperative IGF-I, preoperative PSA, clinical stage, and biopsy Gleason score, IGF-I levels did not predict organ- confined disease (P =0.56, P = 0.4165, respectively). |
| Shariat  (Cont.) |  |  |  |  | Kaplan-Meier Curves: Survival analysis  Cox Proportional hazards: time to recurrence | No significant difference for PSA progression-free survival between patients with high IGF-I levels ($\geq$151.1ng/ml) and patients with low IGF-I levels (<151.1ng/ml) (P= 0.76).  IGF-I levels in radical prostatectomy patients were not significantly higher than those in healthy subjects or in patients with metastatic disease (p=084). |
| Stattin | 2001 | To investigate if increased plasma leptin levels are associated with development of PCa. | PCa Patients (n=149); Controls: Matched men without cancer (n=298) | Immunoradiometric assays | Pearson correlation analysis; Univariate and multivariate logistic regression analysis | Adjustments for IGF-I or IGFBP 1-3, (either as continuous or categorical variables) in separate and combined multivariate models did not attenuate the increased risk associated with moderately elevated leptin levels. |
| **Study** | **Year** | **Experiments** | **Samples** | **Experimental procedures** | **Statistical analysis** | **Results** |
| **IGF-I (Continued)** | | | | | | |
| Yu | 2001 | To determine changes in IGF-I, IGFBP-2 and IGFBP-3 levels in serial post-operative serum samples from PCa patients with and without relapse | PCa Patients (n=148)  Patients who developed recurrence (n=38)  Controls: patients who remained in remission (n=40) | ELISA to measure IGF-I, IGFBP-2 and IGFBP-3 in serum samples | Wilcoxon rank-sum test; Friedman test; Page’s L test; generalized linear model (GLM) | No difference in IGF-I levels between cases and controls (p=0.28). |
| Latif | 2002 | To assess the relationships between IGF-I and PCa disease stage | Patients with BPH (n=17), stage T1/T2 PCa (n=15), T3/T4 cancer (n=16) and metastatic PCa (n=12) | Immuno-enzymometric assay: IGF-I  ELISA: IGFBP-3 | Anova (Kruskal-Wallis) and Mann-Whitney U-test | IGF-I concentrations were similar between patients with BPH and those with cancer.  No correlation between age and IGF-I concentration. |
| Oliver | 2003 | Assess whether serum levels of IGFs and IGFBPs were associated with grade, serum PSA and clinical stage. | 224 men (50-70yrs) with screen-detected prostate cancer identified via population-based case-finding in three UK centres  All had total PSA $\geq$3ng/ml  No healthy controls | IGF-I & -II (ELISA, DSL)  IGFBP-2 (RIA, DSL),  IGFBP-3 (RIA, 'in-house' assay)  the molar ratio of IGF-I:IGFBP-3 (a measure of IGF-I bioavailability) was derived. | Not stated | Geometric mean levels of IGF-I did not vary by stage or grade but were higher in cases with a higher PSA (PSA 3-5ng/ml geometric mean (CI) IGF-I 126.5ng/ml (121.5-133.0), PSA 20+ng/m1 IGF-I 144.0 (129.0- 159.2), P_trend_=0.05).  The IGF-I:IGFBP-3 molar ratio did not differ by clinical stage, but was significantly higher in cases with higher PSA (PSA 3-5ng/ml geometric mean (CI) molar ratio 19.8% (18.6-21.0), PSA 20+ng/ml molar ratio 23.2% (20.6-26.4), P_trend_=0.03)  The IGF-I:IGFBP-3 molar ratio was higher in men with higher grade tumours, (Gleason <7 geometric mean (CI) molar ratio 19.8% (18.8-21.0), Gleason 2:7 geometric mean (CI) molar ratio 22.3% (20.4-24.2), P=0.03) |
| Woodson | 2003 | To evaluate the association between pre-diagnostic levels of IGF-I and IGFBP-3 and PCa risk in a nested case-control study (RAS)  To examine changes in serum IGF-I and IGFBP-3 levels over time (SSS) | RAS: PCa (n=100); Controls: randomly selected members from cohort  SSS: PCa (n=21); Controls: trial participants who had no cancer diagnosed (except non-melanoma skin cancer) over the full period of study follow-up and had two serum draws at least 1 year apart. | ELISA | Logistic regression and paired t-test | No significant association between prostate cancer risk and either serum IGF-I (OR: 0.52; 95% CI, 0.23–1.16) after adjusting for age, BMI, intervention group assignment.  Ratio of IGF-I: IGFBP-3 had borderline significant inverse association with PCa risk (p=0.06).  The cases had an average 18% increase in serum IGF-I levels compared with a 4% decrease among controls (P=0.02). |

| **Study** | **Year** | **Experiments** | **Samples** | **Experimental procedures** | **Statistical analysis** | **Results** |
| --- | --- | --- | --- | --- | --- | --- |
| **IGF-I (Continued)** | | | | | | |
| Tu | 2004 | To investigate the levels of IGF-1 and IGFBP-3 in bone marrow aspirates and plasma samples of men with advanced prostate cancer | 42 patients with widespread bone metastases (n-22) and without bone metastasis (n=20) | ELISA | Not stated | Levels of IGF-I and IGFBP-3 were lower in bone marrow supernatant than in plasma.  Bone marrow supernatant: median IGFBP-3 levels were significantly lower in the Met group (834.5ng/ml) than in the Non-Met group (1650ng/ml) (p=0.0001).  No correlation between IGF-I levels and metastasis. |
| Nam | 2005 | To determine whether high serum IGF-I levels are associated with precancerous lesions of the prostate  To compare serum IGF-I and IGFBP-3 levels between men with PCa and those without cancer or with HG-PIN | Cases: Patients with HGPIN from prostate biopsy.  Controls: no evidence of adenocarcinoma of the prostate or HG-PIN ( 2 or more negative biopsies) | ELISA | Not stated | The mean serum IGF-I level for patients with HGPIN (130.2 ng/mL) was significantly higher than for controls (118.8 ng/mL, P = 0.01).  The crude odds ratio for having HGPIN for patients with the highest quartile of serum IGF-I level compared with the lowest quartile group was 1.95 [95% confidence interval (CI), 1.0-3.7; P = 0.04)  The mean IGF-I level for patients with cancer was 119.4 ng/mL (n = 483) and was not significantly different from the 205 patients in the control group (118.8 ng/mL, P = 0.85). |
| Woongeol | 2007 | Case-control study to investigate the association between serum IGF-I and IGFBP-3 levels and prostate cancer risk | 330 men (165 cases treated by radical prostatectomy and 165 healthy age-matched controls). | Not stated | Conditional logistic regression | Risk of PCa not related to IGF-I and IGF-1:IGFBP-3 molar ratio. |
| Ito | 2009 | IGF-1 and IGFBP-3 kinetics | 78 cases with baseline PSA<4ng/ml and diagnosed with PCa after undergoing at 3 times of screening  Control: 156 age-adjusted and baseline PSA-adjusted men without prostate cancer and screened at least 3 times. Men with PSA velocity <0.2ng/ml/yr was recommended for selection. | Serum IGF-1 and IGFBP-3 were measured using serum samples at initial, intermediate and last screening visits in each participant. | Not stated | There was no significant difference in the baseline IGF- 1, baseline IGFBP-3, IGF-1 velocity and IGFBP-3 velocity between the case and the control group. |

| **Study** | **Year** | **Experiments** | **Samples** | **Experimental procedures** | **Statistical analysis** | **Results** |
| --- | --- | --- | --- | --- | --- | --- |
| **IGF-I (Continued)** | | | | | | |
| Mucci | 2010 | To investigate the levels of IGF-I and IGFBP-3 in plasma samples from PCa patients | 545 incident cases; Controls: 545 matched-controls | ELISA | Conditional logistic regression models | No association between free IGF-I and prostate cancer risk (RR, 0.9; 95% CI: 0.6-1.3) |
| Rowlands | 2012 | Investigated associations of circulating IGF-I, IGF-II, IGFBP-2 and IGFBP-3 with all-cause and PCa mortality in men with clinically identified PCa, stratified by whether localised (stage T1 or T2) or advanced (T3, T4, N1 or M1) at diagnosis. | 396 men with PCa | In-house radioimmunoassay (RIA): For IGF-I, IGF-II and IGFBP-3  ELISA: total IGF-I, IGF-II or IGFBP-3 | Age-adjusted linear regression models, likelihood ratio test and Cox regression hazards regression | In men with advanced cancer, IGF-I was positively associated (HR 1.20; 95% CI: 0.96, 1.49; p = 0.11) and IGFBP-3 was inversely associated (HR 0.84; 95% CI: 0.70, 1.01; p = 0.07) with all-cause mortality after controlling for age, treatment status, smoking, prostate-specific antigen and Gleason grade at diagnosis.  IGF-I was positively associated with prostate cancer mortality in advanced cases (HR 1.23; 95% CI: 0.94, 1.62; p = 0.13).  In advanced cancers, associations of IGF-I with all-cause (HR 1.68; 95% CI: 1.28, 2.23; p<0.001) and prostate cancer–specific (HR 1.59; 95% CI: 1.11, 2.28; p = 0.01) mortality strengthened (and were conventionally statistically significant) and controlling for IGFBP-3. |
| **IGF-II** | | | | | | |
| Oliver | 2003 | Assess whether serum levels of IGFs and IGFBPs were associated with grade, serum PSA and clinical stage. | 224 men (50-70yrs) with screen-detected prostate cancer identified via population-based case-finding in three UK centres. All had total PSA $\geq$3ng/ml  No healthy controls | IGF-I & -II (ELISA, DSL); IGFBP-2 (RIA, DSL); IGFBP-3 (RIA, 'in-house' assay)  Molar ratio of IGF-I:IGFBP-3 (a measure of IGF-I bioavailability) was derived. | Not stated | After adjustment for age and centre, geometric mean levels of IGF-II did not differ by disease stage, grade or PSA. |
| Belharazem | 2012 | To investigate levels of IGF-II protein levels and IGF-II 820G/A genotype whether loss of imprinting (LOI) of IGF-II in normal circulating peripheral blood lymphocytes can predict increased PCa risk | 113 blood samples of patients with a history of radical prostatectomy for PCa  Controls: volunteer blood donors | ELISA: serum IGF-II and IGFBP-3 levels | Chi-square test, Mann–Whitney U test and Spearman’s test | In contrast to controls, IGF-II levels in all PCa patients were increased and appeared uncoupled from the imprinting status (p=0.9).  IGF-II protein levels both in patients and in controls were tightly correlated with IGFBP-3 levels (r=0.8; p<0.0001). |

| **Study** | **Year** | **Experiments** | **Samples** | **Experimental procedures** | **Statistical analysis** | **Results** |
| --- | --- | --- | --- | --- | --- | --- |
| **IGFBP-2** | | | | | | |
| Yu | 2001 | To determine changes in IGF-I, IGFBP-2 and IGFBP-3 levels in serial post-operative serum samples from PCa patients with and without relapse | PCa Patients (n=148)  Patients who developed recurrence (n=38)  Controls: patients who remained in remission (n=40) | ELISA to measure IGF-I, IGFBP-2 and IGFBP-3 in serum samples | Wilcoxon rank-sum test; Friedman test; Page’s L test; generalized linear model (GLM) | Lower serum levels of IGFBP-2 in cases than in controls (p<0.05).  In sequential samples, IGFBP-2 levels increased over time in controls (p=0.014) but not in cases (p=0.53). |
| Oliver | 2003 | Assess whether serum levels of IGFs and IGFBPs were associated with grade, serum PSA and clinical stage. | 224 men (50-70yrs) with screen-detected prostate cancer identified via population-based case-finding in three UK centres. All had total PSA $\geq$3ng/ml  No healthy controls. | IGF-I & -II (ELISA, DSL); IGFBP-2 (RIA, DSL); IGFBP-3 (RIA, 'in-house' assay)  Molar ratio of IGF-I:IGFBP-3 (a measure of IGF-I bioavailability) was derived. | Not stated | After adjustment for age and centre, geometric mean levels of IGFBP-2 did not differ by disease stage, grade or PSA. |
| **IGFBP-3** | | | | | | |
| Smith | 1999 | Compared concentrations of IGFBP-3 and PSA in bone metastases and measured serum IGFBP-3 in patients with changing PSA concentrations. | Metastatic bone tissues from patients with PCa (n=6) and patients with breast cancer (n=5) | Western blot: IGFBP-3 concentrations in metastatic tissue  ELISA: serum IGFBP-3 levels | Mann-Whitney test | IGFBP-3 tissue concentrations in PSA-positive bone metastases from patients with PCa were lower compared to PSA-negative bone metastases from patients with breast cancer (p=0.0081).  Inverse correlation between serum PSA and IGFBP-3 concentrations in patients with PCa during period of therapeutic response or disease progression. |
| Tricoli | 1999 | To determine the overall plasma levels of IGF-I in men at higher risk of PCa development and to investigate the relationships between demographic and IGF-I levels | 105 men ( 63 African American (AA) and 42 White) with no personal history of PCa but have at least one 1^st^ degree relative diagnosed with PCa. | ELISA | Wilcoxon test; Spearman correlation coefficient; linear regression; | Mean plasma level of IGFBP-3 was lower in AA (2789 ng/ml) than in white (3216ng/ml) men (p=0.005).  No correlation between IGFBP-3 plasma levels and age. |
| Yu | 2001 | To determine changes in IGF-I, IGFBP-2 and IGFBP-3 levels in serial post-operative serum samples from PCa patients with and without relapse | PCa Patients (n=148)  Patients who developed recurrence (n=38)  Controls: patients who remained in remission (n=40) | ELISA to measure IGF-I, IGFBP-2 and IGFBP-3 in serum samples | Wilcoxon rank-sum test; Friedman test; Page’s L test; generalized linear model (GLM) | Lower serum levels of IGFBP-3 in cases than in controls (p<0.05). |

| **Study** | **Year** | **Experiments** | **Samples** | **Experimental procedures** | **Statistical analysis** | **Results** |
| --- | --- | --- | --- | --- | --- | --- |
| **IGFBP-3 (Continued)** | | | | | | |
| Latif | 2002 | To assess the relationships between IGF-I and PCa disease stage | Patients with BPH (n=17), stage T1/T2 PCa (n=15), T3/T4 cancer (n=16) and metastatic PCa (n=12) | Immuno-enzymometric assay: IGF-I  ELISA: IGFBP-3 | Anova (Kruskal-Wallis) and Mann-Whitney U-test | IGFBP-3 concentrations were similar between patients with BPH and those with cancer.  Age was correlated with IGFBP-3 concentrations (r=-0.4; p=0.008). |
| Oliver | 2003 | Assess whether serum levels of IGFs and IGFBPs were associated with grade, serum PSA and clinical stage. | 224 men (50-70yrs) with screen-detected prostate cancer identified via population-based case-finding in three UK centres. All had total PSA $\geq$3ng/ml  No healthy controls | IGF-I & -II (ELISA, DSL); IGFBP-2 (RIA, DSL); IGFBP-3 (RIA, 'in-house' assay);  Molar ratio of IGF-I:IGFBP-3 (a measure of IGF-I bioavailability) was derived. | Not stated | After adjustment for age and centre, geometric mean levels of IGFBP-3 did not differ by disease stage, grade or PSA. |
| Tu | 2004 | To investigate the levels of IGF-1 and IGFBP-3 in bone marrow aspirates and plasma samples of men with advanced prostate cancer. | 42 patients with widespread bone metastases (n-22) and without bone metastasis (n=20) | ELISA | Not stated | IGFBP-3 levels in the bone marrow supernatant inversely correlated with serum alkaline phosphatase (p=0.0003) and PSA (p=0.02). |
| Nam | 2005 | To determine whether high serum IGF-I levels are associated with precancerous lesions of the prostate  To compare serum IGF-I and IGFBP-3 levels between men with PCa and those without cancer or with HG-PIN | Cases: Patients with HGPIN from prostate biopsy.  Controls: no evidence of adenocarcinoma of the prostate or HG-PIN ( 2 or more negative biopsies) | ELISA | Not stated | The mean IGFBP-3 level was slightly higher for patients with HGPIN (2,393.9 ng/mL) compared with controls (2,276.0, P = 0.06).  The crude odds ratio for having HGPIN for patients with the highest quartile of serum IGFBP-3 level compared with the lowest quartile group was 2.04 (95% CI, 1.1- 3.9; P = 0.03).  The mean IGFBP-3 level for patients with cancer (2,222.7 ng/mL) was also not significantly different to the control group (2,276.0 ng/mL, P = 0.26). |
| Woongeol | 2007 | Case-control study to investigate the association between serum IGF-I and IGFBP-3 levels and prostate cancer risk | 330 men (165 cases treated by radical prostatectomy and 165 healthy age-matched controls). | Not stated | Conditional logistic regression | Strong inverse association between IGFBP-3 and PCa risk.  Men in highest quartile of IGFBP-3 had 88% reduced risk of PCa compared with men in the lowest quartile (OR=0.12; 95% CI: 0.05-0.64; p<0.01).  48% and 76% reduced risk of aggressive prostate cancer in 3^rd^ and 4^th^ quartile of IGFBP-3 levels compared to 1^st^ quartile. |

Note: PCa: Prostate cancer; HG-PIN: High grade prostatic intraepithelial neoplasia; IHC: immunohistochemistry; NAP: normal adjacent counterpart; SEM: standard error of mean; qPCR: quantitative reverse-transcription polymerase chain reaction; BPH: Benign prostatic hyperplasia; ChIP: Chromatin immunoprecipitation; ab: antibody; ELISA: enzyme-linked immunosorbent assay; PSA: prostate specific antigen; TURP: transurethral resection of the prostate; MALDI-TOF: matrix-assisted laser desorption/ionization-time of flight; PCR: polymerase chain reaction; DRE: digital rectal examination; OR: Odds ratio; CI: confidence interval

Supplementary Table 6 - GRADE assessment of studies of milk and IGF-I levels

| **Quality assessment** | **Rating** | **Adjustment to rating** | **Notes** |
| --- | --- | --- | --- |
| No of studies/starting rating | 4 human RCTs, 1 intervention study and 24 observational studies | 2 | Larger number of observational studies with a small number of RCTs, therefore start with score of 2 |
| **Factors decreasing confidence** | | | |
| Limitations in study design (risk of bias) | Not serious | 0 | Most studies are moderate or unclear RoB |
| Inconsistency | Not serious | 0 | Just one outlier, but can be explained due to outcome being measured 65 years after exposure |
| Indirectness | Not serious | 0 | All studies looked at the effect of milk/dairy products on IGF1 levels |
| Imprecision | Not serious | 0 | Many large studies |
| Publication bias | Serious | -1 | Although not clearly evident there is likely to be publication bias in this area of research |
| **Factors increasing confidence** | | | |
| Strength of association | Substantial | +1 | Combined p-value is very low. |
| Dose-response |  | 0 | Studies were not able to examine this robustly (RCTs based on a single dose/intervention, or food frequency questionnaires) |
| Confounders likely to minimise the effect |  | 0 | Unable to rule out confounding, may play a role |
| Final numerical rating of quality of evidence | | 2 | |
| Statement of quality of evidence | | There is currently a low level of evidence linking milk with IGF-I levels and this suggests a positive association. | |

Supplementary Table 7 - GRADE assessment of studies of milk and IGF-II levels

| **Quality assessment** | **Rating** | **Adjustment to rating** | **Notes** |
| --- | --- | --- | --- |
| No of studies/starting rating | 3 observational studies | 2 | Only 3 studies all observational, although large population sizes |
| **Factors decreasing confidence** | | | |
| Limitations in study design (risk of bias) | Not serious | 0 | 1 low, 2 unclear |
| Inconsistency | Not serious | 0 | Difficult to determine due to the small number of studies, but no strong evidence of inconsistency |
| Indirectness | Not serious | 0 | All studies looked at the effect of milk/dairy products on IGFII levels |
| Imprecision | Serious | -1 | Based on just 3 studies, two of which had wide confidence intervals |
| Publication bias | Serious | -1 | Although not clearly evident there is likely to be publication bias in this area of research |
| **Factors increasing confidence** | | | |
| Strength of association |  | 0 | Strong association but this was in just one study |
| Dose-response |  | 0 | Not robust |
| Confounders likely to minimise the effect |  | 0 | Unable to rule out confounding, may play a role |
| Final numerical rating of quality of evidence | | 1 | |
| Statement of quality of evidence | | There is currently a very low level of evidence linking milk with IGF-II levels and this suggests a positive association. | |

Supplementary Table 8 - GRADE assessment of studies of milk and IGFBP-I levels

| **Quality assessment** | **Rating** | **Adjustment to rating** | **Notes** |
| --- | --- | --- | --- |
| No of studies/starting rating | 2 observational studies | 2 | Only 2 studies both observational |
| **Factors decreasing confidence** | | | |
| Limitations in study design (risk of bias) | Not serious | 0 | 1 low, 1 unclear |
| Inconsistency | Not serious | 0 | Difficult to determine due to the small number of studies, but no strong evidence of inconsistency |
| Indirectness | Not serious | 0 | Both studies looked at the effect of milk/dairy products on IGFBP-I levels |
| Imprecision | Serious | -1 | Based on just 2 studies |
| Publication bias | Serious | -1 | Although not clearly evident there is likely to be publication bias in this area of research |
| **Factors increasing confidence** | | | |
| Strength of association |  | 0 | No evidence of an association in either study |
| Dose-response |  | 0 | No evidence of a dose response |
| Confounders likely to minimise the effect |  | 0 | Unable to rule out confounding, may play a role |
| Final numerical rating of quality of evidence | | 1 | |
| Statement of quality of evidence | | There is currently a very low level of evidence linking milk with IGFBP-1 levels and this suggests no association. | |

Supplementary Table 9 – GRADE assessment of studies of milk and IGFBP-2 levels

| **Quality assessment** | **Rating** | **Adjustment to rating** | **Notes** |
| --- | --- | --- | --- |
| No of studies/starting rating | 3 observational studies | 2 | Only 3 studies all observational |
| **Factors decreasing confidence** | | | |
| Limitations in study design (risk of bias) | Not serious | 0 | 2 low, 1 unclear |
| Inconsistency | Not serious | 0 | Difficult to determine due to the small number of studies, but no strong evidence of inconsistency |
| Indirectness | Not serious | 0 | Both studies looked at the effect of milk/dairy products on IGFBP-2 levels |
| Imprecision | Serious | -1 | Based on just 3 studies |
| Publication bias | Serious | -1 | Although not clearly evident there is likely to be publication bias in this area of research |
| **Factors increasing confidence** | | | |
| Strength of association |  | 0 | Some evidence of a negative association in 2 studies but no evidence of an association in the 3^rd^ |
| Dose-response |  | 0 | No evidence of a dose response |
| Confounders likely to minimise the effect |  | 0 | Unable to rule out confounding, may play a role |
| Final numerical rating of quality of evidence | | 1 | |
| Statement of quality of evidence | | There is currently a very low level of evidence linking milk with IGFBP-2 levels and this suggests a negative association. | |

Supplementary Table 10 – GRADE assessment of studies of milk and IGFBP3 levels

| **Quality assessment** | **Rating** | **Adjustment to rating** | **Notes** |
| --- | --- | --- | --- |
| No of studies/starting rating | 13 studies, 2 of which are RCTs | 2 | A large number of observational studies with a smaller number of RCTs therefore start with score of 2 |
| **Factors decreasing confidence** | | | |
| Limitations in study design (risk of bias) | Not serious | -1 | Most studies have an unclear RoB |
| Inconsistency | Not serious | 0 | Studies seem to be consistent |
| Indirectness | Not serious | 0 | All studies looked at the effect of milk/dairy products on IGFBP-3 levels |
| Imprecision | Serious | 0 | Several studies, some with large sample size (>1000) |
| Publication bias | Serious | -1 | Although not clearly evident there is likely to be publication bias in this area of research |
| **Factors increasing confidence** | | | |
| Strength of association |  | +1 | Combined p-value is very low |
| Dose-response |  | 0 | Studies were not able to examine this robustly (RCTs based on a single dose/intervention, or food frequency questionnaires) |
| Confounders likely to minimise the effect |  | 0 | Unable to rule out confounding, may play a role |
| Final numerical rating of quality of evidence | | 1 | |
| Statement of quality of evidence | | There is currently a very low level of evidence linking milk and IGFBP-3 levels, and this suggests a negative association | |

Supplementary table 11 – GRADE assessment of studies of IGF-I levels and prostate cancer risk

| **Quality assessment** | **Rating** | **Adjustment to rating** | **Notes** |
| --- | --- | --- | --- |
| No of studies/starting rating | many observational studies | 2 | All observational |
| **Factors decreasing confidence** | | | |
| Limitations in study design (risk of bias) | Not serious | 0 | Moderate risk of bias |
| Inconsistency | Not serious | 0 | Studies show some effects in other directions, although prospective studies are mostly homogeneous |
| Indirectness | Not serious | 0 | All studies looked at circulating levels of IGF-I and their association with prostate cancer risk |
| Imprecision | Serious | 0 | Large number of studies some very large |
| Publication bias | Serious | 0 | No evidence of publication bias |
| **Factors increasing confidence** | | | |
| Strength of association |  | 0 | Fairly weak association |
| Dose-response |  | 1 | Some evidence of dose response |
| Confounders likely to minimise the effect |  | 0 | Unable to rule out confounding, may play a role |
| Final numerical rating of quality of evidence | | 3 | |
| Statement of quality of evidence | | There is currently a moderate level of evidence linking IGF-I levels to prostate cancer risk, and this suggests a positive association | |

Supplementary table 12 – GRADE assessment of studies of IGF-II levels and prostate cancer risk

| **Quality assessment** | **Rating** | **Adjustment to rating** | **Notes** |
| --- | --- | --- | --- |
| No of studies/starting rating | 10 observational studies | 2 | All observational |
| **Factors decreasing confidence** | | | |
| Limitations in study design (risk of bias) | Not serious | 0 | Moderate risk of bias |
| Inconsistency | Not serious | -1 | Studies show strong effects in opposite directions |
| Indirectness | Not serious | 0 | All studies looked at circulating levels of IGF-II and their association with prostate cancer risk |
| Imprecision | Serious | -1 | Small number of studies some with conflicting results |
| Publication bias | Serious | 0 | No evidence of publication bias |
| **Factors increasing confidence** | | | |
| Strength of association |  | 0 | Fairly weak association |
| Dose-response |  | 0 | Possible dose response but inconsistency between studies |
| Confounders likely to minimise the effect |  | 0 | Unable to rule out confounding, may play a role |
| Final numerical rating of quality of evidence | | 1 | |
| Statement of quality of evidence | | There is currently a very low level of evidence linking IGF-II levels with prostate cancer and the evidence suggests a positive association | |

Supplementary table 13 – GRADE assessment of studies of IGFBPI levels and prostate cancer risk

| **Quality assessment** | **Rating** | **Adjustment to rating** | **Notes** |
| --- | --- | --- | --- |
| No of studies/starting rating | 4 observational studies | 2 | All observational |
| **Factors decreasing confidence** | | | |
| Limitations in study design (risk of bias) | Not serious | 0 | Moderate risk of bias |
| Inconsistency | Not serious | -1 | Studies show strong effects in opposite directions |
| Indirectness | Not serious | 0 | All studies looked at circulating levels of IGFBP-I and their association with prostate cancer risk |
| Imprecision | Serious | -1 | Small number of studies overall wide confidence intervals |
| Publication bias | Serious | 0 | No evidence of publication bias |
| **Factors increasing confidence** | | | |
| Strength of association |  | 0 | Fairly weak association |
| Dose-response |  | 0 | Possible dose response but inconsistency between studies |
| Confounders likely to minimise the effect |  | 0 | Unable to rule out confounding, may play a role |
| Final numerical rating of quality of evidence | | 1 | |
| Statement of quality of evidence | | There is currently a very low level of evidence linking IGFBPI levels with prostate cancer risk and this suggests no association | |

Supplementary table 14 – GRADE assessment of studies of IGFBP2 and prostate cancer risk

| **Quality assessment** | **Rating** | **Adjustment to rating** | **Notes** |
| --- | --- | --- | --- |
| No of studies/starting rating | 6 observational studies | 2 | All observational |
| **Factors decreasing confidence** | | | |
| Limitations in study design (risk of bias) | Not serious | 0 | Moderate risk of bias |
| Inconsistency | Not serious | -1 | Studies show strong effects in opposite directions |
| Indirectness | Not serious | 0 | All studies looked at circulating levels of IGFBP-2 and their association with prostate cancer risk |
| Imprecision | Serious | -1 | Small number of studies overall wide confidence intervals |
| Publication bias | Serious | 0 | No evidence of publication bias |
| **Factors increasing confidence** | | | |
| Strength of association |  | 0 | Fairly weak association |
| Dose-response |  | 0 | Possible dose response but inconsistency between studies |
| Confounders likely to minimise the effect |  | 0 | Unable to rule out confounding, may play a role |
| Final numerical rating of quality of evidence | | 1 | |
| Statement of quality of evidence | | There is currently a very low level of evidence linking IGFBP2 levels with prostate cancer. | |

Supplementary table 15 – GRADE assessment of studies of IGFBP3 and prostate cancer risk

| **Quality assessment** | **Rating** | **Adjustment to rating** | **Notes** |
| --- | --- | --- | --- |
| No of studies/starting rating | Many observational studies | 2 | All observational |
| **Factors decreasing confidence** | | | |
| Limitations in study design (risk of bias) | Not serious | 0 | Moderate risk of bias |
| Inconsistency | Not serious | 0 | Some studies show effects in opposite directions, prospective studies are largely homogeneous |
| Indirectness | Not serious | 0 | All studies looked at circulating levels of IGFBP-2 and their association with prostate cancer risk |
| Imprecision | Serious | 0 | Large number of studies some very large |
| Publication bias | Serious | 0 | No evidence of publication bias |
| **Factors increasing confidence** | | | |
| Strength of association |  | 0 | Fairly weak association |
| Dose-response |  | 1 | Some evidence of dose response |
| Confounders likely to minimise the effect |  | 0 | Unable to rule out confounding, may play a role |
| Final numerical rating of quality of evidence | | 3 | |
| Statement of quality of evidence | | There is currently a moderate level of evidence linking IGFBP3 levels with prostate cancer. | |

Supplementary table 16 – GRADE assessment of IGFI and advanced prostate cancer risk

| **Quality assessment** | **Rating** | **Adjustment to rating** | **Notes** |
| --- | --- | --- | --- |
| No of studies/starting rating | observational studies | 2 | All observational |
| **Factors decreasing confidence** | | | |
| Limitations in study design (risk of bias) | Not serious | 0 | Moderate risk of bias |
| Inconsistency | Not serious | -1 | Some outliers and difference between prospective and retrospective studies |
| Indirectness | Not serious | 0 | All studies looked at circulating levels of IGF-I and their association with advanced prostate cancer risk |
| Imprecision | Serious | 0 | Overall estimate quite precise |
| Publication bias | Serious | 0 | No evidence of publication bias |
| **Factors increasing confidence** | | | |
| Strength of association |  | 0 | Fairly weak/no association |
| Dose-response |  | 0 | Possible dose response but inconsistency between studies |
| Confounders likely to minimise the effect |  | 0 | Unable to rule out confounding, may play a role |
| Final numerical rating of quality of evidence | | 1 | |
| Statement of quality of evidence | | There is currently a low level of evidence linking IGFI levels with advanced prostate cancer risk. This evidence suggests a positive association. | |

Supplementary table 17 – GRADE assessment of IGFBP3 and advanced prostate cancer risk

| **Quality assessment** | **Rating** | **Adjustment to rating** | **Notes** |
| --- | --- | --- | --- |
| No of studies/starting rating | observational studies | 2 | All observational |
| **Factors decreasing confidence** | | | |
| Limitations in study design (risk of bias) | Not serious | 0 | Moderate risk of bias |
| Inconsistency | Not serious | 0 | Some difference between prospective and retrospective studies |
| Indirectness | Not serious | 0 | All studies looked at circulating levels of IGFBP3 and their association with advanced prostate cancer risk |
| Imprecision | Serious | 0 | Overall estimate quite precise |
| Publication bias | Serious | 0 | No evidence of publication bias |
| **Factors increasing confidence** | | | |
| Strength of association |  | 0 | Fairly weak/no association |
| Dose-response |  | 0 | No evidence of dose response |
| Confounders likely to minimise the effect |  | 0 | Unable to rule out confounding, may play a role |
| Final numerical rating of quality of evidence | | 2 | |
| Statement of quality of evidence | | There is currently a low level of evidence linking IGFBP3 levels with advanced prostate cancer risk. Overall this evidence suggests no effect | |

Supplementary table 18 – GRADE assessment of animal studies of the IGF pathway and prostate cancer risk

| **Quality assessment** | **Rating** | **Adjustment to rating** | **Notes** |
| --- | --- | --- | --- |
| No of studies/starting rating | Experimental studies | 4 | All experimental |
| **Factors decreasing confidence** | | | |
| Limitations in study design (risk of bias) | Not serious | -1 | All unclear risk of bias |
| Inconsistency | Not serious | 0 | Each study was very different so unable to assess this |
| Indirectness | Not serious | -1 | Components of IGF pathway were knocked out or over expressed to very high levels, no comparable with normal distribution in humans, outcomes were tumour weight rather than incidence |
| Imprecision | Serious | -1 | Small number of animals in each experiment, not able to combine results across studies |
| Publication bias | Serious | -1 | Very likely to be publication bias |
| **Factors increasing confidence** | | | |
| Strength of association |  | 0 | 1 study showed a strong association but not replicated |
| Dose-response |  | 0 | No evidence of dose response |
| Confounders likely to minimise the effect |  | 0 | Unable to rule out confounding, may play a role |
| Final numerical rating of quality of evidence | | 1 | |
| Statement of quality of evidence | | There is currently a very low level of evidence from animal studies linking the IGF pathway with prostate cancer risk. | |

**To aid the adjustment to ratings, high quality evidence scores 4 as a starting rating, low quality evidence scores 2 and very low quality evidence scores 1. The initial starting rating is then adjusted as +1 or -1 based on factors that increase (+1) or decrease (-1) confidence in the quality of evidence. The minimum final numerical rating of quality of evidence is 1.*

Supplementary Box 1. Search strategies used to search MEDLINE and EMBASE (28^th^ March 2014).

1. IGF1.tw

2. IGF-1.tw

3. IGFI.tw

4. IGF-I.tw

5. IGF1A.tw

6. IGF-IA.tw

7. IGF2.tw

8. IGF-2.tw

9. IGF-II.tw

10. IGFII.tw

11. IGF-IB.tw

12. IGF1B.tw

13. Insulin-like growth factor.tw

14. exp Somatomedins/

15. somatomedin*.tw

16. exp Insulin-Like Growth Factor Binding Proteins/

17. exp Receptors, Somatomedins/

18. 1 or 2 or 3 or 4 or 5 or 6 or 7 or 8 or 9 or 10 or 11 or 12 or 13 or 14 or 15 or 16 or 17

19. (prostat* adj3 (neoplas* or cancer or carcinoma or tumo?r)).tw

20. exp Prostatic Neoplasms/

21 19 or 20

22. exp Prostatic Intraepithelial neoplasia/

23. exp Neoplasm Metastasis/

24. exp Neoplasm Invasiveness/

25. 23 or 24

26. 21 and 25

27. 21 or 22 or 26

28. exp Dairy Products/

29. (dairy or milk or cheese* or butter or cream* or yog?urt).tw

30. 28 or 29

31. exp Pasteurization/

32. exp Dairying/

33. exp Food contamination/

34. (food* adj2 contaminat*).tw

35. 33 or 34

36. 30 and 35

37. exp Recombinant Proteins/

38. exp Growth Hormone/

39. exp Cattle/

40. 37 and 38 and 39

41. 30 or 31 or 32 or 36 or 40

42. 18 and 27

43. 42 not exp Therapeutic/

44. 43 not exp Review/

45. 18 and 41

46. 45 not exp Therapeutic/

47. 46 not exp Review/

48. 27 and 41

49. 48 not exp Therapeutic/

50. 49 not exp Review/

51. 18 and 27 and 41

52. 51 not exp Therapeutic/

53. 52 not exp Review/

54. 44 or 47 or 50 or 53

*Note- for EMBASE use Therapy rather than Therapeutic MESH term*

Supplementary Box 2. Search strategies used to search CINAHL (30^th^ March 2014).

1. TI IGF1 or AB IGF1

2. TI IGF-1 or AB IGF-1

3. TI IGFI or AB IGFI

4. TI IGF-I or AB IGF-I

5. TI IGF1A or AB IGF1A

6. TI IGF-IA or AB IGF-IA

7. TI IGF2 or AB IGF2

8. TI IGF-2 or AB IGF-2

9. TI IGF-II or AB IGF-II

10. TI IGFII or AB IGFII

11. TI IGF-IB or AB IGF-IB

12. TI IGF1B or AB IGF1B

13. TI Insulin-like growth factor or AB Insulin-like growth factor

14. (MH “Somatomedins”)

15. TI Insulin-like growth factor binding protein or AB Insulin-like growth factor binding protein

16. TI Somatomedins Receptors or AB Somatomedins Receptors

17. TI Insulin-like growth factor receptor or AB Insulin-like growth factor receptor

18. 1 or 2 or 3 or 4 or 5 or 6 or 7 or 8 or 9 or 10 or 11 or 12 or 13 or 14 or 15 or 16 or 17

19. (MH “Prostatic Neoplasms”)

20. T1 Prostatic Intraepithelial neoplasia or AB Prostatic Intraepithelial neoplasia

21. T1 prostat* N3 (neoplas* or cancer or carcinoma or tumo?r) or AB prostat* N3 (neoplas* or cancer or carcinoma or tumo?r)

22. (MH “Neoplasm Metastasis+”)

23. (MH “Neoplasm Invasiveness”)

24. 22 or 23

25 19 or 21

26. 24 AND 25

27. 25 or 20 or 26

28. (MH “Dairy Products+”)

29. T1 dairy or milk or cheese* or butter or cream* or yog?urt or AB dairy or milk or cheese* or butter or cream* or yog?urt

30. 28 or 29

31. (MH “Pasteurization”)

32. TI Dairying or AB Dairying

33. (MH “Food Contamination+”)

34. 30 AND 33

35. (MH “Recombinant Proteins+”)

36. TI Growth Hormone or AB Growth Hormone

37. (MH “Cattle”)

38. 35 AND 36 AND 37

39. 30 or 31 or 32 or 34 or 38

40. (MH “Therapeutics+”)

41. (MH “Systematic Review”)

42. (MH “Book Reviews”)

43. (MH “Literature Review+”)

44. 41 or 42 or 43

45. 18 AND 27

46. 45 not 40

47. 46 not 44

48. 18 AND 39

49. 48 not 40

50. 49 not 44

51. 27 AND 39

52. 51 not 40

53. 52 not 44

54. 18 AND 27 AND 39

55. 54 not 40

56. 55 not 44

57. 47 or 50 or 53 or 56

Supplementary Box 2. Search strategies used to search BIOSIS (31^st^ March 2014).

1. Topic=(IGF1 or IGF-1 or IGFI or IGF-I or IGF1A or IGF-IA or IGF2 or IGF-2 or IGF-II or IGFII or IGF-IB or IGF1B)

2. Topic=(Insulin-like growth factor)

3. Topic=(Somatomedins)

4. Topic=(Insulin-like growth factor binding proteins)

5. Topic=(Somatomedins Receptors)

6. Topic=(Insulin-like growth factor receptor)

7. 6 OR 5 OR 4 OR 3 OR 2 OR 1

8. Topic=(prostat* neoplas* or prostat* cancer or prostat* carcinoma or prostat* tumo$r)

9. Topic=(prostatic intraepithelial neoplasia)

10. 9 OR 8

11. Topic=(Neoplasm Metastasis or Neoplasm Invasiveness)

12. 11 AND 8

13. 12 OR 10

14. Topic=(Dairy Products)

15. Topic=(dairy or milk or cheese* or butter or cream* or yog$urt)

16. Topic=(pasteurization or dairying)

17. Topic=(Food contamination)

18. 17 AND 14

19. Topic=(Recombinant Proteins)

20. Topic=(Growth Hormone)

21. Topic=(Cattle)

34. 33 AND 32 AND 31

35. 34 OR 30 OR 28 OR 27 OR 26

22. 21 AND 20 AND 19

23. 22 OR 18 OR 16 OR 15 OR 14

24. Topic=(Therapeutics)

25. Topic=(Review)

26. 13 AND 7

27. 26 NOT 24

28. 27 NOT 25

29. 23 AND 7

30. 29 NOT 24

31. 30 NOT 25

32. 23 AND 13

33. 32 NOT 24

34. 33 NOT 25

35. 23 AND 13 AND 7

36. 35 NOT 24

37. 36 NOT 25

38. 37 OR 34 OR 31 OR 28

Supplementary Box 4. Risk of bias protocol.

| **RoB Categories** | **Specific questions used to answer RoB categories** |
| --- | --- |
| ***Milk-IGF (human): case/control*** |  |
| Confounding | Did the authors identify all possible confounding factors? Were these taken into account in the study design and/or analysis? Was there a large difference between the characteristics of cases and controls and if so, were they adjusted for? |
| Selection of participants | Were they selected in an acceptable way? Were they part of a defined population? |
| Missing data | Was the follow-up long enough for the outcome to occur? Was the follow-up complete enough? Were details given of those lost to follow up? E.g., was there a difference in those lost to follow-up compared to those included in the study? |
| Measurement of outcome | Did the authors use objective measurements? Were they validated? Was it accurately measured? |
| Measurement of exposure *(Focus on information recall from participants)* | Did the authors use objective measurements? Were they validated? Were all subjects classified into exposure groups using the same procedure? Was it accurately measured? Did this involve information recall from the participants? |
| Selection of reported results | Was the full protocol available? Were all aims of the study reported? Was the study free of selective reporting? |
| ***Milk-IGF (human): RCT*** |  |
| Sequence generation | Was the way in which participants were selected at random acceptable? |
| Allocation concealment | *Not applicable to this analysis as IGF levels cannot be directly influenced by the participants knowledge of the intervention they are participating in.* |
| Blinding of participants and personnel | *Not applicable to this analysis as IGF levels cannot be directly influenced by the participants knowledge of the intervention they are participating in.* |
| Blinding of outcome assessors | Were the assessors blinded to the analysis they were undertaking? |
| Incomplete data | Was the follow-up long enough for the outcome to occur? Was the follow-up complete enough? Were details given of those lost to follow up? E.g., was there a difference in those lost to follow-up compared to those included in the study? |
| Selective reporting | Was the full protocol available? Were all aims of the study reported? Was the study free of selective reporting? |
| **IGF-PCa (human): all** |  |
| Confounding  *(IGF level studies focused on age & ethnicity; genetics studies focused on age, disease status of controls & ethnicity)* | Did the authors identify all possible confounding factors? Were these taken into account in the study design and/or analysis? Was there a large difference between the characteristics of cases and controls and if so, were they adjusted for? |
| Selection of participants | Were they selected in an acceptable way? Were they part of a defined population? |
| Missing data (cohorts only) | Was the follow-up long enough for PCa to occur? Was the follow-up complete enough? Were details given of those lost to follow up? Was there a difference in those lost to follow-up compared to those included in the study? |
| Measurement of outcome (cohorts only) | Did the authors use objective measurements? Were they validated? Was it accurately measured? |
| Measurement of exposure | Did the authors use objective measurements? Were they validated? Were all subjects classified into exposure groups using the same procedure? Was it accurately measured? |
| Selection of reported results | Was the full protocol available? Were all aims of the study reported? Was the study free of selective reporting? |
| ***IGF-PCa (animal): all*** |  |
| Confounding | Did the authors identify all possible confounding factors? Were these taken into account in the study design and/or analysis? Was there a large difference between the characteristics of cases and controls and if so, were they adjusted for? |
| Departures from intended observations | Were the original aims of the study met? Did the results answer the original study question? |
| Random housing of animals | Were animals housed and kept in the same way to minimise environmental factors? |
| Missing data | Was the follow-up long enough for the outcome to occur? Was the follow-up complete enough? Were details given of those lost to follow up? E.g., was there a difference in those lost to follow-up compared to those included in the study? |
| Measurement of outcome | Did the authors use objective measurements? Were they validated? Was it accurately measured? |
| Random outcome assessment of animals | Were samples consistently taken from experimental and control groups at the same time? |
| Selection of reported results | Was the full protocol available? Were all aims of the study reported? Was the study free of selective reporting? |
